# Novel base-initiated cascade reactions of hemiindigos to produce dipolar γ-carbolines and indole-fused pentacycles†

**DOI:** 10.1039/c9ra07807j

**Published:** 2019-12-13

**Authors:** V. S. Velezheva, O. L. Babii, A. A. Khodak, E. A. Alekseeva, Yu V. Nelyubina, I. A. Godovikov, A. S. Peregudov, K. B. Majorov, B. V. Nikonenko

**Affiliations:** A. N. Nesmeyanov Institute of Organoelement Compounds of Russian Academy of Sciences Vavilova St. 28 119991 GSP-1, Moscow Russia vel@ineos.ac.ru; Laboratory for Immunogenetics, Central Institute for Tuberculosis Moscow Russia

## Abstract

Novel continuous-flow cascade reactions are developed for producing 1,4-diaryl-disubstituted dipolar γ-carbolines 2 that contain a carboxylate group and their two pentacyclic precursors 6, 7 from hemiindigos 1. The nucleophilic and pro-electrophilic chemistry described is new to the hemiindigos 1, and it led to the discovery of antimycobacterial scaffold characteristic of rimino-type pentacycles 6, 7 and potent drug clofazimine. The new scaffold like clofazimine appears to be useful in developing lead agents active against drug-resistant/dormant TB.

## Abstract 2

Based on hemiindigos we developed novel reactions for producing γ-carbolines and their precursors that appeared to be active against MTB.

## Graphical abstract

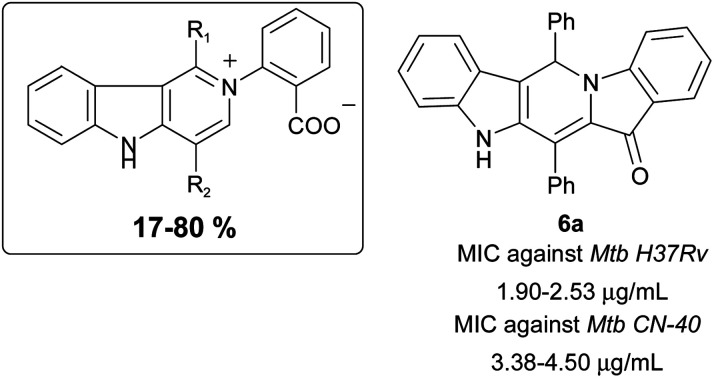

## Introduction

Studies of the cheap dye indigo and its analogs in the last two decades led to the design of novel indigoid photoswitches and to the discovery of new approaches to cascade reactions that contributed largely to materials science and modern organic chemistry.^1^ Base-initiated cascade reactions of indigo using either allyl bromide or propargyl bromide provide rapid and unprecedented one-pot route to a diverse range of unique complex heterocyclic systems that are difficult to access by other means and that exhibit pronounced biological activities for several structures.^2,3^ Cascade synthesis of new bioactive spiroindolinepyrido[1,2-*a*]indolediones from bioactive natural dye indirubin, the 3,2′-bisindole isomer of indigo, was reported.^4^ The base-initiated cascade reactions of indigo with oxiranes and aziridines offered efficient access to dihydropyrazinodiindoles and spiro-oxazocinodiindoles.^5^

Indigoid photoswitches cover a series of various hemithioindigo and hemiindigo chromophores, new studies and applications of those are summarized by C. Petermayer and H. Dube and described by Z. L. Pianowski.^6,7^ The available hemiindigo photoswitches show photochromism in the visible region so that the two isomeric states to be distinguished by the naked eye. Substituted hemiindigos are therefore extremely promising new photoswitches with excellent properties for applications in biology, chemistry, or materials science.^8,9^ The first hemiindigo indoxyl-based 2-phenylmethylideneindoxyl 1a was reported along with indigo in 1883 by A. Baeyer.^10^ However, photochromism of hemiindigos was demonstrated more than a century later.^11^

Natural hemiindigos also include well-known indigoid dyes and natural medicinally important compounds such as indirubin, its family members, and a novel bioactive alkaloid (*E*)-2-[(3′-indole)cyanomethylene]-3-indolinone (isolated from tetraploidy banlangen (Isatis indigotica Fort)).^12^ Indirubins were recognized as inhibitors of glycogen synthase kinase-3β and CDK5/P25, the two protein kinases involved in abnormal tau phosphorylation in Alzheimer's disease.^13^ Indirubins were also identified as potent ATP competitive protein kinase inhibitors.^14^

The indigo-like hemiindigos, 2-arylidene-1*H*-indol-3(2*H*)-ones (2-arylmethylideneindoxyls, or indogenides according to A. Baeyer) 1, were found to possess the challenging structural features as both enamines and α,β-enones show a unique dual nucleophilic and electrophilic reactivity. Earlier, we discovered that hemiindigos enter the ring-opening and ring enlargement reactions of the indoxyl unit^15,16^ and that the exomethylidene hydrogen atom can be replaced by nucleophilic and electrophilic agents.^17^ We also reported, for the first time, a number of unexpected reactions, such as ring conversions and oxidative exomethylidene hydrogen substitution.^15,18^ Hemiindigos are readily available and versatile intermediates in the synthesis of fused indoles,^19–22^ 4-quinolones,^16^ 4-(2-acetylaminobenzoyl)-5-aryl-1,2,3-triazoles,^15^ indoxyl alkaloid analogs,^23^ and pyridazino-[4,3-*b*]indoles exhibiting inhibitory activity against monoamine oxidases A and/or B and *Mycobacterium tuberculosis*.^18^ They also undergo the Diels–Alder and inverse electron demand Diels–Alder reactions^24,25^ as well as diverse cyclocondensations.^26^ Recently, some of us reported a photo-induced cyclodimerization of *N*-alkylhemiindigos (*N*-alkylindogenides) to spiropyrano[3,2-*b*]pseudoindoxyls rather than to cyclobutane adducts, as was reported by M. Hooper *et al.*^27,28^ The spiropseudoindoxyl fragment of pseudoindoxyls is characteristic of many natural alkaloids. Over the last decade, the hemiindigo scaffold has attracted considerable attention from synthetic chemists because of its unique reactivity and relevance to medicinal chemistry due to wide scope of bioactive properties.^29–31^ Our research in the field of cascade reactions began with the discovery of a cationic domino reaction of hemiindigos that afford pentacyclic 3-hydroxyindolenines related to indolenine alkaloids in a diastereoselective fashion under the NMR experiment conditions.^32^ This is the first example of the acid-catalyzed domino reaction that involved homocoupling of hemiindigos 1 followed by a reverse Wagner–Meerwein [1,2] type rearrangement of the intermediate 2-spiropseudoindoxyls.^32^ We also described novel cationic domino reactions of hemiindigos 1 in the absence or in the presence of an external nucleophile.^33^ These reactions provide approaches to the dispiropseudoindoxyl system that represent structural core elements of natural spiroindoxyl alkaloids^23,34^ and a pyrrolo[1,2-*a*]indole core system of biologically active fluorazones.^33^ Interesting results that we have obtained for homodimerization of hemiindigos 1 in acidic media inspired us to investigate related reactions in base-induced conditions that remained unexplored until recently. Here we report novel alkaline-initiated cascade reactions of hemiindigos 1 that lead to 1,4-diaryl-disubstituted zwitterionic γ-carbolinium derivatives 2 with a carboxylate group and two new indole-fused pentacycles 6, 7 as well as to a spiropentacyclic compound 3.

## Results and discussion

**Scheme 1:**
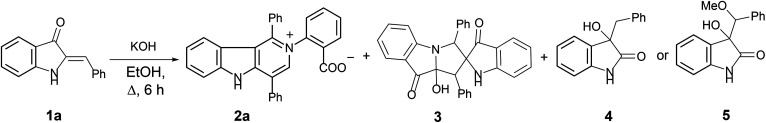
Transformation of hemiindogo 1a in the alkaline conditions.

**Fig. 1:**
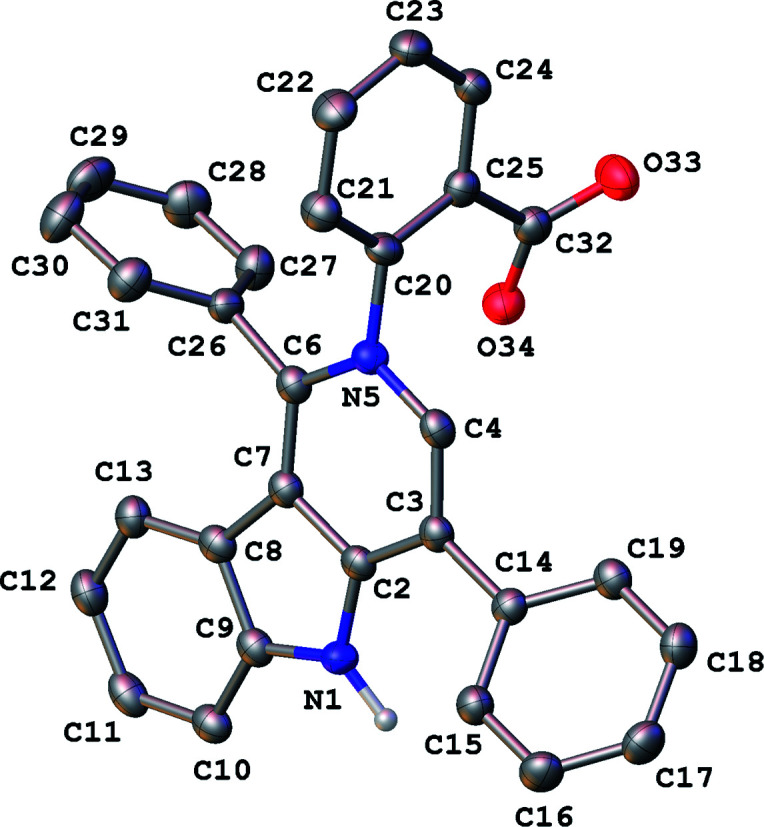
General view of 2a in representation of atoms *via* thermal ellipsoids at 50% probability level.

In our ongoing research^32,33^ into the reactivity of hemiindigos 1, we studied their alkoxide-induced conversions. We observed that brightly colored orange solutions of hemiindigos 1a in neutral media and dark-blue solutions of 1a in alkaline media undergo decoloration during a short-term reflux in the alkaline (KOH) EtOH or MeOH solution. Then we obtained a mixture of γ-carboline 2a, fused spiroindoxyl 3, and 2-indolinone 4 in 2, 14, and 5% yields, respectively, upon reflux of 1a in alkaline EtOH for 6 h (Scheme 1). Another byproduct, 2-indolinone 5, was also formed in 12% isolated yield using MeOH as a solvent. The starting indogenide 1a as well as its analogs 1b–i were obtained in 68–95% yields by condensation of 1-acetylindoxyl and corresponding benzaldehydes upon heating in alkaline EtOH.^27^ The structures of 2, 3 and 5 were deduced from the elemental analyses, mass spectrometry and ^1^H and ^13^C NMR. Compound 4 was obtained previously, and its characteristics are consistent with the published data.^35^ Finally, the structure of 2a (Fig. 1) as well as of 3 (see ESI†) was unambiguously confirmed by single-crystal X-ray diffraction.

The γ-carboline skeleton is widely used in the design of agents against various diseases, and it represents a basic core of many clinically successful drugs. These include antihistaminic drugs mebhydroline (diazolin) and dimebon (latrepirdine) as well as neuroleptic carbidine. The last-two drugs are used in clinical practice in Russia. Flutroline and Gevotroline were successfully tested in clinical trials as neuroleptics. Benzo-λ-carboline alkaloid cryptosanguinolentine with highly conjugated indolenine containing ring system showed antiplasmodial properties.^36^ Various types activities of γ-carbolines, such as neuropharmacological, antihistamine, anti-inflammatory, anticancer, antiviral, antibacterial, antifungal and antimalarial activity, were found in the recent years.^37–39^ Many methods for the synthesis of γ-carboline ring systems were elaborated.^37^ In addition, novel approaches from both traditional and modern chemistry continue to emerge.^40–44^ However, it is still a challenge to offer novel synthetic methods that provide functionalized γ-carbolines from readily available precursors.

We put our efforts into the redirection of the process towards γ-carboline 2a as the major product. To this end, the reaction was conducted under different conditions by varying the solvents, temperature, and the nature and amount of bases. When sodium ethylate in DMSO was used in the reaction with 1a, 2a was isolated in a low yield, along with many decomposition products. Only the starting 1a was recovered when KF in *tert*-BuOH or DMF was used. However, our experiments showed that raising the temperature (and replacing MeOH or EtOH by *tert*-BuOH with higher bp) could improve the yield of the target 2a. Thus, we managed to obtain 2a in 83% isolated yield (42% after recrystallization) directly from 1a in the one-pot procedure involving reflux of wet *tert*-BuOH solution of 1a in the presence of a threefold excess of KOH for 24 h followed by acidification. The applicability of this synthetic procedure was illustrated with hemiindigos 1b–i as examples. The purified white crystalline γ-carbolines 2b–i were obtained from 1b–i in 26–80% yields; compound 2h being the only exception (Table 1).

**Table 1:** Synthesis of γ-carbolines 2

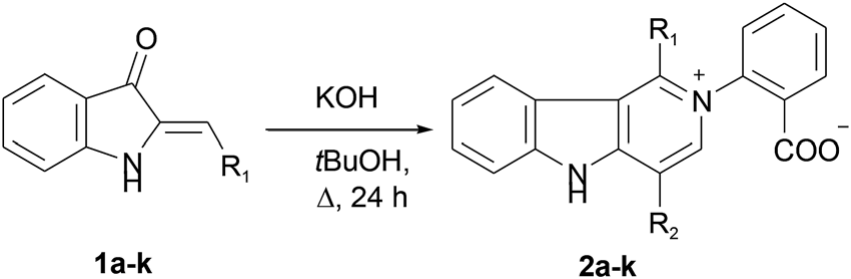				
Entry	Product	R^1^	R^2^	Yield^a^ (%)
1	2a	Ph	Ph	42
2	2b	4-Cl-Ph	4-Cl-Ph	31
3	2c	4-F-Ph	4-F-Ph	35
4	2d	4-Me-Ph	4-Me-Ph	39
5	2e	3-Me-Ph	3-Me-Ph	57
6	2f	4-CF_3_-Ph	4-CF_3_-Ph	28
7	2g	2,4-Cl_2_-Ph	2,4-Cl_2_-Ph	26
8	2h	3-OMe-Ph	3-OMe-Ph	17
9	2i	3-Pyridyl	3-Pyridyl	80
10	2j	Ph	4-F-Ph	–b
11	2k	4-F-Ph	Ph	–b

a The purified yield.

b The crude reaction mixture.

**Scheme 2:**
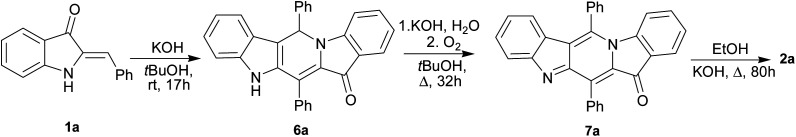
Synthesis of pentacycles 6a, 7a and carbolines 2a.

The reaction can also be used to synthesize γ-carbolines with various substituents at positions 1 and 4 of the ring system. This can be achieved by a crossover protocol that employs a 1 : 1 mixture of unsubstituted compound 1a and 4-fluoro-substituted compound 1c reacting under the above conditions. Two cross-linked carbolines 2j and 2k together with 2a and 2c were identified in the crude reaction mixture by mass spectrometry and by ^1^H, and ^19^F NMR spectroscopy. To isolate possible precursors of 2a, it seemed appropriate to perform a similar reaction of 1a under milder conditions. The process was carried out in alkaline *tert*-BuOH for 17 h at room temperature to afford the first-formed purple fully fused pentacyclic dimer 6a in 83% yield (Scheme 2).

Compound 6a was found amenable to conversion into 2a in 63% yield. The reaction involving heating in alkaline EtOH was disappointingly slow, and a too long time was required to achieve good product yields (Scheme 2). We then found that pentacycle 6a can be converted into the reddish oxidized pentacyclic compound 7a in 81% yield by reflux in alkaline EtOH for 32 h (Scheme 2). Compounds 6c and 7c were obtained from 1c by the similar fashion. However, the reactions do not proceed under argon atmosphere. Under reflux in alkaline MeOH, compound 7a slowly converted into the target carboline 2a in 53% yield (Scheme 2). The poor solubility of compounds 6a and 7a in *tert*-BuOH did not allow us to increase the temperature and thus enhance the efficiency of their transformation into 2a. The structure of 7a was established by single-crystal X-ray diffraction (see ESI†). The structure of 6 was assigned by elemental analysis, mass spectrometry, and 1D and 2D NMR (COSY, HMBC, HSQC and NOESY). Compounds 6 and 7 were found to have the same ring junction in the fused pentacyclic ring system, and the NMR spectrum of 7 displayed only aromatic signals. On the contrary, the spectrum of 6a showed both the NH-1 and aliphatic CH-20 signals at *δ* 10.90 and 7.00 ppm, respectively. The large benzene ring current effects and the presence of the C20–N5 bond account for such downfield shift of the CH-20 proton signal. The chemical shift (*δ*(C) = 56.5 ppm) corresponds to the aliphatic carbon at C-20. Significant nuclear Overhauser effects were observed between the NH signal at *δ* 10.90 ppm and the doublet aromatic signals at 7.62 (H-22, H-26) and 7.35 ppm (H-14), respectively. HMBC experiments showed correlations between the H-29 and H-33 protons and the C-20 carbon.

**Scheme 3:**
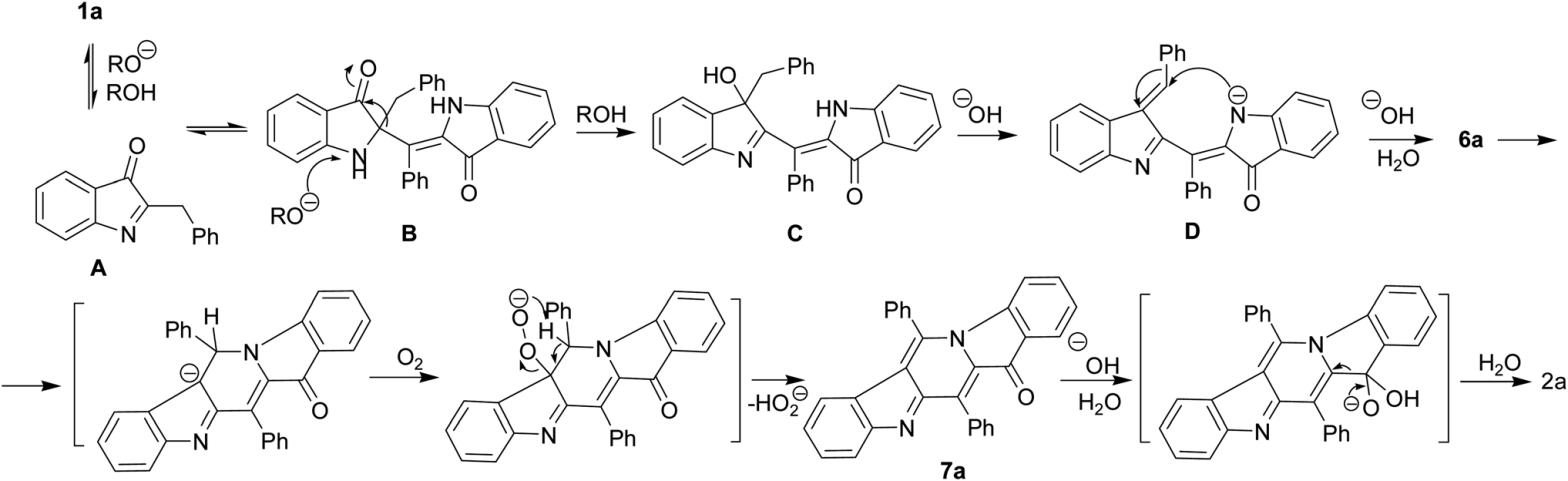
Proposed route for the formation of 6a and 7a.

We believe that certain key steps of the transformation 1a → 2a are now experimentally justified (Scheme 3). Thus, the cascade reaction of bicyclic hemiindigos on the route to tricyclic γ-carboline system includes information on at least two its pentacyclic precursors 6 and 7. The presumable route to these intermediates is depicted in Scheme 3 with 1a as an example. Alkaline-promoted tautomerization of nucleophilic 1a to electrophilic 2-benzylindoleninone A allows coupling between them to afford pseudoindoxyl adduct B that undergoes 1,2-shift of the benzyl moiety to directly obtain a rearranged hydroxyindolenine C.

The transformations C → D → 6a likely involve dehydration of C to give intermediate D and a subsequent ring closure yielding pentacycle 6a. The air oxidation of 6a into 7a occurs at the next step, probably, *via* base-induced deprotonation of the ring NH. It is the cleavage of the C12–C4 bond in compound 7 that seems to be responsible for the formation of the target 2.

**Scheme 4:**
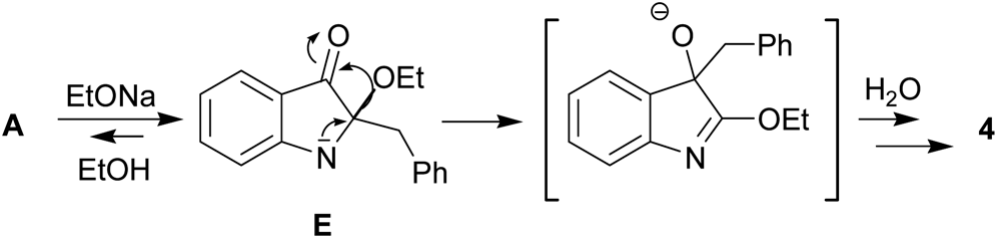
Proposed route for the aza-benzilic type rearrangement of indoleninone A into 4.

The indoleninone/indolenine type intermediates A, C, and D postulated in this work are often found in synthetic and natural indole heterocycles.^45–47^ The simplest 2-unsubstituted indoleninone was postulated to be one of the key intermediates in the Baeyer–Drewson indigo synthesis that proceeds as a multi-step reaction employing acetone and 2-nitrobenzaldehyde in the presence of alkali.^48^ L. Kalb and J. Bayer described 2-phenylindoleninone-3 as a reddish solid that rearranged to 3-hydroxy-3-phenyl-1,3-dihydro-2*H*-indol-2-one in alkaline conditions.^49^ Apparently, indoleninone A participates in the formation of dimer 6 but also of rearranged byproducts, 2-indolinones 4 and 5 (Schemes 1 and 4).

**Scheme 5:**
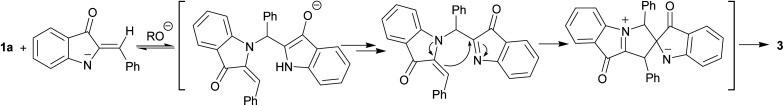
Proposed route for spiroindoxyl 3 formation.

The umpolung reactivity of the exomethylidene C=C bond of 1a seems to result in its tautomerization to highly electrophilic α-imineketone form A. However, 1 as enamines behave like *C*-nucleophiles that couple with A. Easy addition of both *C*-nucleophilic 1a and *O*-nucleophiles RO^−^ to the C=N bond of A leads to the formation of corresponding intermediates B and E that undergo a 1,2-shift of the benzyl moiety. Note that compound 1a in neutral media and its *N*-methyl derivative in alkaline conditions do not furnish corresponding rearrangement products and their methyl analogs. Thus, the anionotropic rearrangement in B can be considered as the driving force of the process, since from this point on the reaction is irreversible. 2,2-Disubstituted indoxyls (indolin-3-ones) with a quaternary center at position 2 are prone to rearrangement that can be induced thermally, under acidic conditions, or under basic conditions.^50–52^ There are also other examples of similar rearrangement of certain 2,2-disubstituted indol-3-one derivatives.^53,54^ In total, all these cascade reactions involve an addition-rearrangement closely related to the aza-benzilic type rearrangement sequence of α-imineketones followed by the formation of pentacycle 6 or byproducts 4, 5 depending on the nature of the *C*- or *O*-nucleophiles (Schemes 4 and 5, respectively). Scheme 4 depicts this mechanism for the case of byproduct 4.

Recently, S. Yamabe showed that the related benzilic type rearrangement of α-diketones involving a carbanion [1,2] migration^55^ included both alkyl and aryl groups as migrating units. Surprisingly, in alkaline conditions, we managed to obtain the target γ-carbolines and to avoid the formation of the rearrangement byproducts 4, 5 as the major products.

Three γ-carbolines 2 and the pentacycles 6, 7 with the core unit of potent antileprotic and antimycobacterial drug clofazimine were tested for possible antimycobacterial activity. Initial serendipitous studies revealed promising *in vitro* antimycobacterial activity of 6, 7 in contrast to a low activity of γ-carbolines 2 against both *Mycobacterium tuberculosis* H37Rv and isoniazid-resistant human isolate CN-40 (Table 2). The conjugated system of 6, 7 resembles the ring system of clofazimine and its rimino-analogs. It is believed that the ring system of both rimino-compounds and 6, 7 is responsible for the unique mechanism of their action.

**Table 2:** Antimycobacterial testing results of polycyclic compounds

Compound	Activity (MIC_99_ [μg ml^−1^]) against	
	*M. tuberculosis* H37Rv	CN-40^b^
2a	>1.48 (ref. 1)^a^	NT
2c	>40^a^	NT
2h	>40^a^	NT
2i	>40^a^	NT
6a	1.90–2.53	3.38–4.50
6c	>40^a^	NT
7c	>1.48 (ref. 1)^a^	NT
Isoniazid (INH)	0.05	20
Clofazimine	0.8,^56^ 0.25 (ref. 57)	

a Precipitation upon testing.

b INH resistant; NT – not tested.

Another hemiindigo cascade reaction leading to spiroproduct 3 likely involves a different-type coupling of 1a. The nucleophilic 1,4-addition of the NH-deprotonated form of 1a to another unprotonated molecule occurs by umpolung base-induced catalyzed coupling to afford bis-adduct. Possible intermediates of the process are shown in Scheme 5.

## Experimental section

### Synthesis of γ-carbolines (2a–i)

To a solution of the corresponding 2-arylidene-1*H*-indol-3(2*H*)-ones 1 (0.6 mmol) in *tert*-BuOH (1.6 mL) was added KOH (130 mg, 2.0 mmol), and the reaction mixture was refluxed for 24 h. After cooling of the solution to room temperature, the solvent was evaporated *in vacuo*. Water (2–5 mL) was added to a precipitate and the mixture was acidified with 5% hydrochloric acid to pH 5.0–6.0. The precipitate of crude 2 was filtered off, dried on air and recrystallized from MeOH or MeOH/AcOH.

#### 2-(1,4-Diphenyl-5*H*-pyrido[4,3-*b*]indol-2-ium-2-yl)benzoate (2a)

Compound 2a was synthesized from (*Z*)-2-benzylidene-1,2-dihydro-3*H*-indol-3-one 1a (130 mg, 0.6 mmol) to give a white powder (54 mg, 42% yield): mp 355–357 °C (MeOH); ^1^H NMR (400 MHz, DMSO-d_6_) *δ* 6.61 (d, *J* = 8.2 Hz, 1H), 7.15 (t, *J* = 7.6 Hz, 1H), 7.27 (t, *J* = 7.6 Hz, 1H), 7.33–7.59 (m, 11H), 7.67 (d, *J* = 7.0 Hz, 1H), 7.75 (br s, 2H), 7.81 (d, *J* = 6.8 Hz, 1H), 7.88 (d, *J* = 8.2 Hz, 1H), 8.56 (s, 1H); ^13^C NMR (101 MHz, CD_3_COOD) *δ* 166.45, 164.07, 147.41, 143.69, 140.78, 139.22, 136.64, 131.52, 130.58, 130.10, 129.57, 129.40, 129.08, 128.50, 128.18, 128.02, 127.60, 127.50, 126.77, 123.60, 121.71, 121.00, 120.61, 119.95, 118.95, 112.20; IR (KBr, cm^−1^) 3059, 1610, 1595, 1587, 1560, 1489, 1474, 1445, 1424, 1342, 1276, 1230, 1120, 793, 759, 700; MS, *m*/*z* [M]^+^: 440; anal. calcd for C_30_H_20_N_2_O_2_: C, 81.80; H, 4.58; N, 6.36. Found: C, 81.67; H, 4.73; N, 6.24.

### Synthesis of pentacycles 6a, 7a

#### 5,12-Diphenyl-5,13-dihydro-11*H*-pyrido[1,2-*a*:4,5-*b*′]diindol-11-one (6a)

To a solution of 1a (260 mg, 1.18 mmol) in *tert*-BuOH (3.2 mL) was added potassium hydroxide (260 mg, 4.02 mmol) and the mixture was stirred at room temperature for 17 h. Water (3–5 mL) was added to a precipitate, the aqueous layer was extracted with benzene (3 × 20 mL) or ethyl acetate (2 × 50 mL), the organic layers were combined and concentrated *in vacuo*. The residue was purified by flash column chromatography on silica gel (C_6_H_6_/MeOH, 5/1, v/v) to give 6a (177 mg, 83% yield) as a blue powder: mp 207–209 °C (C_6_H_6_/MeOH). ^1^H NMR (600 MHz, DMSO-d_6_) *δ* 6.88 (t, *J* = 7.4 Hz, 1H), 6.98 (t, *J* = 7.5 Hz, 1H), 7.02 (s, 1H), 7.08–7.58 (m, 12H), 7.61 (d, *J* = 7.0 Hz, 2H), 7.64 (d, *J* = 7.6 Hz, 2H), 10.89 (s, 1H); ^13^C NMR (151 MHz, DMSO-d_6_) *δ* 182.29, 150.70, 143.05, 139.51, 135.70, 131.86, 130.52, 130.42, 130.17, 129.32, 128.98, 128.87, 128.34, 127.07, 124.82, 124.19, 123.84, 123.19, 120.61, 120.10, 119.29, 118.55, 113.07, 112.90, 111.39, 56.66; IR (KBr, cm^−1^) 3431, 3281, 2921, 2851, 1651, 1614, 1584, 1566, 1511, 1474, 1435, 1346, 1326, 1295, 1270, 1239, 1189, 1153, 1134, 1101, 1063, 980, 744, 721, 560, 475, 429, 403; MS, *m*/*z* [M]^+^: 424; anal. calcd for C_30_H_20_N_2_O: C, 84.88; H, 4.75; N, 6.60. Found: C, 84.74; H, 4.98; N, 6.43.

#### 5,12-Diphenyl-11*H*-pyrido[1,2-*a*:4,5-*b*′]diindol-11-one (7a)

To a solution of 6a (84 mg, 0.198 mmol) in ethanol (10 mL) under reflux was added potassium hydroxide (150 mg, 2.3 mmol) and the mixture was refluxed for 32 h. Reaction mixture was acidified with hydrochloric acid to pH 2.0–3.0. The solid precipitate was filtered off. The organic layer was diluted with water (10–20 mL) and was added 86% potassium hydroxide to pH 8.0–10.0. The precipitate was filtered off, washed with water, dried on air. The residue was purified by flash column chromatography on silica gel (C_6_H_6_/MeOH, 5/1, v/v) 7a (68 mg, 81% yield) as a red powder: mp 317–319 °C. ^1^H NMR (400 MHz, DMSO-d_6_) *δ* 6.02 (d, *J* = 8.6 Hz, 1H), 6.56 (d, *J* = 7.6 Hz, 1H), 6.98 (t, *J* = 7.1 Hz, 1H), 7.31 (t, *J* = 7.2 Hz, 1H), 7.35–7.96 (m, 14H); ^13^C NMR (101 MHz, DMSO-d_6_) *δ* 182.62, 162.80, 158.07, 154.83, 147.72, 141.90, 136.37, 132.37, 131.84, 131.81, 131.14, 130.68, 129.44, 129.14, 129.01, 128.04, 127.92, 127.41, 126.82, 126.64, 125.16, 122.79, 122.22, 121.54, 120.66, 116.24; IR (KBr, cm^−1^) 3050, 1703, 1608, 1582, 1541, 1466, 1443, 1362, 1346, 1297, 1221, 1209, 1094, 996, 769, 755, 737, 697; MS, *m*/*z* [M]^+^: 422; anal. calcd for C_30_H_18_N_2_O: C, 85.29; H, 4.29; N, 6.63. Found: C, 85.37; H, 4.33; N, 6.54.

See ESI† for more details and spectral data.

## Conclusions

In summary, we developed a novel uninterrupted cascade reaction to produce 1,4-diaryl-disubstituted dipolar γ-carbolines 2 with a carboxylate group and their two indole fused pentacyclic precursors 6, 7 from hemiindigos 1. The driving force of the reactions is likely to be the addition-rearrangement process identified based on the availability of precursor 6 and byproducts 4, 5 that were isolated for the first time. The multiform reactivity of pro-electrophilic hemiindigos 1 was revealed, including umpolung C=C bond reactivity by its tautomerization to the indoleninone form A that provides the novel aza-benzilic type rearrangement sequence induced by *C*-nucleophiles. Thus, we found a prized antimycobacterial highly conjugated rimino-type scaffold that can be used to design clofazimine-like rimino-compounds. Pro-electrophilic hemiindigos including indirubin seem to be promising for the new strategy for utilizing pro-electrophilic compounds as agents for the treatment of certain neurological diseases including relapsing multiple sclerosis.^58^ The chemistry described in this work is new to the hemiindigos. The γ-carboline and novel indole-fused pentacyclic molecular scaffolds may open a new way in the search of pharmacologically important compounds. This gives an impetus to future research aimed to optimize synthetic routes to these species.

## Conflicts of interest

There are no conflicts to declare.

## Supplementary Material

RA-009-C9RA07807J-s001

RA-009-C9RA07807J-s002

## References

[cit1] Gaboriaud-Kolar N. , Nam S. and Skaltsounis A. L., A Colorful History: The Evolution of Indigoids, in Progress in the Chemistry of Organic Natural Products, ed. A. Kinghorn, H. Falk and J. Kobayashi, Springer, Cham, 2014, vol. 99 10.1007/978-3-319-04900-7_2 25296438

[cit2] Shakoori A., Bremner J. B., Willis A. C., Haritakun R., Keller P. A. (2013). J. Org. Chem..

[cit3] Shakoori A., Bremner J. B., Abdel-Hamid M. K., Willis A. C., Haritakun R., Keller P. A. (2015). Beilstein J. Org. Chem..

[cit4] Sele A. M., Bremner J. B., Willis A. C., Haritakun R., Griffith R., Keller P. A. (2015). Tetrahedron.

[cit5] Butler N. M., Hendra R., Bremner J. B., Willis A. C., Lucantoni L., Avery V. M., Keller P. A. (2018). Org. Biomol. Chem..

[cit6] Petermayer C., Dube H. (2018). Acc. Chem. Res..

[cit7] Pianowski Z. L. (2019). Chemistry.

[cit8] Petermayer C., Thumser S., Kink F., Mayer P., Dube H. (2017). J. Am. Chem. Soc..

[cit9] Berdnikova D. V. (2019). Chem. Commun..

[cit10] Baeyer A. (1883). Chem. Ber..

[cit11] Arai T., Ikegami M. (1999). Chem. Lett..

[cit12] Chen W. S., Li B., Zhang W. D., Yang G. J., Qiao C. Z. (2001). Chin. Chem. Lett..

[cit13] Leclerc S., Garnier M., Hoessel R., Marko D., Bibb J. A., Snyder G. L., Greengard P., Biernati J., Wui Y.-Z., Mandelkowi E.-M., Eisenbrand G., Meijer L. (2001). J. Biol. Chem..

[cit14] Cheng X., Merz K.-H., Vatter S., Zeller J., Muehlbeyer S., Thommet A., Christ J., Wölfl S., Eisenbrand G. (2017). J. Med. Chem..

[cit15] Velezheva V. S., Melman A. I., Polshakov V. I., Anisimova O. S. (1992). Chem. Heterocycl. Compd..

[cit16] Velezheva V. S., Marshakov V. Y., Melman A. I., Kurkovskaya L. N., Suvorov N. N. (1988). Zh. Org. Khim..

[cit17] Velezheva V. S., Marshakov V. Y. (1992). Chem. Heterocycl. Compd..

[cit18] Velezheva V. S., Brennan P. J., Marshakov V. Y., Gusev D. V., Lisichkina I. N., Peregudov A. S., Tchernousova L. N., Smirnova T. G., Andreevskaya S. N., Medvedev A. E. (2004). J. Med. Chem..

[cit19] Wang F., Li Z., Wang J., Li X., Cheng J.-P. (2015). J. Org. Chem..

[cit20] Yang L. C., Tan Z. Y., Rong Z. Q., Liu R., Wang Y. N., Zhao Y. (2018). Angew. Chem., Int. Ed. Engl..

[cit21] Zhou J., Wang B., He X.-H., Liu L., Wu J., Lu J., Peng C., Rao C.-L., Han B. (2019). J. Org. Chem..

[cit22] Guo C., Schedler M., Daniliuc C. G., Glorius F. (2014). Angew. Chem., Int. Ed..

[cit23] Stahl R., Galli R., Güller R., Borschberg H.-J. (1994). Helv. Chim. Acta.

[cit24] Davion Y., Joseph B., Merour J.-Y. (1998). Synlett.

[cit25] Yang L., Huang W., He X.-H., Yang M.-C., Li X., He G., Peng C., Han B. (2016). Adv. Synth. Catal..

[cit26] Velezheva V. S., Nevskii K. V., Suvorov N. N. (1985). Chem. Heterocycl. Compd..

[cit27] Hooper M., Pitkethly W. N. (1972). J. Chem. Soc., Perkin Trans. 1.

[cit28] Babii O. L., Hodak A. A., Peregudov A. S., Polshakov V. I., Starikova Z. A., Borisov Y. A., Fedorov Y. V., Velezheva V. S. (2017). Russ. Chem. Bull..

[cit29] Zhang M., Li T., Qian M., Li K., Qin Y., Zhao T., Yang L.-Q. (2018). J. Heterocycl. Chem..

[cit30] Carrasco M. P., Machado M., Gonsalves L., Sharma M., Gut J., Lukens A. K., Wirth D. F., Andre V., Duarte M. T., Guedes R. C., dos Santos D. J. V. A., Rosenthal P. J., Mazitschek R., Prudencio M., Moreira R. (2016). ChemMedChem.

[cit31] Klein L. L., Petukhova V., Wan B., Wang Y., Santasiero B. D., Lankin D. C., Pauli G. F., Franzblau S. G. (2014). Bioorg. Med. Chem. Lett..

[cit32] Peregudov A. S., Godovikov I. A., Fedorova I. N., Istomina O. V., Lyssenko K. A., Velezheva V. S. (2013). Russ. Chem. Bull..

[cit33] Velezheva V. S., Fedorova I. N., Babii O. L., Anisimov A. A., Bushmarinov I. S., Peregudov A. S. (2015). Curr. Org. Synth..

[cit34] Miller K. A., Williams R. M. (2009). Chem. Soc. Rev..

[cit35] Gong J. H., Lee K. Y., Son J. S., Kim J. N. (2003). Indium mediated Barbier reaction of ninhydrin and isatin. Bull. Korean Chem. Soc..

[cit36] Miki Y., Kuromatsu M., Miyatake H., Hamamoto H. (2007). Tetrahedron Lett..

[cit37] Dai J., Dan W., Zhang Y., Wang J. (2018). Eur. J. Med. Chem..

[cit38] Alekseyev R. S., Kurkin A. V., Yurovskaya M. A. (2011). Chem. Heterocycl. Compd..

[cit39] Alekseyev R. S., Kurkin A. V., Yurovskaya M. A. (2009). Chem. Heterocycl. Compd..

[cit40] Verma S., Kumar M., Mishra P. K., Verma A. K. (2019). Org. Lett..

[cit41] Uredi D., Motati D. R., Watkins E. B. (2018). Org. Lett..

[cit42] Garg V., Kumar P., Verma A. K. (2018). J. Org. Chem..

[cit43] Wang T.-T., Zhang D., Liao W.-W. (2018). Chem. Commun..

[cit44] Hingane D. G., Parekh N. P., Khan A., Kusurkar R. S. (2016). Synth. Commun..

[cit45] Bu L., Li J., Yin Y., Qiao B., Chai G., Zhao X., Jiang Z. (2018). Chem.–Asian J..

[cit46] Dalpozzo R., Bartoli G., Bencivenni G. (2012). Chem. Soc. Rev..

[cit47] Feldman K. S., Gonzalez I. Y., Glinkerman C. M. (2014). J. Am. Chem. Soc..

[cit48] Baeyer A., Drewson V. (1882). Chem. Ber..

[cit49] Kalb L., Bayer J. (1912). Chem. Ber..

[cit50] Liu Y., McWhorter W. W. (2003). J. Org. Chem..

[cit51] Patrick J. B., Witkop B. (1950). J. Am. Chem. Soc..

[cit52] Song Z.-L., Fan C.-A., Tu Y.-Q. (2011). Chem. Rev..

[cit53] Richman R. J., Hassner A. (1968). J. Org. Chem..

[cit54] Sukari M. A., Vernon J. A. (1983). J. Chem. Soc., Perkin Trans. 1.

[cit55] Yamabe S., Tsuchida N., Yamazaki S. (2006). J. Org. Chem..

[cit56] Barry V. C., Belton J. G., Conalty M. L., Denneny J. M., Edward D. W., O'Sullivan J. F., Twomey D., Winder F. (1957). Nature.

[cit57] Xu J., Wang B., Fu L., Zhu H., Guo S., Huang H., Yin D., Zhang Y., Lu Y. (2019). Antimicrob. Agents Chemother..

[cit58] Satoh T., Rezaie T., Seki M., Sunico C. R., Tabuchi T., Kitagawa T., Yanagitai M., Senzaki M., Kosegawa C., Taira H., McKercher S. R., Hoffman J. K., Roth G. P., Lipton S. A. (2011). J. Neurochem..

