# Supplementary material for: Novel base-initiated cascade reactions of hemiindigos to produce dipolar γ-carbolines and indole-fused pentacycles
Source: RSC Adv. 2019 Dec 13;9(71):41402–8. doi: 10.1039/c9ra07807j (PMC9076487; doi:10.1039/c9ra07807j)
Supplement: RA-009-C9RA07807J-s001 [file RA-009-C9RA07807J-s001.pdf]

Electronic Supplementary Material (ESI) for RSC Advances.
This journal is © The Royal Society of Chemistry 2019

SUPPORTING INFORMATION

Novel base-initiated cascade reactions of hemiindigos to produce dipolar γ-Carbolines and indole-fused pentacycles

V. S. Velezheva,[a]* O. L. Babii,[a] A. A. Hodak,[a] E. A. Alekseeva,[a] Yu. V. Neliubina,[a] I. A. Godovikov,[a] A. S. Peregudov,[a] K. B. Majorov,[b] B. V. Nikonenko[b]

[a]*A. N. Nesmeyanov Institute of Organoelement Compounds, Russian Academy of Sciences, 28 Vavilov Str., 119991 GSP-1 Moscow, Russia*

vel@ineos.ac.ru

[b]*Laboratory for Immunogenetics, Central Institute for Tuberculosis, Moscow, Russia*

Table of Contents:

1. General Information ______ **S2**

2. Experimental procedures and spectral data ______ **S2**

3. Copies of NMR Spectra ______ **S8**

4. X-ray diffraction ______ **S38**

5. References ______ **S40**

1. General Information.

All chemicals and solvents were purchased from commercial sources and were used without further purification. IR spectra were run as KBr disks on a IR-Fourier-spectrometer Magna 750 IR Nicolet. ^{1}H NMR (300, 400 or 600 MHz), ^{13}C NMR (75, 101 or 151 MHz) and ^{19}F NMR (282 MHz) spectra were recorded on a Bruker Avance^{TM} - 300, Bruker Avance^{TM} - 400 or Bruker Avance^{TM} - 600 spectrometers. NMR spectra were recorded in solutions of DMSO-d$_6$, CF_3COOD, CD_3COOD. Chemical shifts were reported in the δ scale relative to residual signal of solvent DMSO (2.50 ppm), CF_3COOH (11.50 ppm) and CH_3COOH (2.04 ppm) for ^{1}H-NMR and to the central line of DMSO-d$_6$ (39.5 ppm), CF_3COOD (116.6, 164.2 ppm), CD_3COOD (20.0, 179.0 ppm) for ^{13}C-NMR, respectively. ^{19}F-NMR spectra were recorded using CD_3COOD, DMSO-d$_6$ as a lock solvent. COSY-HH, NOESY-HH, HMBC-HC and HMQC-HC were used where necessary in assigning NMR spectra. Multiplicities are indicated by s (singlet), d (doublet), t (triplet), and m (multiplet). Coupling constants *J* are reported in Hertz. Among all chromatographically homogenous γ-carbolines **2**, 3-aryl and 2,3-diaryl substituted carbolines **2e** and **2g-i** featured the ^{1}H and ^{13}C NMR spectra with the increased number of aromatic, or aromatic and aliphatic proton, and carbon signals, probably, due to the hindered rotation of the C1-aryl ring around the Ar-C1 bond. Mass spectra were recorded on a Finnigan LCQ Advantage for ESI, on a Finnigan LTQ FT Ultra for HRMS (ICR) and on a Finnigan Polaris Q for EI. Elemental analyses were performed at the laboratory of microanalysis of A. N. Nesmeyanov Institute of Organoelement Compounds, Moscow. Melting points were uncorrected. X-Ray diffraction experiments for compounds **2a**, **3**, **4**, and **7** were carried out with a Bruker SMART APEX 1000 CCD area detector, using graphite monochromated Mo-Kα radiation (lλ = 0.71073 Å, ω-scans) at temperature 100 °K. Flash Column chromatography was performed on Silica gel Merck 60 (Merck, 230 mesh). Starting materials 2-arylidene-1*H*-indol-3(2*H*)-ones (indogenides) **1** were prepared by literature procedures.[1-3]

Biological evaluation.A series of compounds were tested for their in vitro antimycobacterial activity against a laboratory strain M. tuberculosis H37Rv and against a clinical isolate of INH-resistant *M. tuberculosis* with selective single INH resistance, designated as CN-40. M. tuberculosis strain H37Rv (museum strain) was originally obtained from the Institute Pasteur, Paris, France (a kind gift of G. Marchal). The CN-40 strain was isolated from a TB patient at the TB Research Institute of the Russian Academy of Medical Sciences. MICs against M. tuberculosis were determined by standard microdilution in microtubes using Dubos medium containing 0.05% Tween 80.

M. tuberculosis strains. *M. tuberculosis* strain H37Rv (Pasteur) was maintained and prepared for in vitro infection exactly as described previously.[7,8] Briefly, following 3 weeks of growth on Loewenstein-Jensen medium at 37°C, mycobacteria were suspended in sterile saline containing 0.05% Tween 20 and 0.1% bovine serum albumin (BSA; Sigma), washed, aliquoted (10 mg of semidry bacterial mass in 1 ml), and stored at −80°C. To obtain the log-phase bacteria for challenge, 50 µl from a thawed aliquot was added to 5 ml of Dubos broth (Difco, Detroit, Mich.) supplemented with 0.5% BSA and incubated for 1 week at 37°C. The mycobacterial suspension (0.5 ml) was diluted in 20 ml of fresh warm Dubos-BSA medium and further cultured for 1 week. The resulting suspension was washed three times at 3,000 × g and 4°C with 0.02% EDTA-phosphate-buffered saline (PBS) (Ca2+- and Mg2+-free) solution, resuspended in medium 2, and filtered through a 4-µm-pore-size filter (Sigma) to remove clumps. To estimate the CFU content in the filtrate, 10 µl from each fivefold serial dilution was plated onto Dubos agar (Difco), and the total number of microcolonies in the spot was calculated under an inverted CK-2 microscope (Olympus, Osaka, Japan) after being cultured for 3 days at 37°C. The bulk of the filtered culture was stored at 4°C, and it was found that no change in the CFU content occurred during this period.

The lowest concentration of a compound resulting in no visible growth of *M. tuberculosis* for 2 weeks was considered the MIC. All samples were tested twice in triplicate. We also used a micro method developed in our laboratory based on measurement under a microscope of the volume of growing compact mycobacterial culture in the wells of round-bottom 96-well plates in the presence or absence of a tested compound (K. Majorov, unpublished data).

2. Experimental procedures and spectral data.

General Methods for the Synthesis of γ-Carbolines (2a-i).

Method A: To a solution of the corresponding 2-arylidene-1*H*-indol-3(2*H*)-ones **1** (0.6 mmol) in *tert*-BuOH (1.6 mL) was added KOH (130 mg, 2.0 mmol), and the reaction mixture was refluxed for 24 h. After cooling of the solution to room temperature, the solvent was evaporated in vacuo. Water (2-5 mL) was added to a precipitate and the mixture was acidified with 5% hydrochloric acid to pH 5.0-6.0. The precipitate of cude **2** was filtered off, dried on air and recrystallized from MeOH or MeOH/AcOH.

Method B: To a solution of 2-benzylidene-1,2-dihydro-3*H*-indol-3-one **1a** (0.6 mmol) in DMSO (2 mL) was added EtONa (2 mmol) and the reaction mixture was heated to 50 °C for 6 h. Water (2 mL) was added, the precipitate formed was filtered off, washed with water and dried on air. Compound **2a** was obtained as a white powder (5 % yield): m.p. 355-357 °C (MeOH).

Method C: To a suspension of the corresponding 2-arylidene-1H-indol-3(2H)-ones **1e**, **1f** and **1i** (0.6 mmol) in *tert*-BuOH (7 mL), was added potassium hydroxide (388 mg, 6.0 mmol) and the mixture was refluxed. Work-up of the reaction mixture was performed as described above.

Method B: To a solution of 5,12-diphenyl-11*H*-pyrido[1,2-a:4,5-b']diindol-11-one **7** (20 mg, 0.047 mmol) in methanol (9 mL) under reflux was added potassium hydroxide (929 mg, 14.27 mmol) and the mixture was refluxed for 80 h. After evaporation of methanol from a reaction mixture, to a residue was added water (3-5 mL) and the mixture was acidified with 5% hydrochloric acid to pH 5.0-6.0. The precipitate formed was filtered off, washed with water and dried on air. Compound **2a** was obtained as a white powder (11 mg, 53% yield): m.p. 355-357 °C (MeOH).

Method E: To a solution of 5,12-diphenyl-5,13-dihydro-11*H*-pyrido[1,2-a:4,5-b']diindol-11-one **6** (60 mg, 0.142 mmol) in ethanol (6 mL) potassium hydroxide (107 mg, 1.64 mmol) was added and the mixture was refluxed for 135 h. After evaporation of ethanol from a reaction mixture, to a residue was added water (3-5 mL) and the mixture was acidified with 5% hydrochloric acid to pH 5.0-6.0. The precipitate formed was filtered off, washed with water and dried on air. Compound **2a** was obtained as a white powder (30 mg, 48% yield): m.p. 355-357 °C (MeOH).

2-(1,4-Diphenyl-5*H*-pyrido[4,3-b]indol-2-ium-2-yl)benzoate (2a).

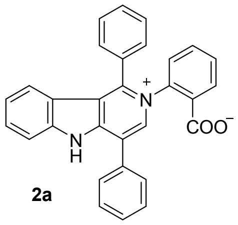

2a

Compound **2a** was synthesized from (Z)-2-benzylidene-1,2-dihydro-3H-indol-3-one **1a** (130 mg, 0.6 mmol) according to the method A to give a white powder (54 mg, 42% yield): m.p. 355-357 °C (MeOH); ^{1}H NMR (400 MHz, DMSO-d$_6$) δ 6.61 (d, *J* = 8.2 Hz, 1H), 7.15 (t, *J* = 7.6 Hz, 1H), 7.27 (t, *J* = 7.6 Hz, 1H), 7.33-7.59 (m, 11H), 7.67 (d, *J* = 7.0 Hz, 1H), 7.75 (br s, 2H), 7.81 (d, *J* = 6.8 Hz, 1H), 7.88 (d, *J* = 8.2 Hz, 1H), 8.56 (s, 1H); ^{13}C NMR (101 MHz, CD$_3$COOD) δ 166.45, 164.07, 147.41, 143.69, 140.78, 139.22, 136.64, 131.52, 130.58, 130.10, 129.57, 129.40, 129.08, 128.50, 128.18, 128.02, 127.60, 127.50, 126. 77, 123.60, 121.71, 121.00, 120.61, 119.95, 118.95, 112.20; IR (KBr, cm^{-1}) 3059, 1610, 1595, 1587, 1560, 1489, 1474, 1445, 1424, 1342, 1276, 1230, 1120, 793, 759, 700; MS, m/z [M]$^+$: 440; Anal. Calcd. for $C_{30}H_{20}N_2O_2$: C, 81.80; H, 4.58; N, 6.36. Found: C, 81.67; H, 4.73; N, 6.24.

2-[1,4-Bis(4-chlorophenyl)-5*H*-pyrido[4,3-b]indol-2-ium-2-yl]benzoate (2b).

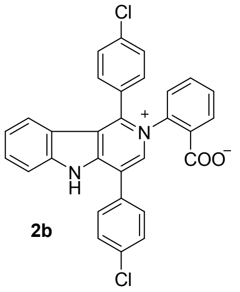

2b

Compound **2b** was synthesized from (Z)-2-(4-chlorobenzylidene)-1,2-dihydro-3H-indol-3-one **1b** (150 mg, 0.6 mmol) according to the method A to give a white powder (46 mg, 31% yield): m.p. 351-353 °C (MeOH); ^{1}H NMR (600 MHz, CF$_3$COOD) δ 6.57 (d, *J* = 8.2 Hz, 1H), 6.81 (t, *J* = 7.6 Hz, 1H), 6.91 (t, *J* = 9.0 Hz, 2H), 7.03 (t, *J* = 7.6 Hz, 2H), 7.14-7.24 (m, 8H), 7.34 (t, *J* = 7.1 Hz, 1H), 7.81 (d, *J* = 6.8 Hz, 1H), 7.93 (s, 1H); ^{13}C NMR (151 MHz, CF$_3$COOD) δ 168.66, 147.58, 144.64, 144.49, 140.99, 140.85, 140.33, 138.07, 137.01, 136.14, 134.74, 132.67, 131.00, 129.94, 129.86, 129.53, 129.23, 129.15, 128.96, 128.85, 128.74, 128.04, 127.06, 124.28, 123.45, 121.75, 121.46, 120.34, 120.03, 112.16; IR (KBr, cm^{-1}) 3397, 3064, 2954, 2604, 1610, 1598, 1557, 1491, 1476, 1455, 1431, 1397, 1346, 1231, 1093, 1016, 1007, 848, 826, 760, 743, 731; MS, m/z [M]$^+$: 509; Anal. Calcd. for $C_{30}H_{18}Cl_2N_2O_2$: C, 70.74; H, 3.56; N, 5.50; Cl, 13.92. Found: C, 70.70; H, 3.56; N, 5.42; Cl, 13.77.

2-[1,4-Bis(4-fluorophenyl)-5*H*-pyrido[4,3-b]indol-2-ium-2-yl]benzoate (2c).

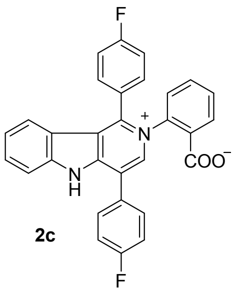

2c

Compound **2c** was synthesized from (Z)-2-(4-fluorobenzylidene)-1,2-dihydro-3H-indol-3-one **1c** (141 mg, 0.6 mmol) according to the method A to give a white powder (49 mg, 35% yield): m.p. 337-339 °C (MeOH); ^{1}H NMR (600 MHz, CD$_3$COOD) δ 6.98 (d, *J* = 7.7 Hz, 1H), 7.28-7.88 (m, 14H), 8.12 (d, *J* = 6.3 Hz, 1H), 8.77 (s, 1H); ^{13}C NMR (151 MHz, CF$_3$COOD) δ 168.63, 164.06 (d, *J* = 153 Hz), 163.85 (d, *J* = 153 Hz), 147.73, 144.66, 140.89, 140.48, 136.23, 134.76, 132.66, 130.96, 130.10, 130.05, 129.90, 129.68, 129.62, 128.92, 125.85, 125.83, 124.92, 124.89, 124.35, 123.42, 121.91, 121.48, 120.48, 120.20, 112.21; ^{19}F NMR (300 MHz, CD$_3$COOD) δ -30.92 (s, 1F), -33.83 (s, 1F); IR (KBr, cm^{-1}) 3435, 3059, 2920, 2851, 1606, 1562, 1501, 1476, 1455, 1431, 1404, 1351, 1305, 1278, 1229, 1160, 1121, 1098, 1017, 838, 776, 760, 671, 531; MS, m/z [M]$^+$: 476; Anal. Calcd. for $C_{30}H_{18}F_2N_2O_2$: C, 75.62; H, 3.81; N, 5.88; F, 7.97. Found: C, 75.26; H, 3.68; N, 5.85; F, 7.61.

2-[1,4-Bis(4-methylphenyl)-5*H*-pyrido[4,3-b]indol-2-ium-2-yl]benzoate (2d).

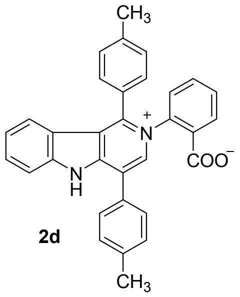

2d

Compound **2d** was synthesized from (Z)-2-(4-methylbenzylidene)-1,2-dihydro-3H-indol-3-one **1d** (138 mg, 0.6 mmol) according to the method A to give a white powder (53 mg, 39% yield): m.p. 335-337 °C (MeOH); ^{1}H NMR (600 MHz, CD_3COOD) δ 2.42 (s, 3H), 2.46 (s, 3H), 6.95 (d, *J* = 7.6 Hz, 1H), 7.23-7.81 (m, 14H), 8.12 (d, *J* = 6.9 Hz, 1H), 8.68 (s, 1H); ^{13}C NMR (151 MHz, CD_3COOD) δ 167.99, 148.93, 144.92, 141.93, 141.37, 140.67, 140.08, 137.87, 132.62, 131.84, 130.61, 130.11, 129.98, 129.53, 129.47, 129.22, 128.89, 128.82, 128.54, 127.60, 122.93, 122.29, 122.05, 121.41, 120.23, 113.44, 20.44, 20.33; IR (KBr, cm-1) 3428, 3063, 3028, 2918, 2859, 1617, 1598, 1588, 1561, 1500, 1476, 1454, 1428, 1344, 1313, 1276, 1257, 1232, 1219, 1120, 1022, 881, 843, 821, 758, 671, 617, 522; MS, m/z [M]+: 468; Anal. Calcd. for $C_{32}H_{24}N_2O_2$: C, 82.03; H, 5.16; N, 5.98. Found: C, 81.75; H, 5.39; N, 5.78.

2-[1,4-Bis(3-methylphenyl)-5*H*-pyrido[4,3-b]indol-2-ium-2-yl]benzoate (2e).

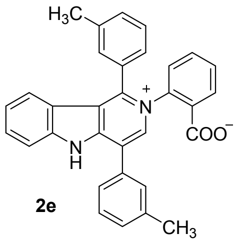

2e

Compound **2e** was synthesized from (Z)-2-(3-methylbenzylidene)-1,2-dihydro-3H-indol-3-one **1e** (138 mg, 0.6 mmol) according to the method A to give a white powder (68 mg, 49% yield): m.p. 327-329 °C (MeOH); ^{1}H NMR (600 MHz, CF_3COOD) δ 1.81 (s, 3H), 1.95 (s, 3H), 6.53 (d, *J* = 6.4 Hz, 1H), 6.64-7.29 (m, 14H), 7.72 (br s, 1H), 7.87 (s, 1H); ^{13}C NMR (151 MHz, CF_3COOD) δ 168.88, 168.73, 148.79, 148.73, 144.56, 140.64, 140.62, 140.58, 140.50, 140.08, 139.63, 139.61, 135.87, 135.80, 134.47, 134.30, 132.37, 132.16, 131.32, 131.31, 130.59, 130.57, 130.54, 129.77, 129.45, 128.97, 128.87, 128.78, 128.73, 128.66, 128.59, 127.99, 127.83, 125.26, 124.56, 124.45, 124.31, 123.03, 122.76, 122.75, 121.67, 121.63, 120.34, 119.94, 111.85, 18.90, 18.69, 18.65; IR (KBr, cm^{-1}) 3393, 3058, 2921, 2852, 1608, 1592, 1563, 1528, 1476, 1454, 1432, 1342, 1287, 1258, 1240, 1223, 1118, 1043, 877, 830, 794, 757, 721, 705, 680, 651, 623; MS, m/z [M]$^+$: 468; Anal. Calcd. for $C_{32}H_{24}N_2O_2$: C, 82.03; H, 5.16; N, 5.98. Found: C, 81.85; H, 5.20; N, 6.07.

Compound **2e** was obtained from **1e** (138 mg, 0.6 mmol) by method B and reflux for 40 h to give 79 mg (57%) of a target product.

2-[1,4-Bis[4-(trifluoromethyl)phenyl]-5*H*-pyrido[4,3-b]indol-2-ium-2-yl]benzoate (2f).

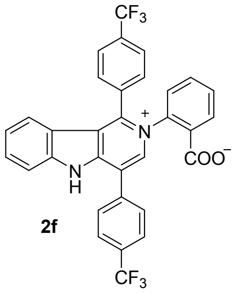

2f

Compound **2f** was synthesized from (Z)-2-[4-(trifluoromethyl)benzylidene]-1,2-dihydro-3H-indol-3-one **1f** (170 mg, 0.6 mmol) according to the method A to give a white powder (31 mg, 18% yield): m.p. 360-362 °C (MeOH); ^{1}H NMR (600 MHz, CF_3COOD) δ 6.41 (d, *J* = 7.9 Hz, 1H), 6.77 (t, *J* = 7.6 Hz, 1H), 7.13-7.44 (m, 13H), 7.78 (d, *J* = 7.7 Hz, 1H), 8.00 (s, 1H); ^{13}C NMR (151 MHz, CF_3COOD) δ 168.57, 147.22, 144.47, 141.00, 140.06, 136.51, 134.80, 133.77 (q, *J* = 33 Hz), 133.26, 132.90 (q, *J* = 33 Hz), 132.73, 132.38, 131.20, 130.20, 129.37, 128.90, 128.32, 128.16, 126.17 (q, *J* = 3 Hz), 125.87 (q, *J* = 4 Hz), 125.78 (q, *J* = 3 Hz), 124.31, 123.64, 122.79 (q, *J* = 272 Hz), 122.42 (q, *J* = 272 Hz), 121.59, 121.55, 121.29, 120.51, 120.46, 119.83, 119.80, 112.35; IR (KBr, cm^{-1}) 3438, 3067, 2918, 2850, 1618, 1600, 1563, 1498, 1476, 1454, 1433, 1407, 1325, 1278, 1231, 1171, 1121, 1110, 1090, 1067, 1020, 845, 707; MS, m/z [M]$^+$: 576; Anal. Calcd. for $C_{32}H_{18}F_6N_2O_2$: C, 66.67; H, 3.15; N, 4.86; F, 19.77. Found: C, 66.53; H, 3.23; N, 4.99; F, 19.53.

Compound **2f** was obtained from **1f** (170 mg, 0.6 mmol) by method B and reflux for 38 h to give 48 mg (28%) of a target product.

2-[1,4-Bis(2,4-dichlorophenyl)-5*H*-pyrido[4,3-b]indol-2-ium-2-yl]benzoate (2g).

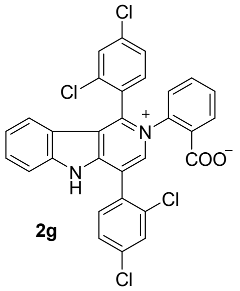

2g

Compound **2g** was synthesized from (Z)-2-(2,4-dichlorobenzylidene)-1,2-dihydro-3H-indol-3-one **1g** (171 mg, 0.6 mmol) according to the method A to give a white powder (45 mg, 26% yield): m.p. 277-279 °C (MeOH); ^{1}H NMR (600 MHz, CF_3COOD) δ 6.61 (d, *J* = 8.2 Hz, 1H), 7.00-7.52 (m, 12H), 8.04 (d, *J* = 7.4 Hz, 1H), 7.97 (s, 1H); ^{13}C NMR (151 MHz, CF_3COOD) δ 160.05, 159.76, 144.88, 144.74, 144.48, 141.06, 140.92, 139.84, 139.15, 137.94, 137.81, 134.80, 133.46, 132.58, 131.38, 131.11, 130.26, 129.84, 129.81, 129.37, 127.86, 127.59, 126.86, 126.62, 126.49, 123.75, 120.91, 119.51, 119.33, 112.22; IR (KBr, cm^{-1}) 3390, 3066, 2928, 2817, 1612, 1585, 1563, 1504, 1471, 1454,

1435, 1381, 1347, 1278, 1227, 1144, 1101, 1041, 949, 887, 862, 812, 797, 768, 710; MS, m/z $[M]^+$: 578; Anal. Calcd. for $C_{30}H_{16}Cl_4N_2O_2$: C, 62.31; H, 2.79; N, 4.84; Cl, 24.52. Found: C, 62.42; H, 2.76; N, 4.73; Cl, 24.34.

2-[1,4-Bis(3-methoxyphenyl)-5*H*-pyrido[4,3-b]indol-2-ium-2-yl]benzoate (2h).

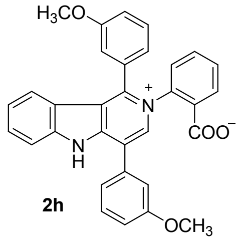

2h

Compound **2h** was synthesized from (Z)-2-(3-methoxybenzylidene)-1,2-dihydro-3H-indol-3-one **1h** (148 mg, 0.6 mmol) according to the method A to give a white powder (27 mg, 17% yield): m.p. 313-315 °C (MeOH); ^{1}H NMR (600 MHz, CF_3COOD) δ 3.36 (d, *J* = 15.3 Hz, 3H), 3.55 (s, 3H), 6.51 (d, *J* = 8.1 Hz, 1H), 6.59-6.70 (m, 3H), 6.73 (t, *J* = 7.6 Hz, 1H), 6.80 (d, *J* = 8.1 Hz, 1H), 6.89 (d, *J* = 7.6 Hz, 1H), 6.96-7.31 (m, 8H), 7.76 (t, *J* = 7.0 Hz, 1H), 7.95 (d, *J* = 2.8 Hz, 1H); ^{13}C NMR (151 MHz, CF_3COOD) δ 168.69, 168.60, 158.61, 158.46, 158.18, 149.62, 149.30, 147.69, 144.26, 140.86, 140.38, 140.31, 136.02, 134.54, 132.49, 132.40, 131.47, 130.96, 130.81, 130.80, 130.49, 130.42, 130.22, 129.78, 128.85, 128.83, 124.37, 124.33, 123.26, 123.24, 121.93, 121.70, 121.57, 121.46, 121.14, 120.19, 120.03, 112.21, 54.97, 54.63, 54.54; IR (KBr, cm^{-1}) 3407, 3066, 2937, 2839, 1609, 1587, 1561, 1536, 1501, 1479, 1464, 1430, 1348, 1307, 1295, 1274, 1254, 1230, 1182, 1120, 1112, 1029, 873, 763, 680; MS, m/z $[M]^+$: 500; Anal. Calcd. for $C_{32}H_{24}N_2O_4$: C, 76.78; H, 4.83; N, 5.60. Found: C, 76.80; H, 4.81; N, 5.55.

2-(1,4-Dipyridin-3-yl-5*H*-pyrido[4,3-b]indol-2-ium-2-yl)benzoate (2i).

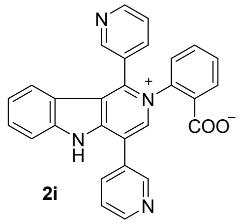

2i

Compound **2i** was synthesized from (Z)-2-(pyridin-3-ylmethylene)-1,2-dihydro-3H-indol-3-one **1i** (131 mg, 0.6 mmol according to the method A to give a white powder (31 mg, 24% yield): m.p. 359-361 °C (MeOH); ^{1}H NMR (300 MHz, DMSO-d_6) δ 6.61 (t, *J* = 7.0 Hz, 1H), 7.22 (t, *J* = 7.6 Hz, 1H), 7.41-8.16 (m, 11H), 8.69 (d, *J* = 3.7 Hz, 1H), 8.73 (d, *J* = 3.5 Hz, 1H), 8.85 (d, *J* = 9.7 Hz, 2H), 8.98 (s, 1H); ^{13}C NMR (151 MHz, CD_3COOD) δ 171.28, 150.32, 148.33, 147.43, 146.59, 145.85, 145.68, 145.17, 145.13, 142.46, 142.35, 140.18, 139.94, 139.55, 139.27, 133.97, 133.41, 132.32, 132.09, 131.47, 131.36, 130.33, 129.99, 129.85, 129.49, 127.80, 126.48, 125.25, 125.05, 123.79, 121.81, 121.77, 121.29, 121.22, 120.73, 120.70, 117.85, 113.96; IR (KBr, cm^{-1}) 3413, 3062, 2919, 2850, 1611, 1596, 1590, 1563, 1533, 1501, 1474, 1453, 1430, 1412, 1352, 1315, 1282, 1234, 1220, 1195, 1123, 1027, 840, 812, 758, 710, 657, 619; MS, m/z $[M]^+$: 442; Anal. Calcd. for $C_{28}H_{18}N_4O_2$: C, 76.01; H, 4.10; N, 12.66. Found: C, 76.19; H, 3.93; N, 12.60.

Compound **2i** was obtained from **1i** (131 mg, 0.6 mmol) by method B and reflux for 30 h to give 104 mg (80%) of a target product.

2-(1,4-Diphenyl-5*H*-pyrido[4,3-b]indol-2-ium-2-yl)benzoate (2a), 9a'-hydroxy-1',3'-diphenyl-1'*H*-spiro[indole-2,2'-pyrrolo[1,2-a]indole]-3,9'(1*H*,9a'*H*)-dione (3), 3-benzyl-3-hydroxy-1,3-dihydro-2*H*-indol-2-one (4), 3-hydroxy-3-[methoxy(phenyl)methyl]-1,3-dihydro-2*H*-indol-2-one (5).

Method A: To a solution of **1a** (1.0 g, 4.5 mmol) in ethanol (45 mL) was added potassium hydroxide (2.96 g, 45 mmol) under reflux for 6 h, then the reaction mixture was concentrated in vacuo and water (90 mL) was added. Solid precipitate was filtered off and washed with water to give compound 9a'-hydroxy-1',3'-diphenyl-1'*H*-spiro[indole-2,2'-pyrrolo[1,2-a]indole]-3,9'(1*H*,9a'*H*)-dione **3** and other mixtures. The residue was purified by flash column chromatography on silica gel (C_6H_6/MeOH, 5/1, v/v) to give **3** (144 mg, 14% yield) as a yellow crystalles: m.p. 258-260 °C. The mother liquor was extracted with ether (4×20mL). The organic phases were combined and the solvent was removed in vacuo. The residue was purified by flash column chromatography on silica gel (C_6H_6/acetone, 5/2, v/v)) to give 3-benzyl-3-hydroxy-1,3-dihydro-2*H*-indol-2-one **4** (54 mg, 5% yield) as a white powder: m.p. 163-165 °C (C_6H_6) (lit.[4] 162-164 °C). The aqueous phase was acidified with 5% hydrochloric acid to pH 5.0-6.0, the precipitate formed was filtered off and washed with water to give **2a** (16 mg, 2% yield).

Method B: To a solution of **1a** (1.0 g, 4.5 mmol) in methanol (45 mL) was added potassium hydroxide (2.96 g, 45 mmol) under reflux, then the reaction mixture was concentrated in vacuo and water (90 mL) was added. Solid precipitate was filtered

off and washed with water to give compound 9a'-hydroxy-1',3'-diphenyl-1'H-spiro[indole-2,2'-pyrrolo[1,2-a]indole]-3,9'(1H,9a'H)-dione **3** and other mixtures. The residue was purified by flash column chromatography on silica gel (C_6H_6/MeOH, 5/1, v/v) to give **3** (144 mg, 14% yield) as a yellow crystalles: m.p. 258-260 °C. The mother liquor was extracted with ether (4×20mL). The organic phases were combined and the solvent was removed in vacuo. The residue was purified by flash column chromatography on silica gel (C_6H_6/acetone, 5/2, v/v) to give 3-hydroxy-3-[methoxy(phenyl)methyl]-1,3-dihydro-2*H*-indol-2-one **5** (151 mg, 12% yield) as a white powder: m.p. 214-216 °C (C_6H_6). **5** was obtained as a mixture of isomers, single isomer was isolated after recrystallization from methanol. The aqueous layer was acidified with 5% hydrochloric acid, the precipitate formed was filtered off and washed with water to give **2a** (150 mg, 7% yield).

Data of compounds 3-5.

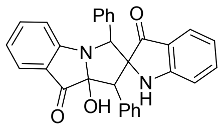

3

^{1}H NMR (600 MHz, DMSO-d_6) δ 3.22 (s, 1H), 4.72 (s, 1H), 6.40 (t, *J* = 7.3 Hz, 1H), 6.61 (d, *J* = 8.2 Hz, 1H), 6.69 (d, *J* = 8.2 Hz, 1H,), 7.07-7.11 (m, 4H), 7.13 (t, *J* = 7.5 Hz, 1H), 7.16 (d, *J* = 7.7 Hz, 1H), 7.23 (t, *J* = 7.0 Hz, 1H), 7.26-7.31 (m, 4H), 7.43 (d, *J* = 5.9 Hz, 2H), 7.47 (s, 1H), 7.58-7.63 (m, 3H); ^{13}C NMR (151 MHz, DMSO-d_6) δ 199.39, 197.69, 161.70, 161.58, 138.77, 138.26, 138.02, 132.43, 131.21, 128.57, 128.09, 127.82, 127.71, 127.16, 125.07, 123.78, 121.98, 121.83, 118.36, 117.15, 113.17, 112.30, 98.09, 83.45, 74.86, 57.10; IR (KBr, cm^{-1}) 3382, 3288, 3063, 3026, 1686, 1605, 1585, 1491, 1464, 1322, 1299, 1260, 1150, 1096, 1077, 1020, 935, 750, 698; MS, m/z [M]$^+$: 458; Anal. Calcd. for $C_{30}H_{22}N_2O_3$: C, 78.59; H, 4.84; N, 6.11. Found: C, 78.31; H, 4.74; N, 6.06.

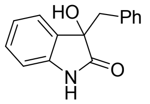

4

^{1}H NMR (400 MHz, DMSO-d_6) δ 3.01 (d, *J* = 12.6 Hz, 1H), 3.14 (d, *J* = 12.6 Hz, 1H), 6.60 (d, *J* = 7.8 Hz, 1H), 6.89-7.12 (m, 8H), 10.04 (s, 1H); ^{13}C NMR (101 MHz, DMSO-d_6) δ 179.21, 142.12, 135.54, 131.41, 130.58, 129.31, 127.93, 126.78, 125.03, 121.64, 109.73, 77.03, 43.92; IR (KBr, cm^{-1}) 3338, 3178, 3085, 3030, 1709, 1670, 1625, 1608, 1473, 1217, 1180, 1112, 756, 700; MS, m/z [M]$^+$: 239; Anal. Calcd. for $C_{15}H_{13}NO_2$: C, 75.30; H, 5.48; N, 5.85. Found: C, 75.08; H, 5.31; N, 5.98.

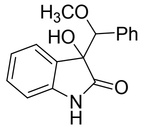

5

^{1}H NMR (400 MHz, DMSO-d_6) δ 3.07 (s, 3H), 4.54 (s, 1H), 6.05 (d, *J* = 7.2 Hz, 1H), 6.18 (s, 1H), 6.70 (t, *J* = 7.5 Hz, 1H), 6.77 (d, *J* = 7.6 Hz, 1H), 7.12-7.36 (m, 6H), 10.24 (s, 1H); ^{13}C NMR for mixture of isomers (75 MHz, DMSO-d_6) δ 179.55, 177.46, 143.75, 142.54, 136.67, 136.51, 129.50, 129.31, 128.76, 128.42, 128.20, 127.75, 126.79, 126.30, 121.58, 120.80, 109.74, 109.39, 87.34, 87.22, 85.55, 85.46, 79.49, 77.37, 57.94, 57.43; IR (KBr, cm^{-1}) 3326, 3276, 3059, 1708, 1624, 1489, 1472, 1453, 1359, 1350, 1214, 1185, 1132, 1101, 1077, 975, 754, 727, 699, 674, 599; MS, m/z [M]$^+$: 269; Anal. Calcd. for $C_{16}H_{15}NO_3$: C, 71.36; H, 5.61; N, 5.20. Found: C, 71.64; H, 5.49; N, 5.11.

5,12-Diphenyl-5,13-dihydro-11*H*-pyrido[1,2-a:4,5-b']diindol-11-one (6a).

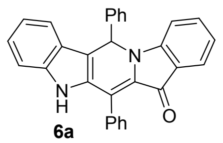

To a solution of **1a** (260 mg, 1.18 mmol) in *tert*-BuOH (3.2 mL) was added potassium hydroxide (260 mg, 4.02 mmol) and the mixture was stirred at room temperature for 17 h. Water (3-5 mL) was added to a precipitate, the aqueous layer was extracted with benzene (3×20 mL) or ethyl acetate (2×50 mL), the organic layers were combined and concentrated in vacuo. The residue was purified by flash column chromatography on silica gel (C_6H_6/MeOH, 5/1, v/v) to give 5,12-diphenyl-5,13-dihydro-11*H*-pyrido[1,2-a:4,5-b']diindol-11-one **6** (177 mg, 83% yield) as a blue powder: m.p. 207-209 °C (C_6H_6/MeOH). ^{1}H NMR (600 MHz, DMSO-d_6) δ 6.88 (t, *J* = 7.4 Hz, 1H), 6.98 (t, *J* = 7.5 Hz, 1H), 7.02 (s, 1H), 7.08-7.58 (m, 12H), 7.61 (d, *J* = 7.0 Hz, 2H), 7.64 (d, *J* = 7.6 Hz, 2H), 10.89 (s, 1H); ^{13}C NMR (151 MHz, DMSO-d_6) δ 182.29, 150.70, 143.05, 139.51, 135.70, 131.86, 130.52, 130.42, 130.17, 129.32, 128.98, 128.87, 128.34, 127.07, 124.82, 124.19, 123.84, 123.19, 120.61, 120.10, 119.29, 118.55, 113.07, 112.90, 111.39, 56.66; IR (KBr, cm^{-1}) 3431, 3281, 2921, 2851, 1651, 1614, 1584, 1566, 1511, 1474, 1435, 1346, 1326, 1295, 1270, 1239, 1189, 1153, 1134, 1101, 1063, 980, 744, 721, 560, 475, 429, 403; MS, m/z [M]$^+$: 424; Anal. Calcd. for $C_{30}H_{20}N_2O$: C, 84.88; H, 4.75; N, 6.60. Found: C, 84.74; H, 4.98; N, 6.43.

5,12-Diphenyl-11*H*-pyrido[1,2-a:4,5-b']diindol-11-one (7a).

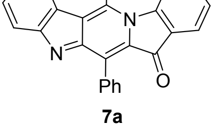

7a

To a solution of **6** (84 mg, 0.198 mmol) in ethanol (10 mL) under reflux was added potassium hydroxide (150 mg, 2.3 mmol) and the mixture was refluxed for 32h. Reaction mixture was acidified with hydrochloric acid to pH 2.0-3.0. The solid precipitate was filtered off. The organic layer was diluted with water (10-20 mL) and was added 86 % potassium hydroxide to pH 8.0-10.0. The precipitate was filtered off, washed with water, dried on air. The residue was purified by flash column

chromatography on silica gel (C_6H_6/MeOH, 5/1, v/v) to give 5,12-diphenyl-11*H*-pyrido[1,2-a:4,5-b']diindol-11-one **7** (68 mg, 81% yield) as a red powder: m.p. 317-319 °C. ^{1}H NMR (400 MHz, DMSO-d$_6$) δ 6.02 (d, *J* = 8.6 Hz, 1H), 6.56 (d, *J* = 7.6 Hz, 1H), 6.98 (t, *J* = 7.1 Hz, 1H), 7.31 (t, *J* = 7.2 Hz, 1H), 7.35-7.96 (m, 14H); ^{13}C NMR (101 MHz, DMSO-d$_6$) δ 182.62, 162.80, 158.07, 154.83, 147.72, 141.90, 136.37, 132.37, 131.84, 131.81, 131.14, 130.68, 129.44, 129.14, 129.01, 128.04, 127.92, 127.41, 126.82, 126.64, 125.16, 122.79, 122.22, 121.54, 120.66, 116.24; IR (KBr, cm^{-1}) 3050, 1703, 1608, 1582, 1541, 1466, 1443, 1362, 1346, 1297, 1221, 1209, 1094, 996, 769, 755, 737, 697; MS, m/z [M]$^+$: 422; Anal. Calcd for $C_{30}H_{18}N_2O$: C, 85.29; H, 4.29; N, 6.63. Found: C, 85.37; H, 4.33; N, 6.54.

5,12-Bis(4-fluorophenyl)-5,13-dihydro-11*H*-pyrido[1,2-a:4,5-b']diindol-11-one (6c).

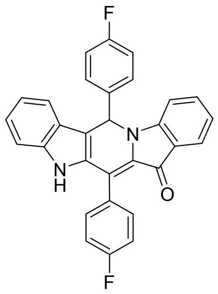

6c

To a solution of **1a** (740 mg, 3.10 mmol) in *tert*-BuOH (8.5 mL) was added potassium hydroxide (647 mg, 11.55 mmol) and the mixture was stirred at room temperature for 21 h. After reaction completion, the water (15 mL) was added, the mixture was acidified with CH_3COOH to pH 7. The resulting solid precipitate was filtered off, washed with water and dried. The residue was recrystallized from MeOH to give 5,12-bis(4-fluorophenyl)-5,13-dihydro-11*H*-pyrido[1,2-a:4,5-b']diindol-11-one **6c** (330 mg, 46% yield) as a purple powder: m.p. 243-246 °C (MeOH). ^{1}H NMR (600 MHz, DMSO-d$_6$) δ 6.88 (t, *J* = 7.2 Hz, 1H), 6.98 (br s, 2H), 7.10 (t, *J* = 7.8 Hz, 4H), 7.34 (q, *J* = 8.7 Hz, 3H), 7.42-7.49 (m, 3H), 7.64 (t, *J* = 6.1 Hz, 4H), 10.93 (s, 1H); ^{13}C NMR (126 MHz, DMSO-d$_6$) δ 182.50, 163.80, 162.95, 161.85, 161.00, 150.57, 139.46, 139.16, 139.14, 135.96, 132.59, 130.48, 130.02, 129.20, 127.92, 127.90, 124.70, 123.07, 120.85, 120.40, 119.25, 117.69, 116.19, 115.99, 115.75, 113.04, 112.81, 111.40; MS, m/z [M]$^+$: 460.

5,12- Bis(4-fluorophenyl)-11*H*-pyrido[1,2-a:4,5-b']diindol-11-one (7c).

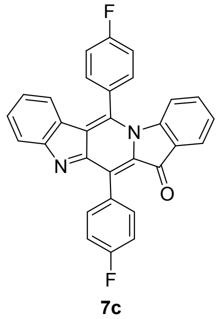

7c

To a solution of **6c** (106 mg, 0.25 mmol) in ethanol (10 mL) under reflux was added potassium hydroxide (205 mg, 4.4 mmol) and the mixture was refluxed for 40h. Reaction mixture was acidified with hydrochloric acid to pH 2.0. The solid precipitate was filtered off. The organic layer was diluted with water (10-20 mL) and was added 86 % potassium hydroxide to pH 8.0-10.0. The precipitate was filtered off, washed with water, dried on air. The residue was recrystallized from DMF/EtOH to give 5,12-bis(4-fluorophenyl)-11*H*-pyrido[1,2-a:4,5-b']diindol-11-one **7c** (42 mg, 40% yield) as a red powder: m.p. 395 °C (decomp.). ^{1}H NMR (600 MHz, DMSO-d$_6$) δ 6.07 (d, *J* = 8.6, 1H), 6.57 (d, *J* = 8.1, 1H), 7.02 (t, *J* = 7.5, 1H), 7.31-7.44 (m, 6H), 7.69-7.73 (m, 6H), 7.92 (q, J = 2.9Hz, 2H); ^{13}C NMR (126 MHz, DMSO-d$_6$) δ 182.83, 165.12, 163.94, 163.14, 161.99, 158.15, 157.79, 154.64, 147.60, 141.18, 136.68, 133.38, 133.32, 131.80, 131.73, 129.35, 128.54, 128.52, 128.26, 128.21, 127.76, 127.66, 126.60, 126.52, 125.34, 123.02, 122.54, 121.50, 120.63, 118.11, 117.94, 116.31, 115.24, 115.06; MS, m/z [M]$^+$: 458.

3. Copies of NMR Spectra

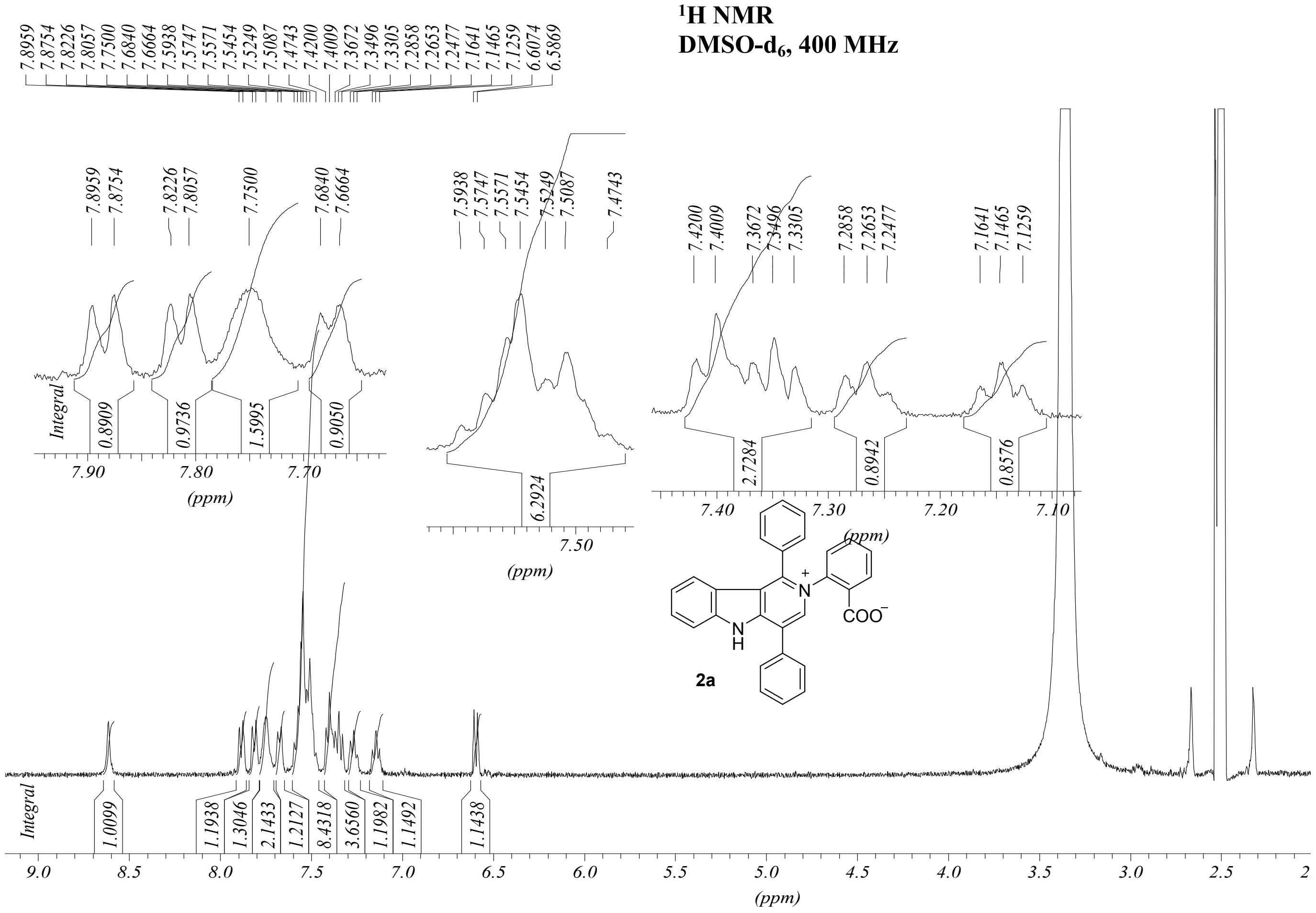

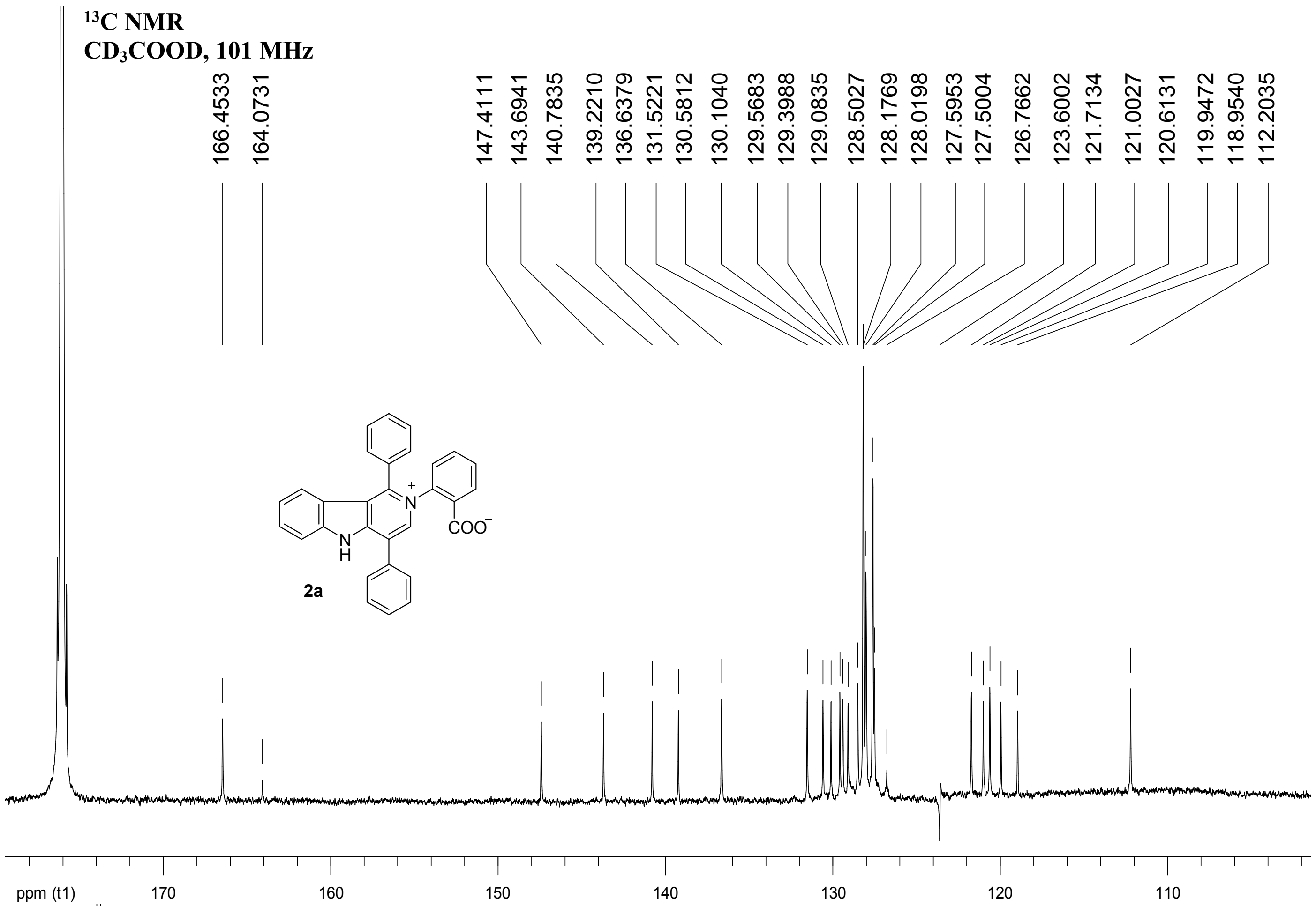
13C NMR
CD3COOD, 101 MHz
166.4533
164.0731
147.4111
143.6941
140.7835
139.2210
136.6379
131.5221
130.5812
130.1040
129.5683
129.3988
129.0835
128.5027
128.1769
128.0198
127.5953
127.5004
126.7662
123.6002
121.7134
121.0027
120.6131
119.9472
118.9540
112.2035
2a
ppm (t1)
170
160
150
140
130
120
110

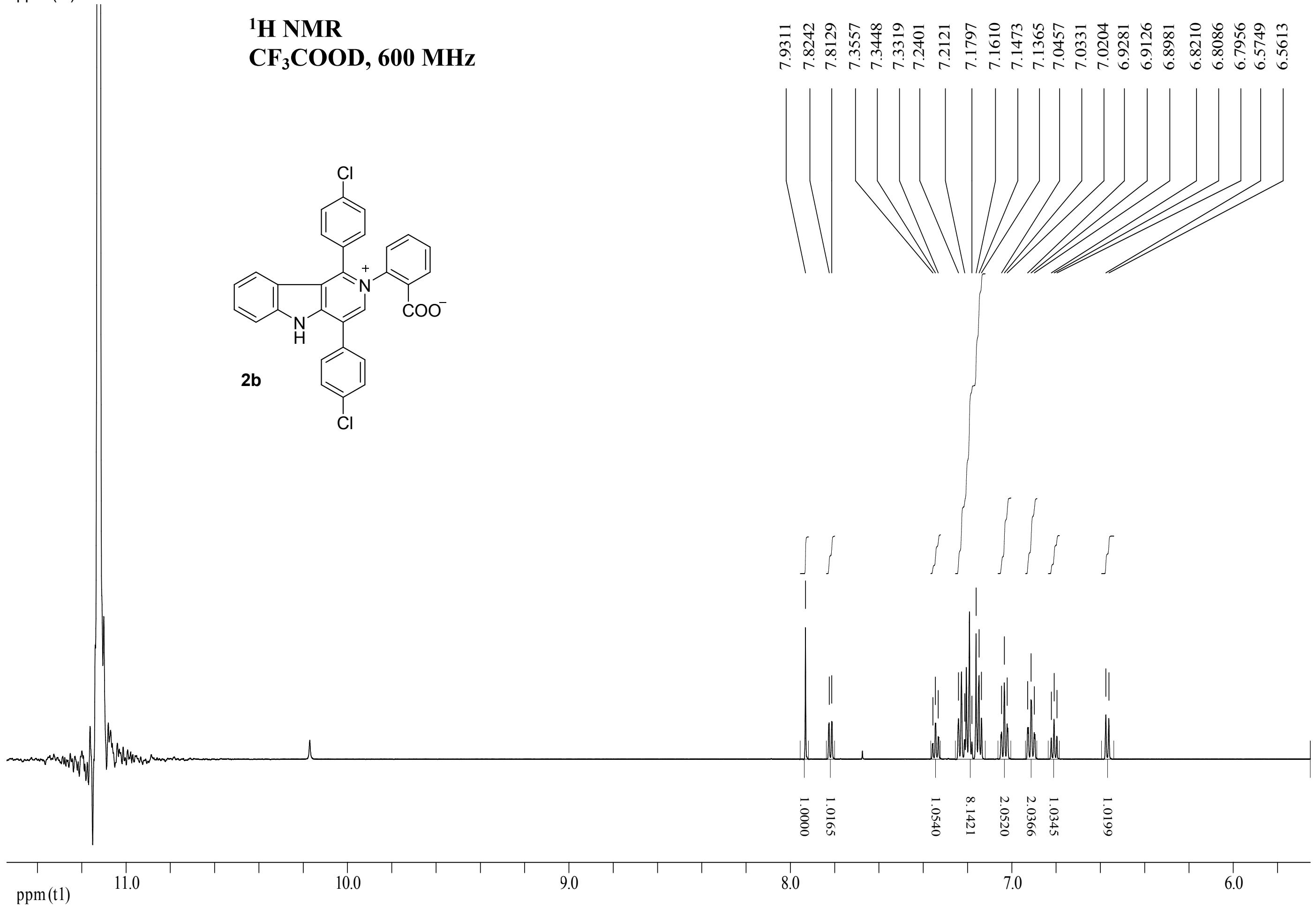
1H NMR
CF3COOD, 600 MHz
7.9311
7.8242
7.8129
7.3557
7.3448
7.3319
7.2401
7.2121
7.1797
7.1610
7.1473
7.1365
7.0457
7.0331
7.0204
6.9281
6.9126
6.8981
6.8210
6.8086
6.7956
6.5749
6.5613
Cl
COO−
2b
1.0000
1.0165
1.0540
8.1421
2.0520
2.0366
1.0345
1.0199
ppm(t1)
11.0
10.0
9.0
8.0
7.0
6.0

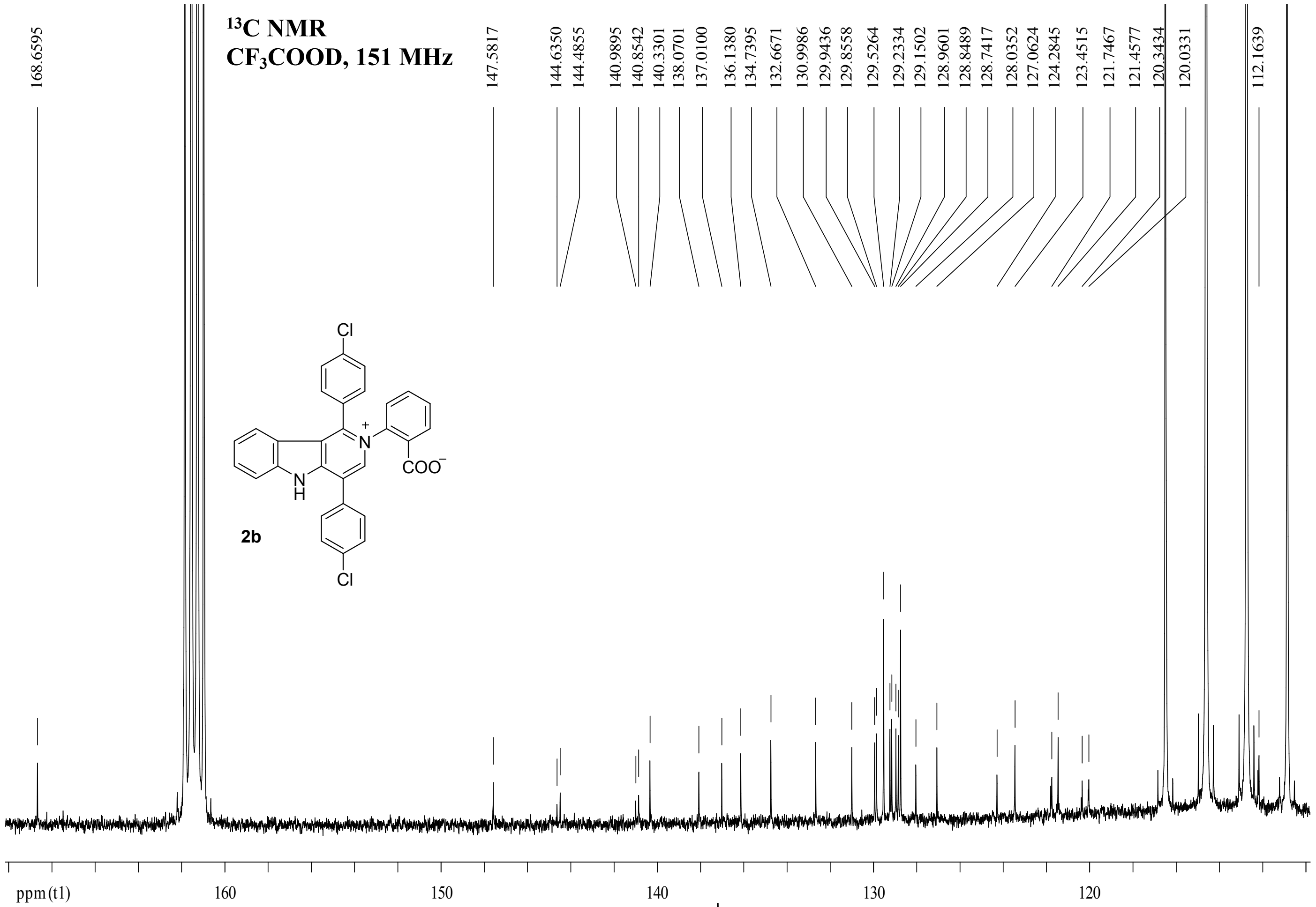
13C NMR
CF3COOD, 151 MHz
168.6595
147.5817
144.6350
144.4855
140.9895
140.8542
140.3301
138.0701
137.0100
136.1380
134.7395
132.6671
130.9986
129.9436
129.8558
129.5264
129.2334
129.1502
128.9601
128.8489
128.7417
128.0352
127.0624
124.2845
123.4515
121.7467
121.4577
120.3434
120.0331
112.1639
Cl
N
H
COO−
2b
Cl
ppm (t1)
160
150
140
130
120

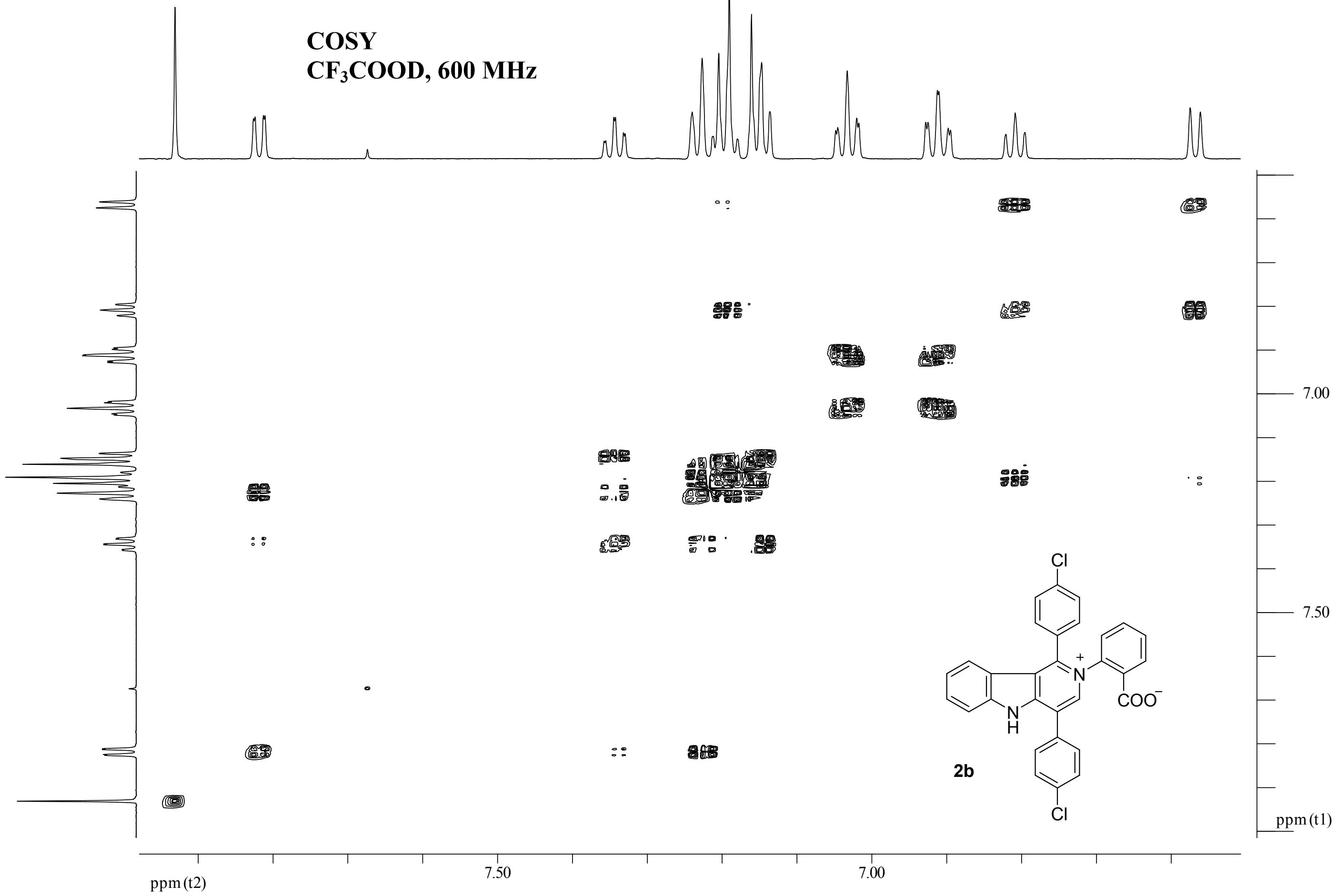
COSY
CF3COOD, 600 MHz
7.00
7.50
ppm (t1)
Cl
N
H
COO−
2b
Cl
7.50
7.00
ppm (t2)

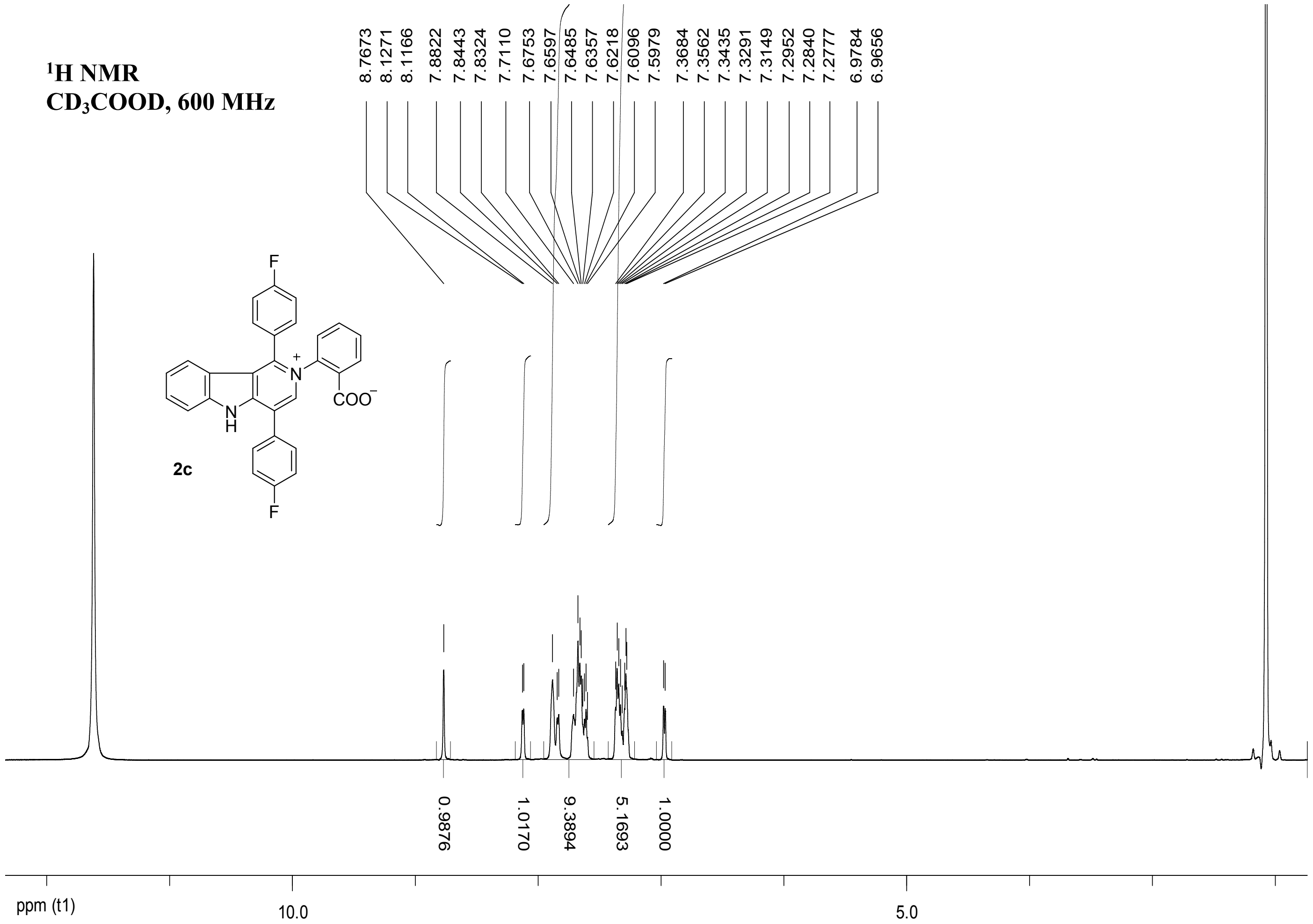

^{1}H NMR
CD_3COOD, 600 MHz
8.7673
8.1271
8.1166
7.8822
7.8443
7.8324
7.7110
7.6753
7.6597
7.6485
7.6357
7.6218
7.6096
7.5979
7.3684
7.3562
7.3435
7.3291
7.3149
7.2952
7.2840
7.2777
6.9784
6.9656
F
N
COO
N
H
F
2c
0.9876
1.0170
9.3894
5.1693
1.0000
ppm (t1)
10.0
5.0

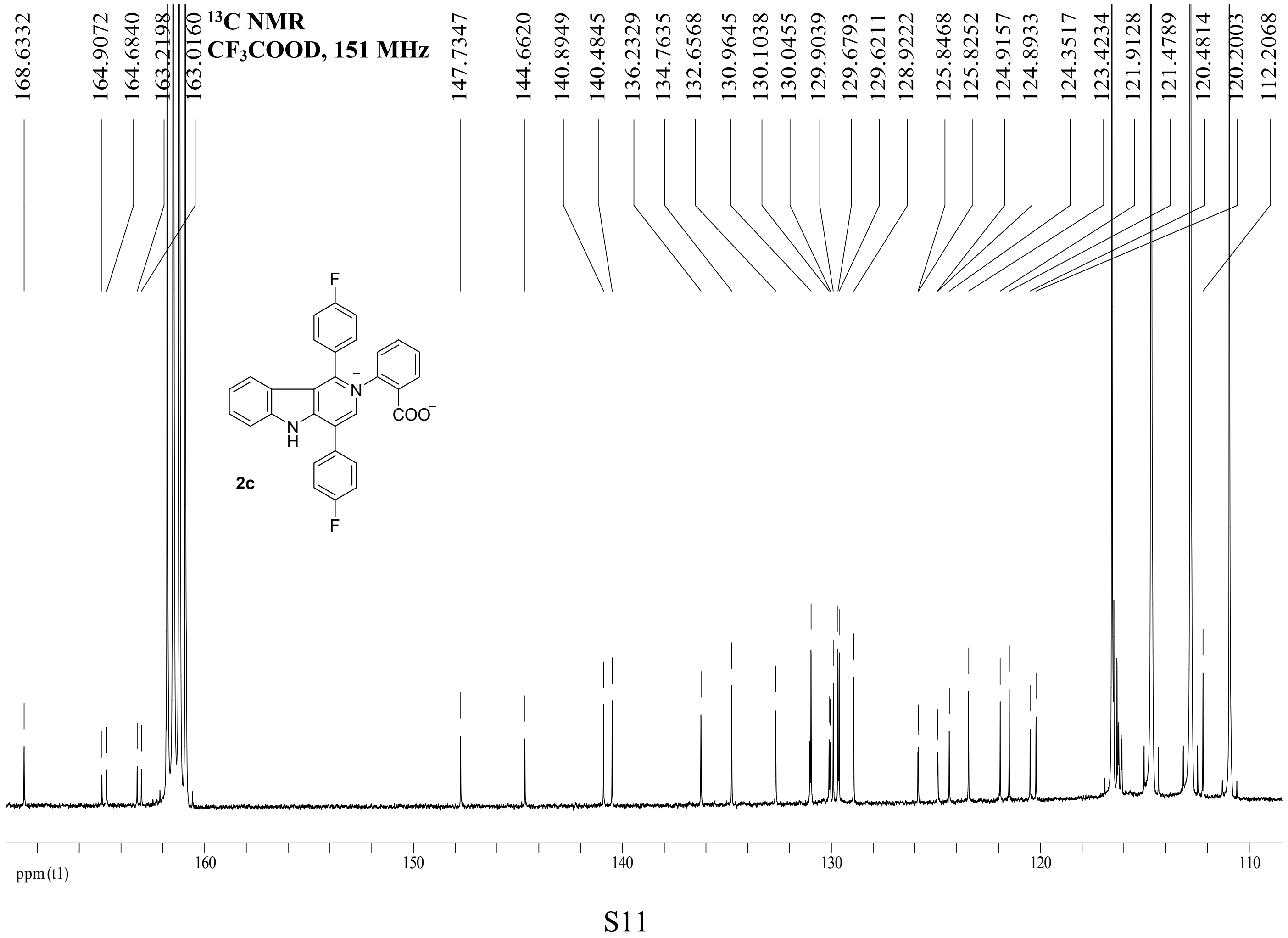

^{13}C NMR
CF_3COOD, 151 MHz
168.6332
164.9072
164.6840
163.2198
163.0160
147.7347
144.6620
140.8949
140.4845
136.2329
134.7635
132.6568
130.9645
130.1038
130.0455
129.9039
129.6793
129.6211
128.9222
125.8468
125.8252
124.9157
124.8933
124.3517
123.4234
121.9128
121.4789
120.4814
120.2003
112.2068
F
N
COO
N
H
F
2c
ppm (t1)
160
150
140
130
120
110

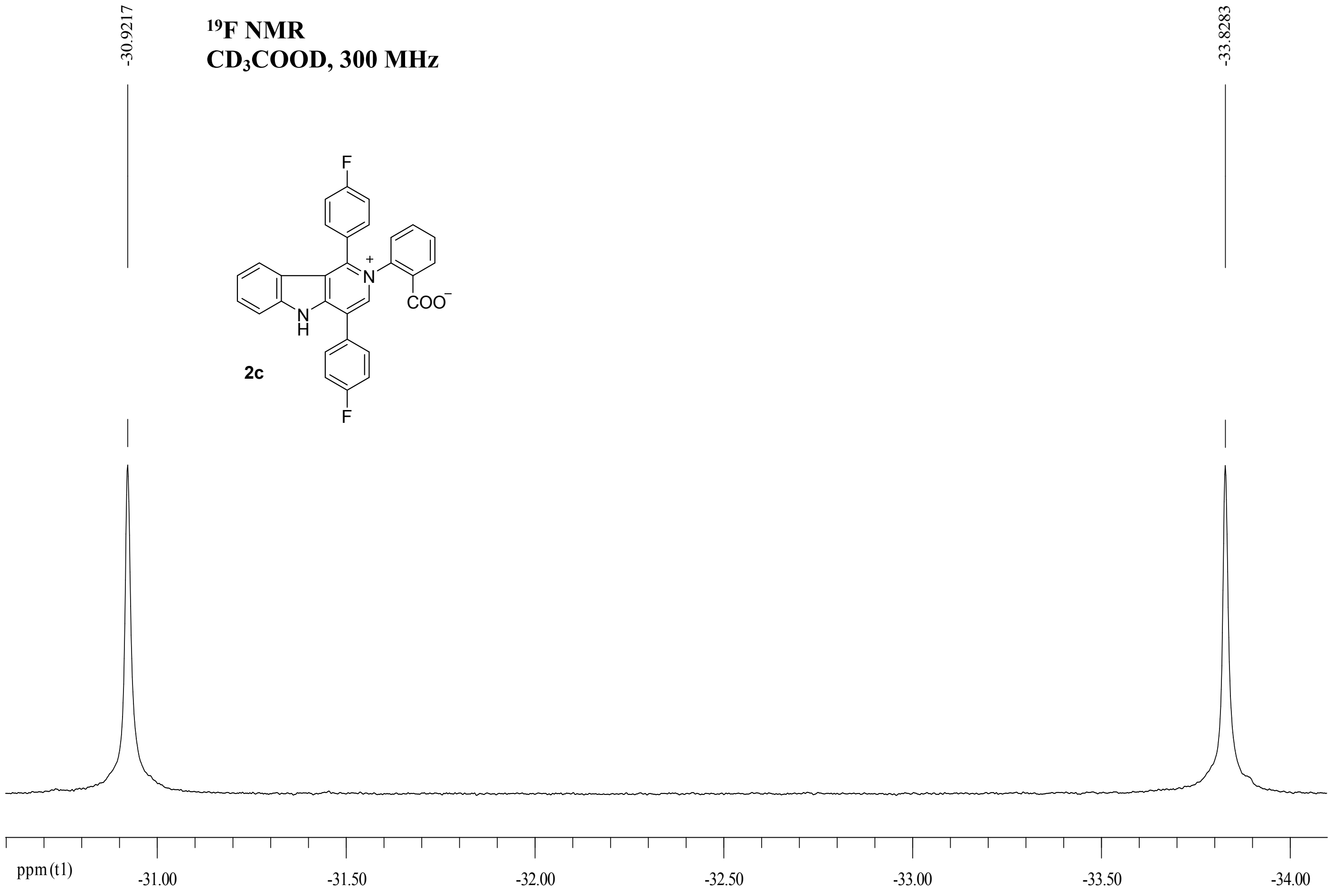
-30.9217
-33.8283
19F NMR
CD3COOD, 300 MHz
F
N+
COO−
N
H
2c
F
ppm (t1)
-31.00
-31.50
-32.00
-32.50
-33.00
-33.50
-34.00

COSY
CD_3COOD, 600 MHz

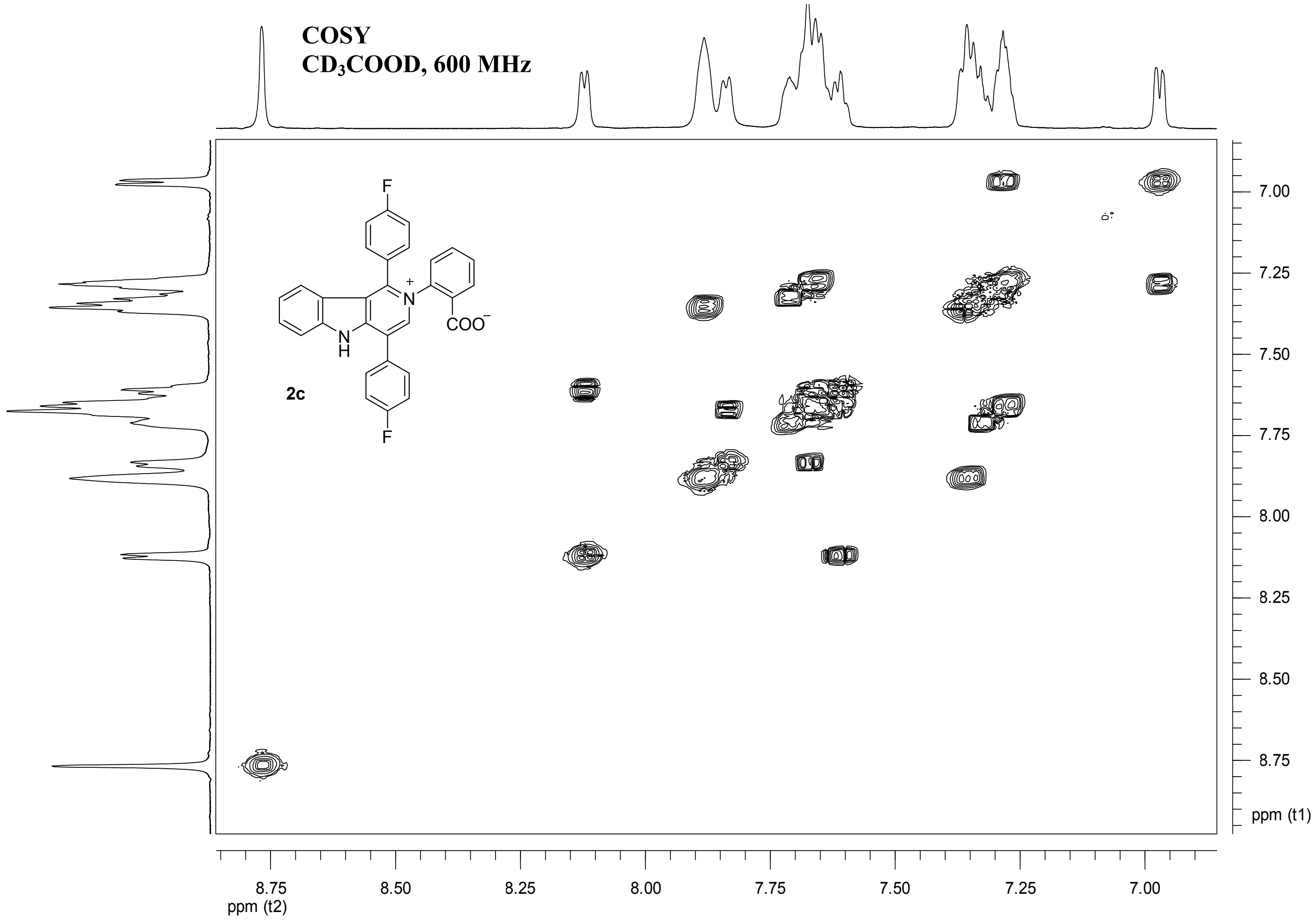

^1H NMR
CD_3COOD, 600 MHz

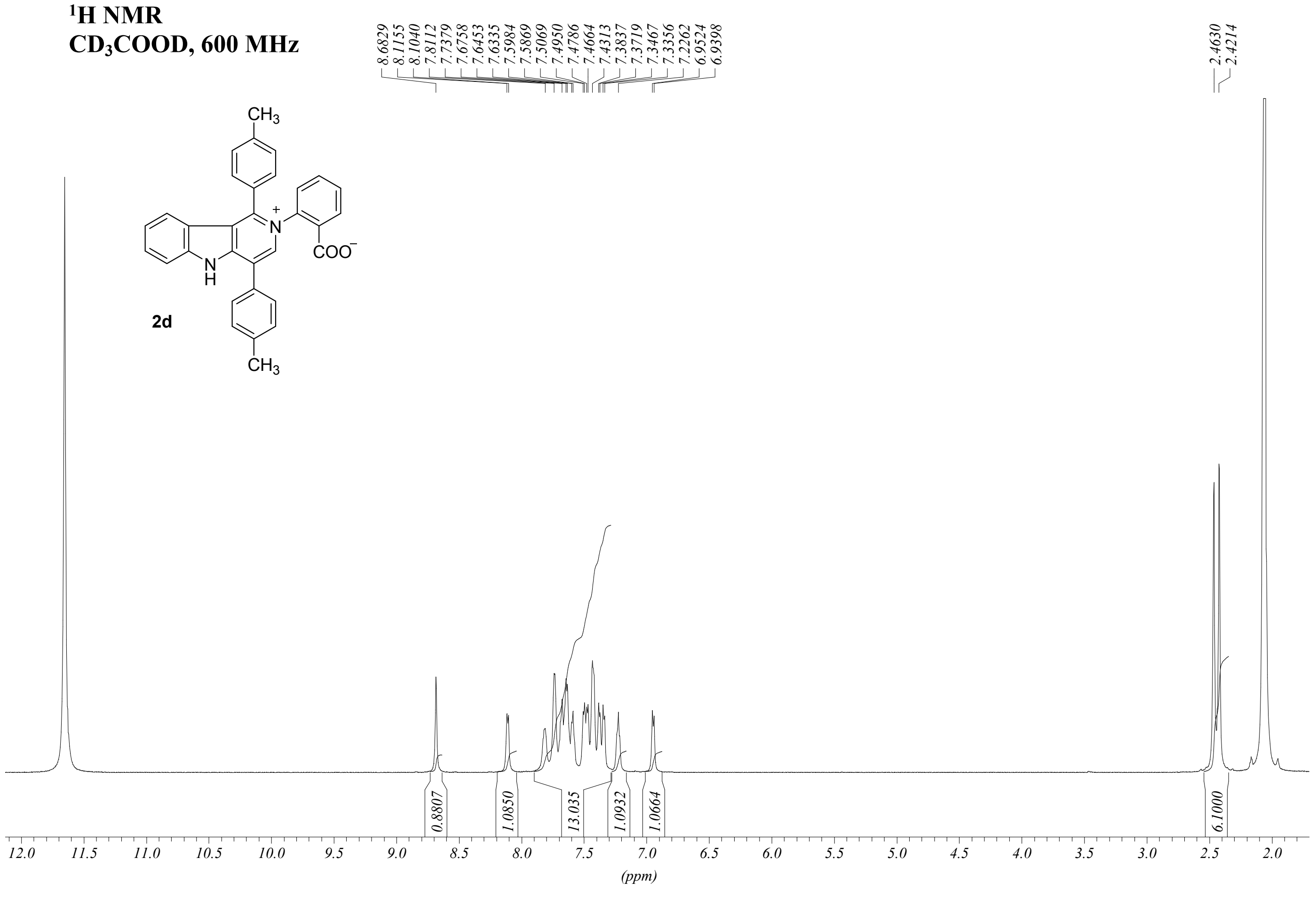

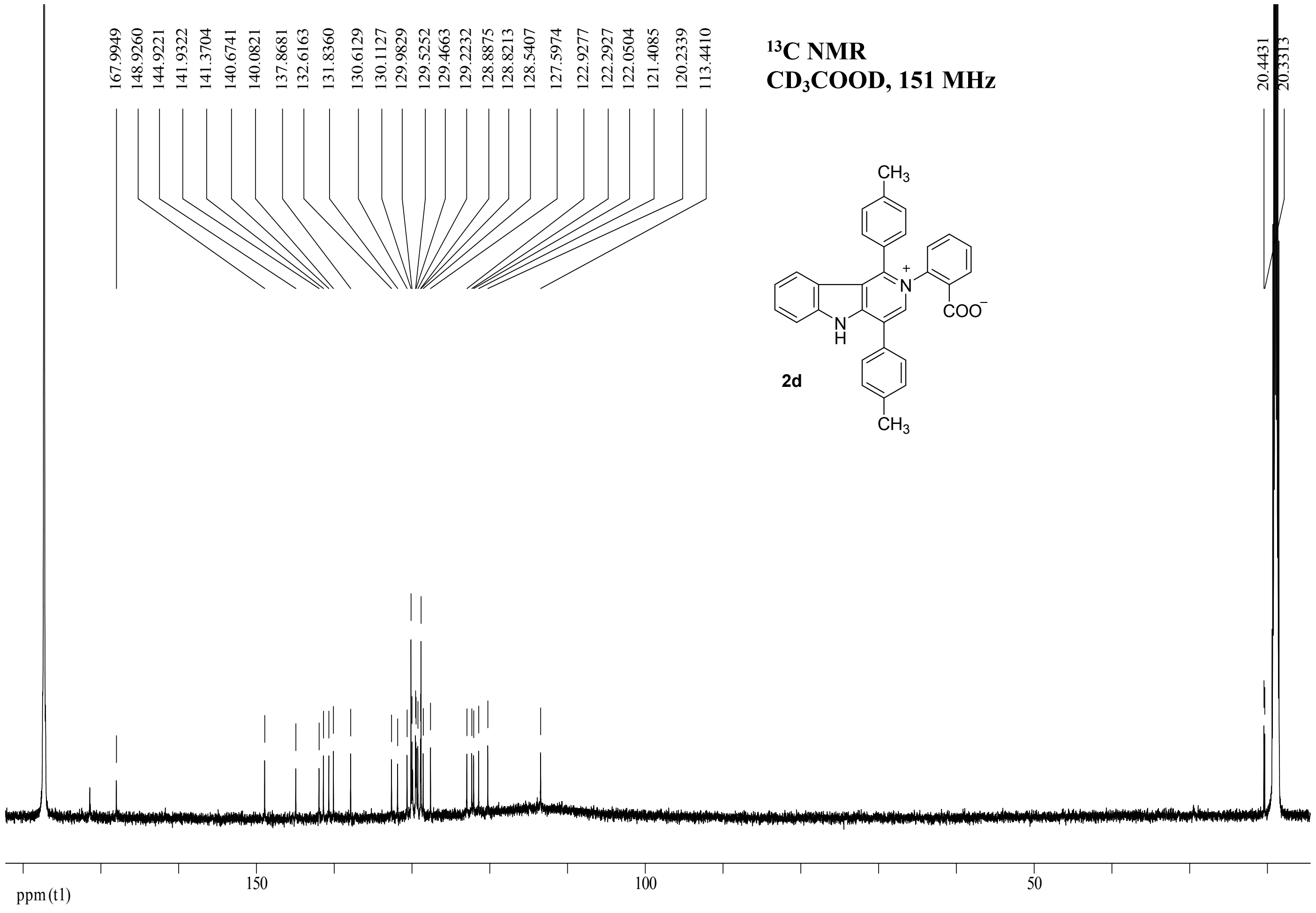

167.9949
148.9260
144.9221
141.9322
141.3704
140.6741
140.0821
137.8681
132.6163
131.8360
130.6129
130.1127
129.9829
129.5252
129.4663
129.2232
128.8875
128.8213
128.5407
127.5974
122.9277
122.2927
122.0504
121.4085
120.2339
113.4410
^{13}C NMR
CD_3COOD, 151 MHz
20.4431
20.3313
2d
150
100
50
ppm (t1)

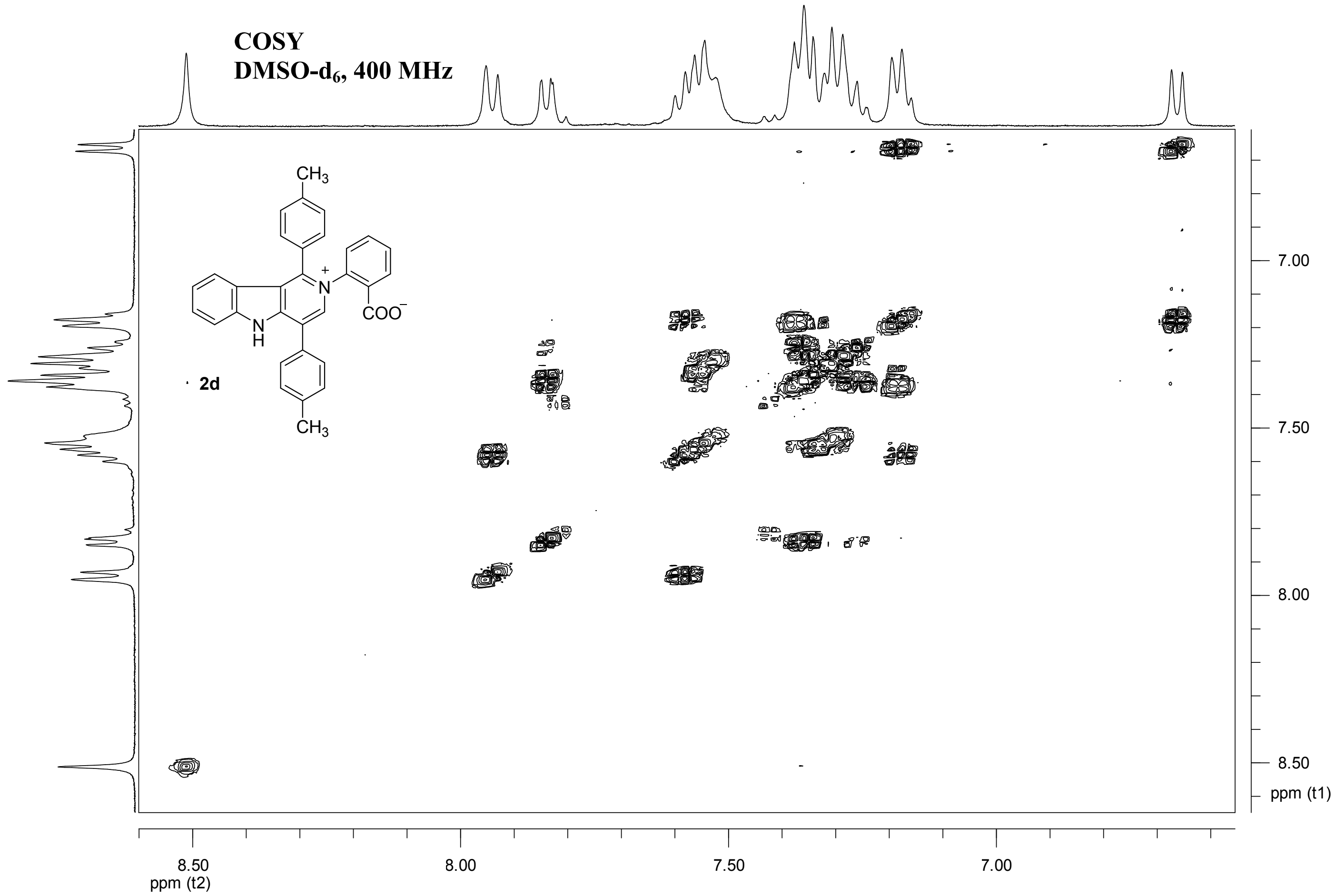

COSY
DMSO-d_6, 400 MHz
2d
7.00
7.50
8.00
8.50
ppm (t1)
8.50
8.00
7.50
7.00
ppm (t2)

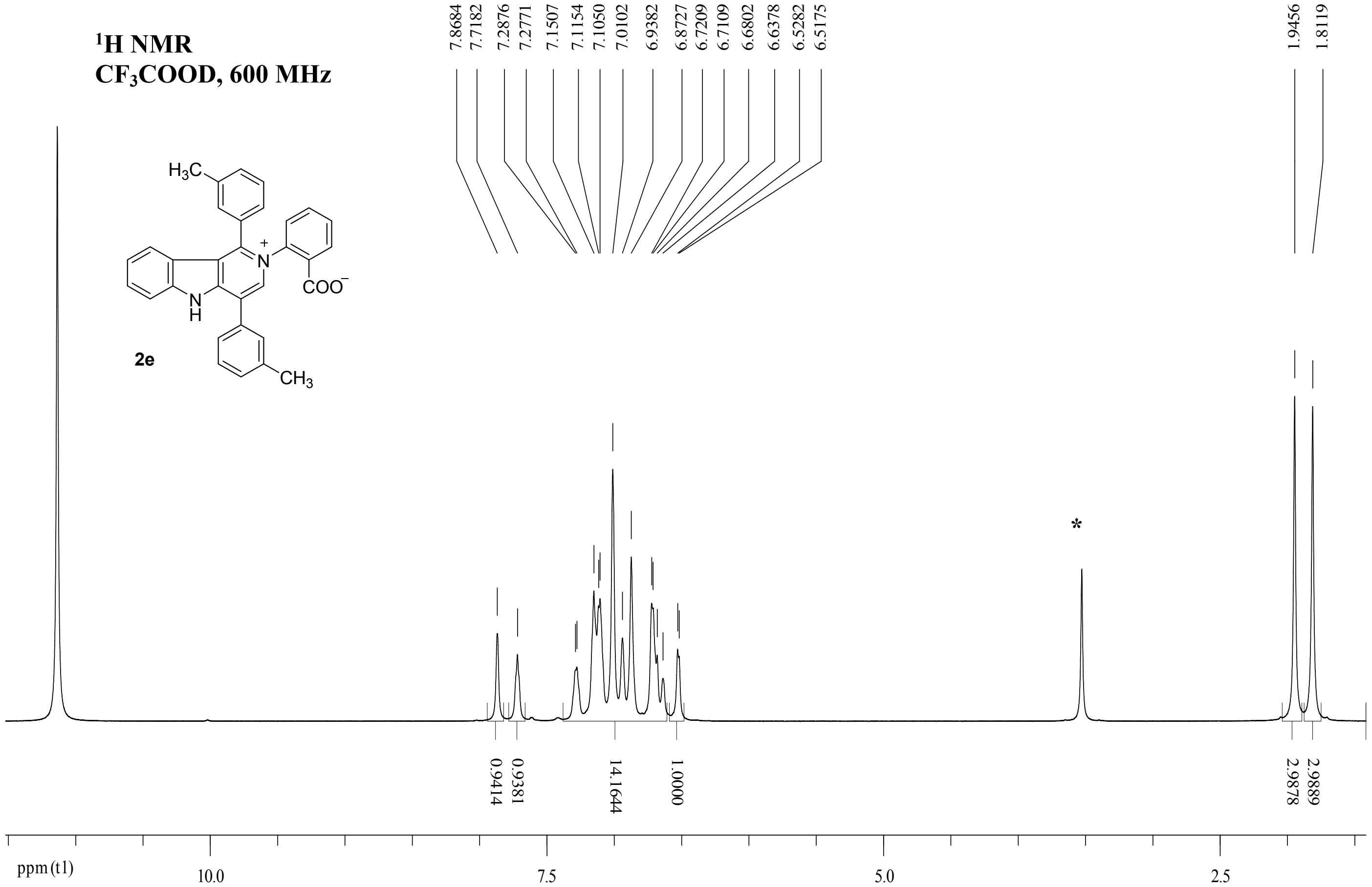

*) from CF_3COOD

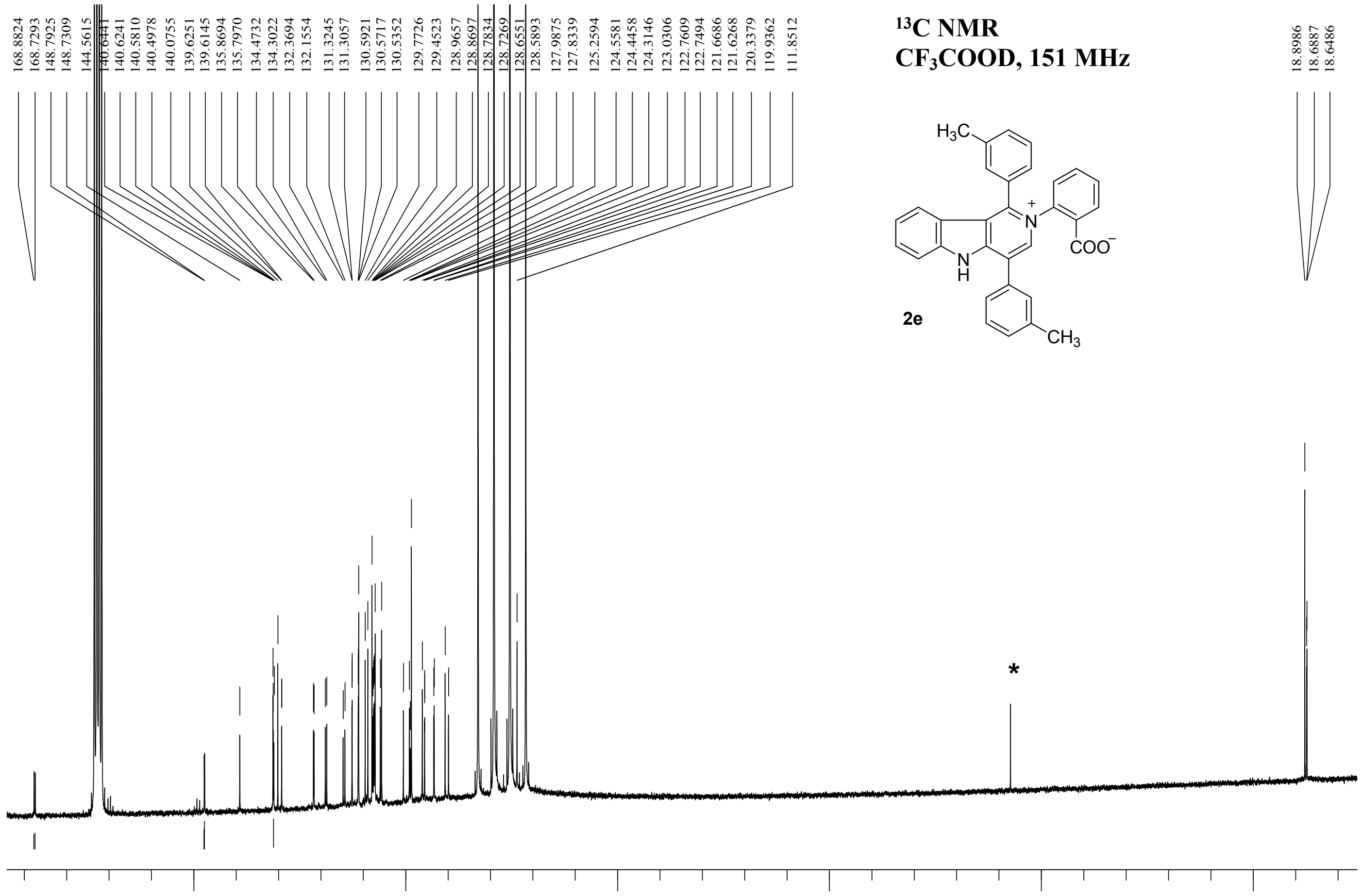

*) from CF_3COOD

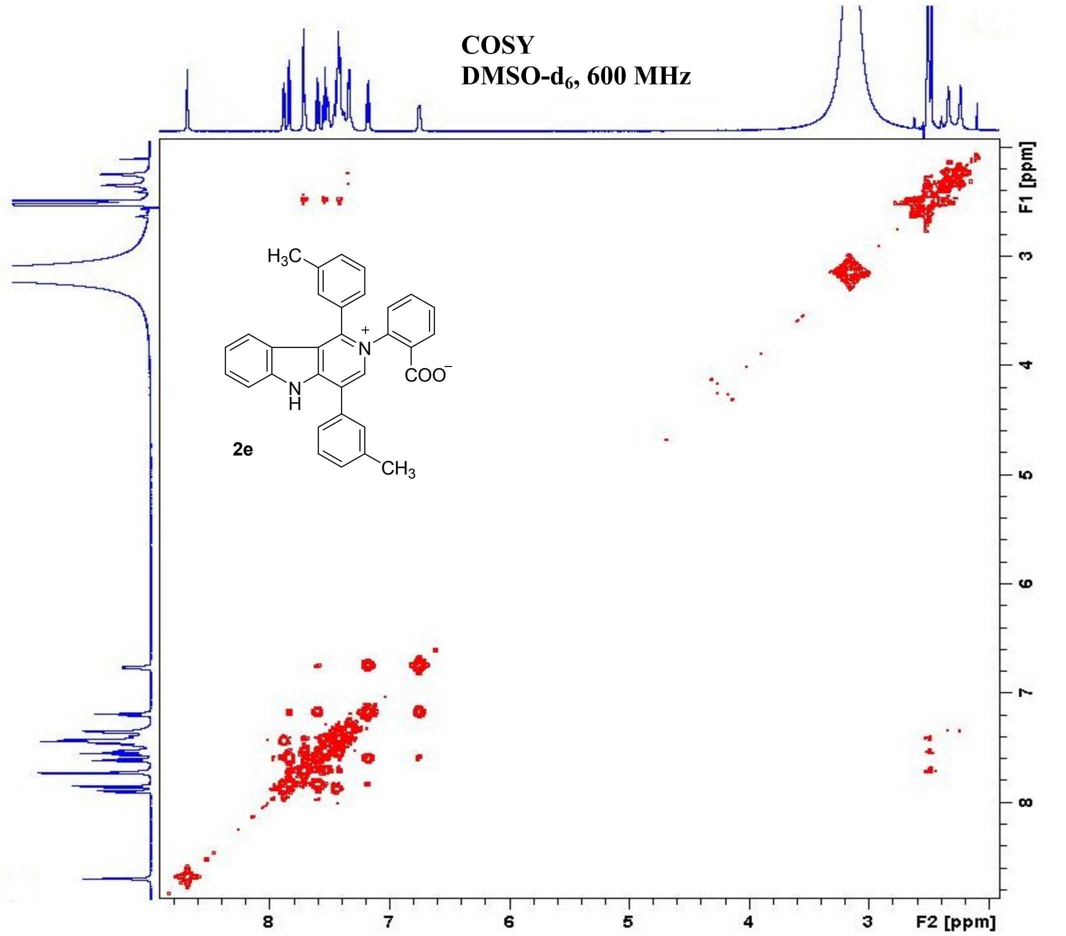
COSY
DMSO-d$_6$, 600 MHz
H_3C
N$^+$
COO$^-$
N
H
2e
CH_3
F1 [ppm]
F2 [ppm]
3
4
5
6
7
8
8
7
6
5
4
3

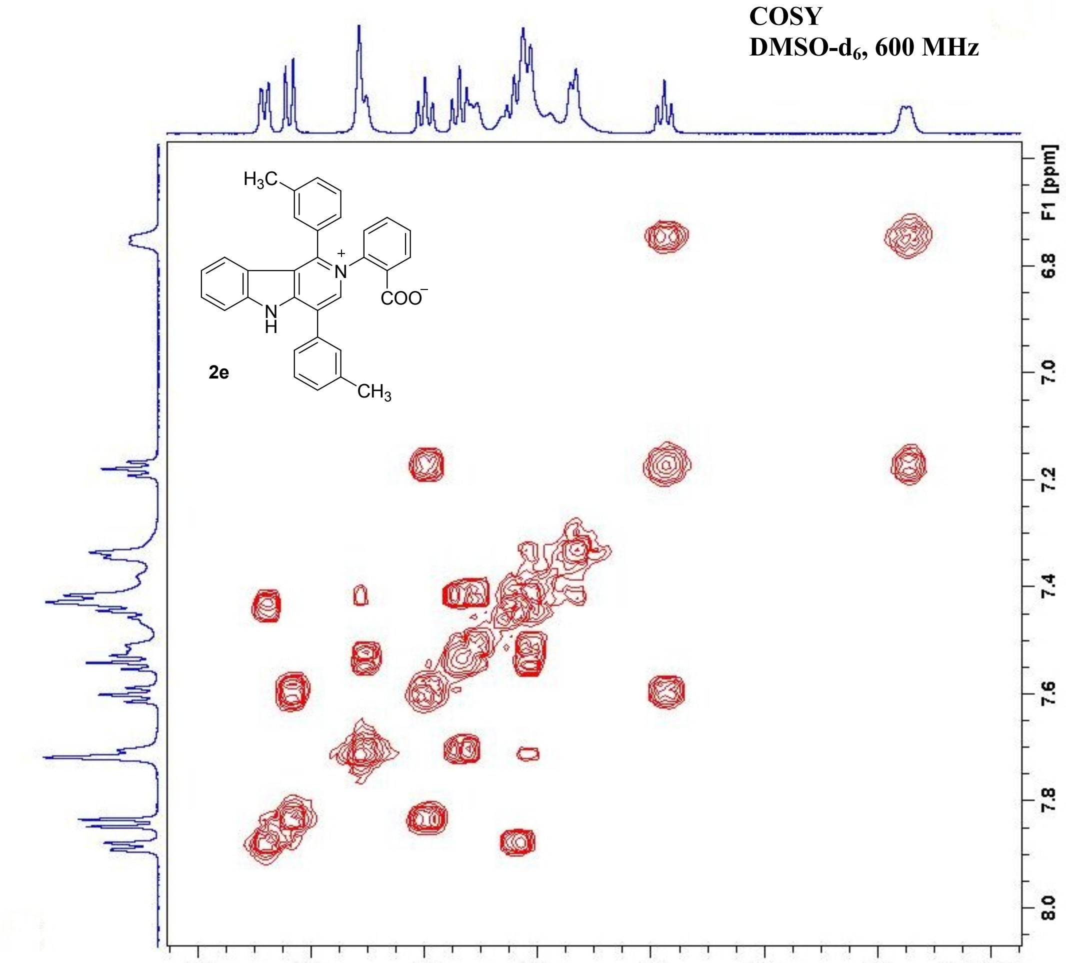
COSY
DMSO-d$_6$, 600 MHz
H_3C
N$^+$
COO$^-$
N
H
2e
CH_3
F1 [ppm]
6.8
7.0
7.2
7.4
7.6
7.8
8.0
F2 [ppm]
8.0
7.8
7.6
7.4
7.2
7.0
6.8

HMQC1
DMSO-d$_6$, 600 MHz

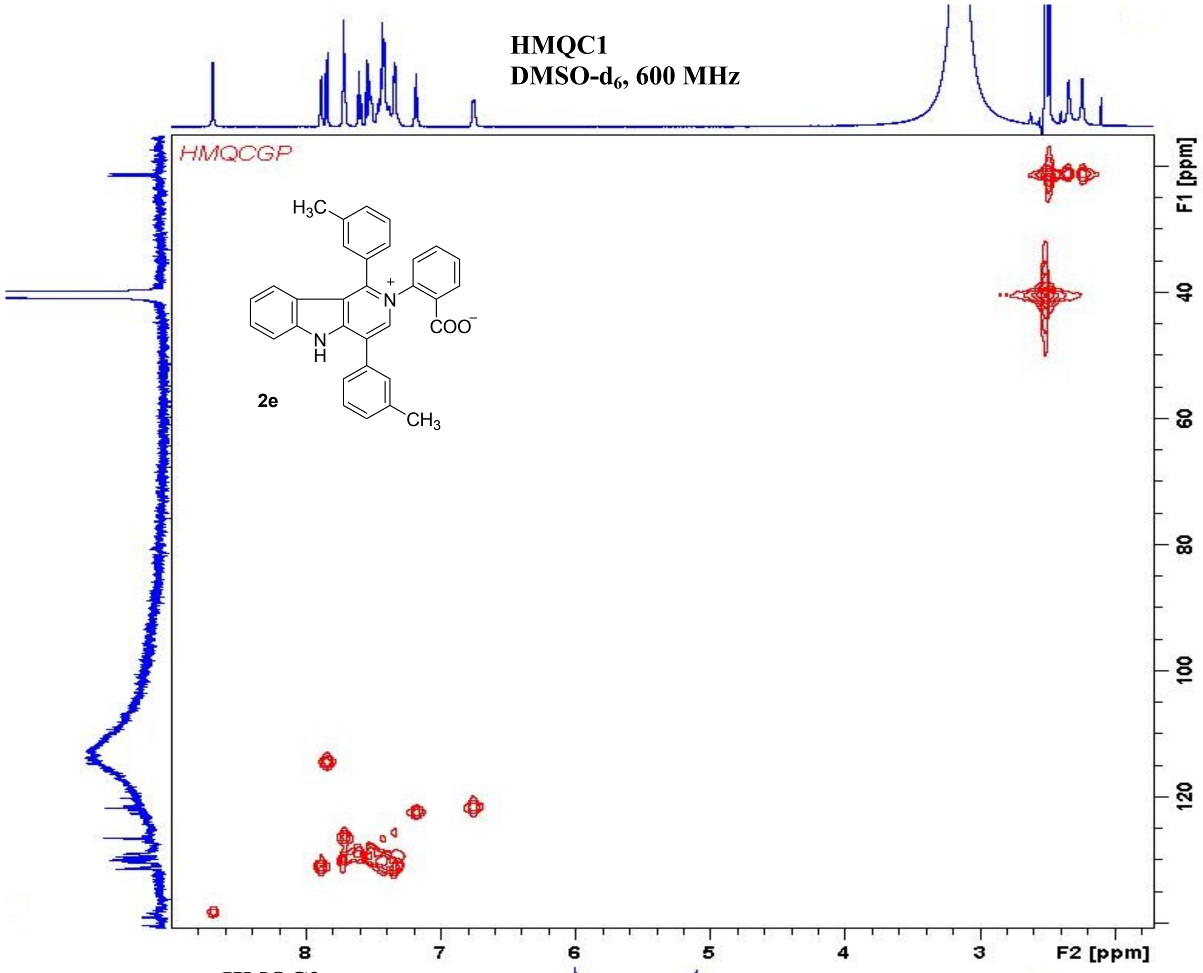

HMQC2
DMSO-d$_6$, 600 MHz

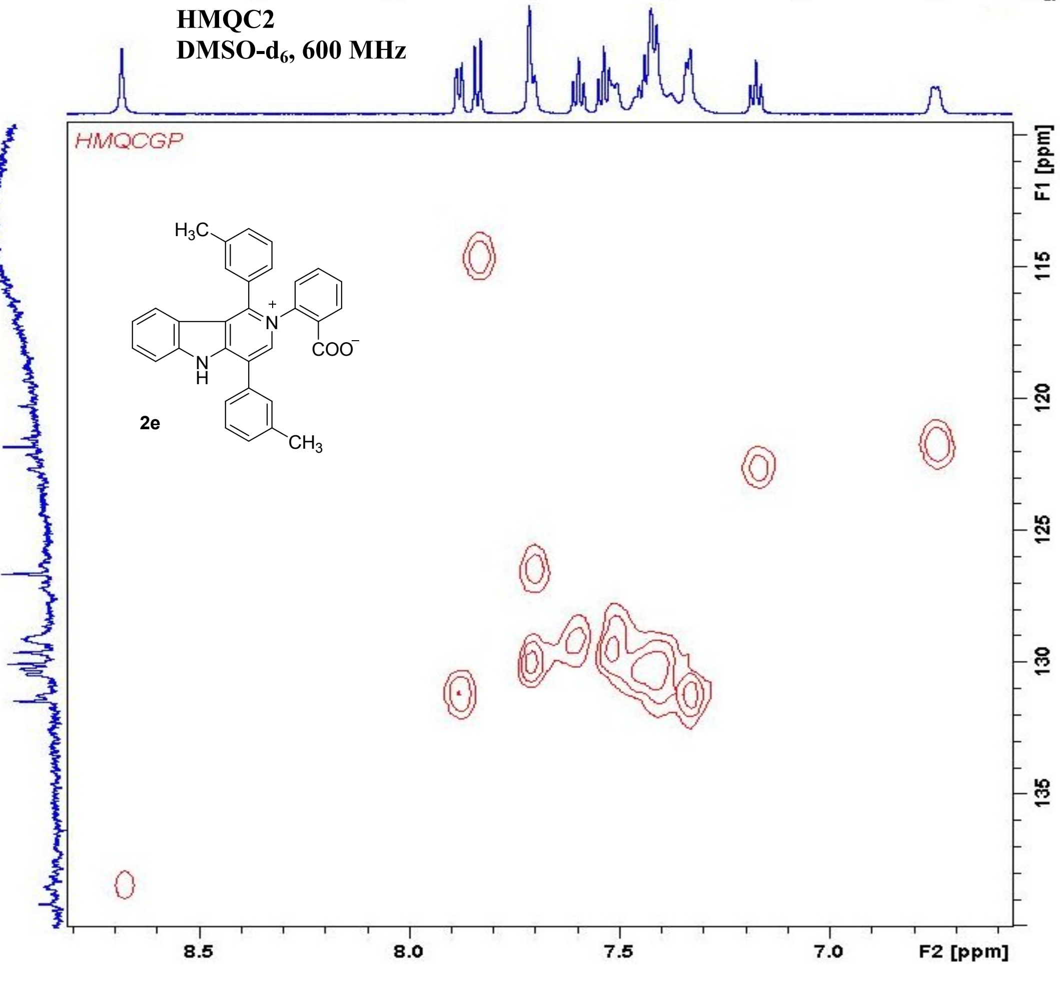

HMQC3
DMSO-d$_6$, 600 MHz

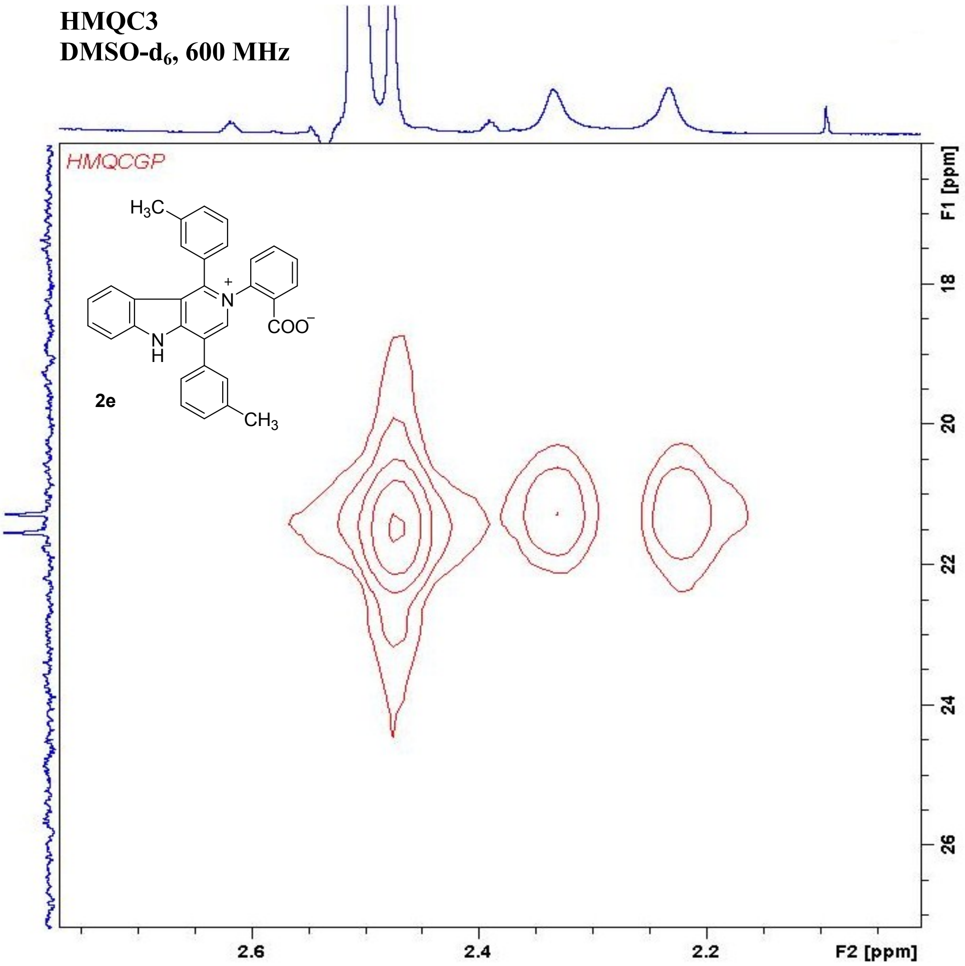

^{1}H NMR
CF$_3$COOD, 600 MHz

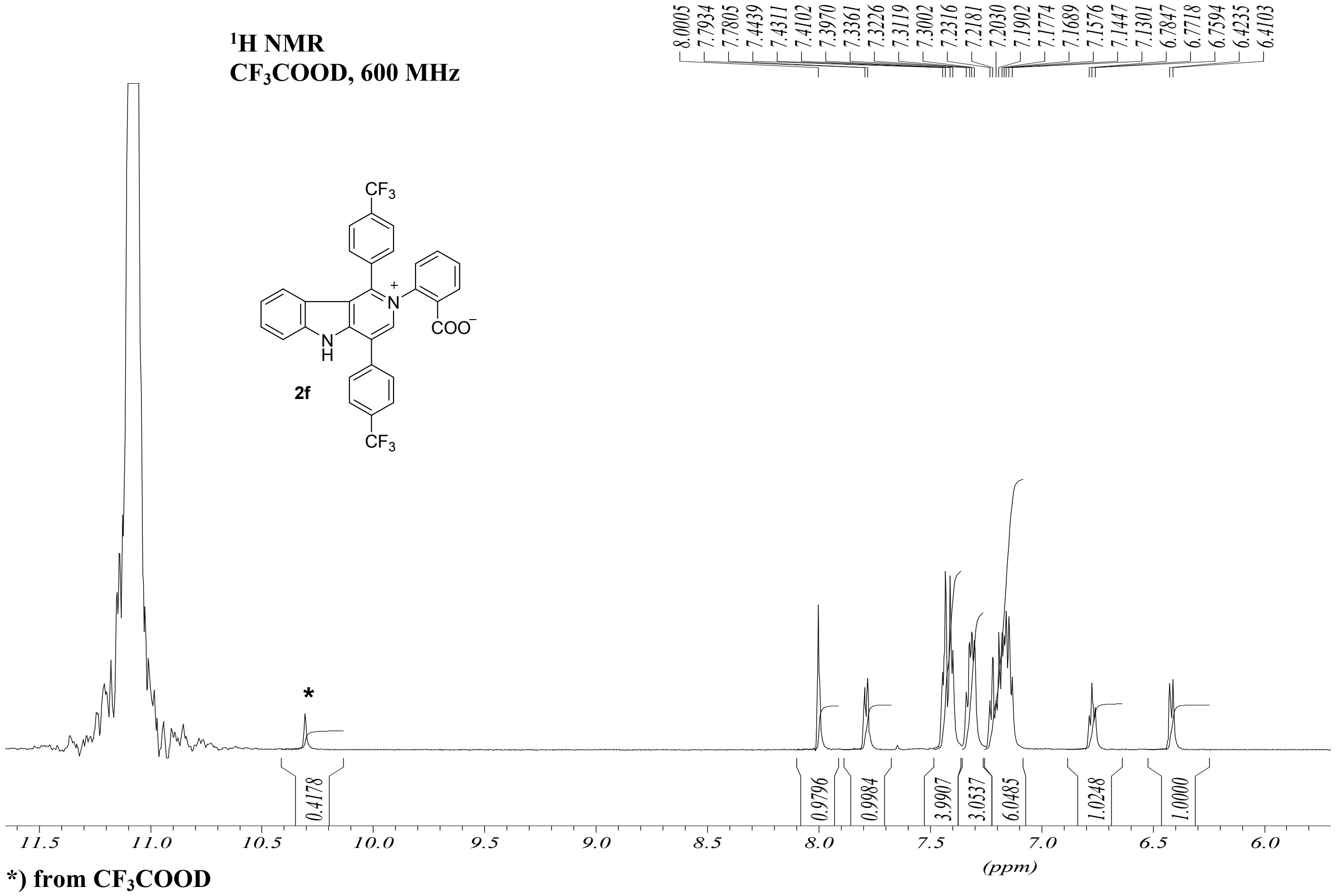

*) from CF$_3$COOD

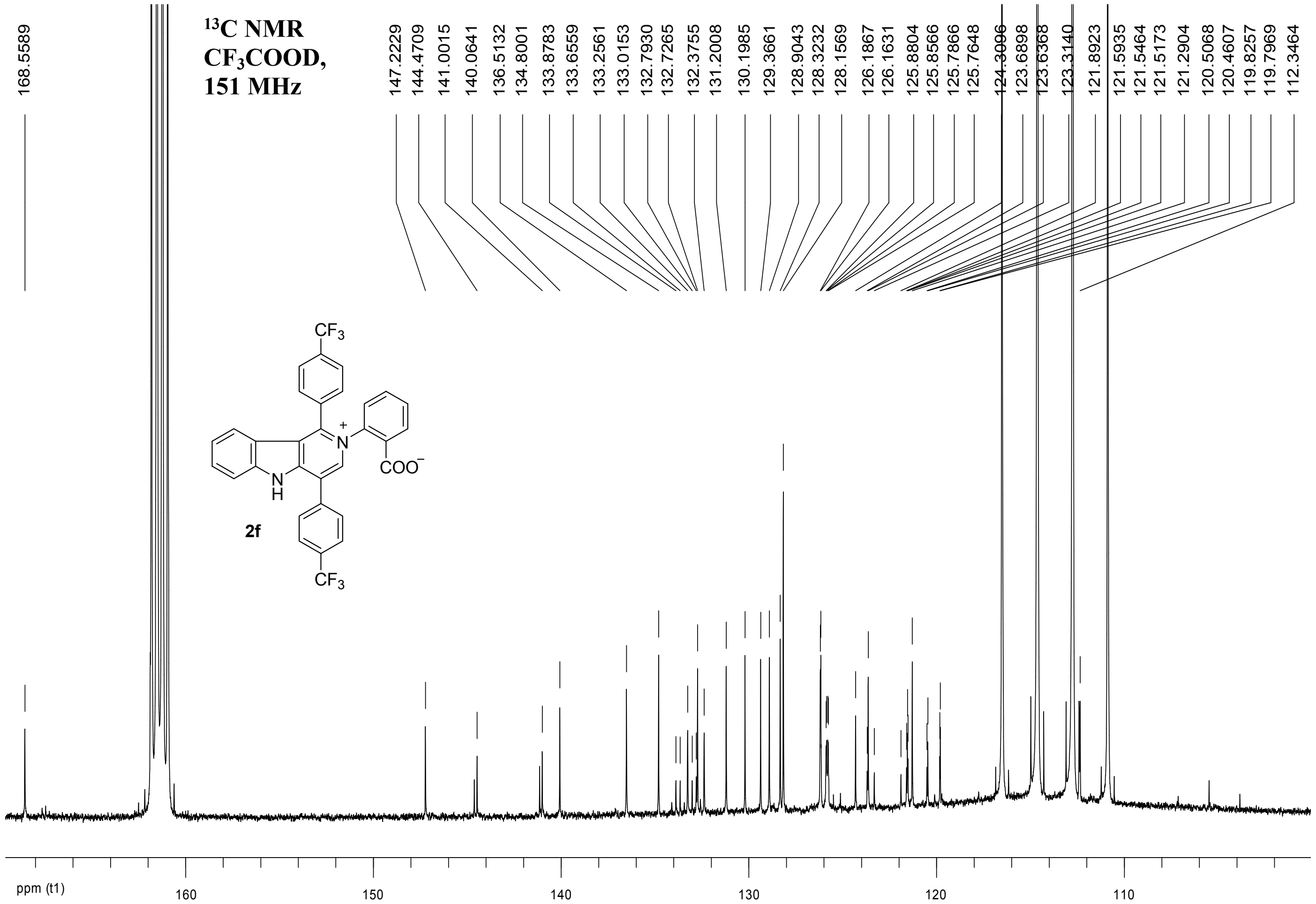
13C NMR
CF3COOD,
151 MHz
168.5589
147.2229
144.4709
141.0015
140.0641
136.5132
134.8001
133.8783
133.6559
133.2561
133.0153
132.7930
132.7265
132.3755
131.2008
130.1985
129.3661
128.9043
128.3232
128.1569
126.1867
126.1631
125.8804
125.8566
125.7866
125.7648
124.3096
123.6898
123.6368
123.3140
121.8923
121.5935
121.5464
121.5173
121.2904
120.5068
120.4607
119.8257
119.7969
112.3464
CF3
N+
COO−
N
H
2f
CF3
ppm (t1)
160
150
140
130
120
110

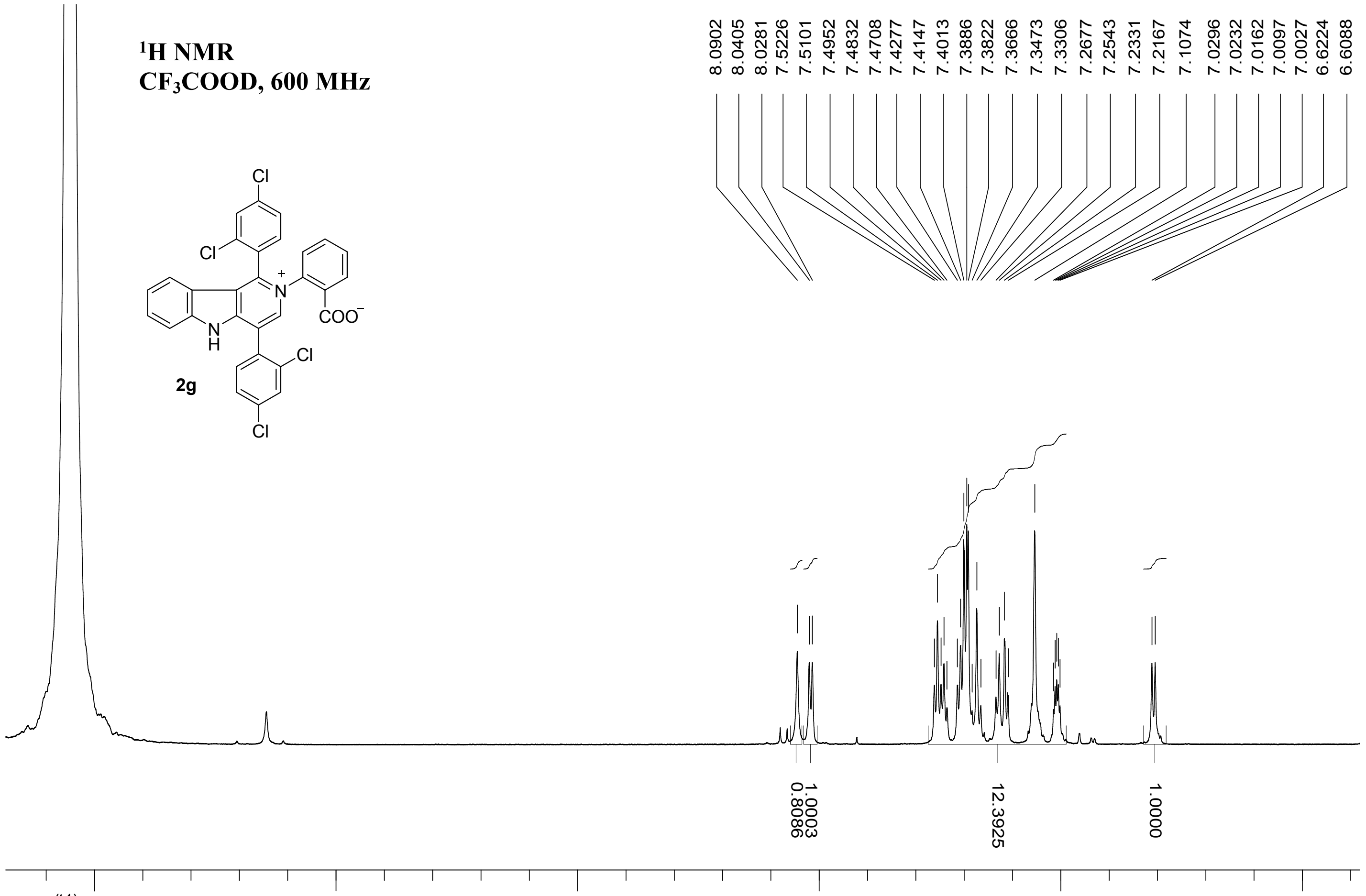
1H NMR
CF3COOD, 600 MHz
8.0902
8.0405
8.0281
7.5226
7.5101
7.4952
7.4832
7.4708
7.4277
7.4147
7.4013
7.3886
7.3822
7.3666
7.3473
7.3306
7.2677
7.2543
7.2331
7.2167
7.1074
7.0296
7.0232
7.0162
7.0097
7.0027
6.6224
6.6088
Cl
Cl
N+
COO−
N
H
Cl
2g
Cl
0.8086
1.0003
12.3925
1.0000
ppm (t1)
11.0
10.0
9.0
8.0
7.0
6.0

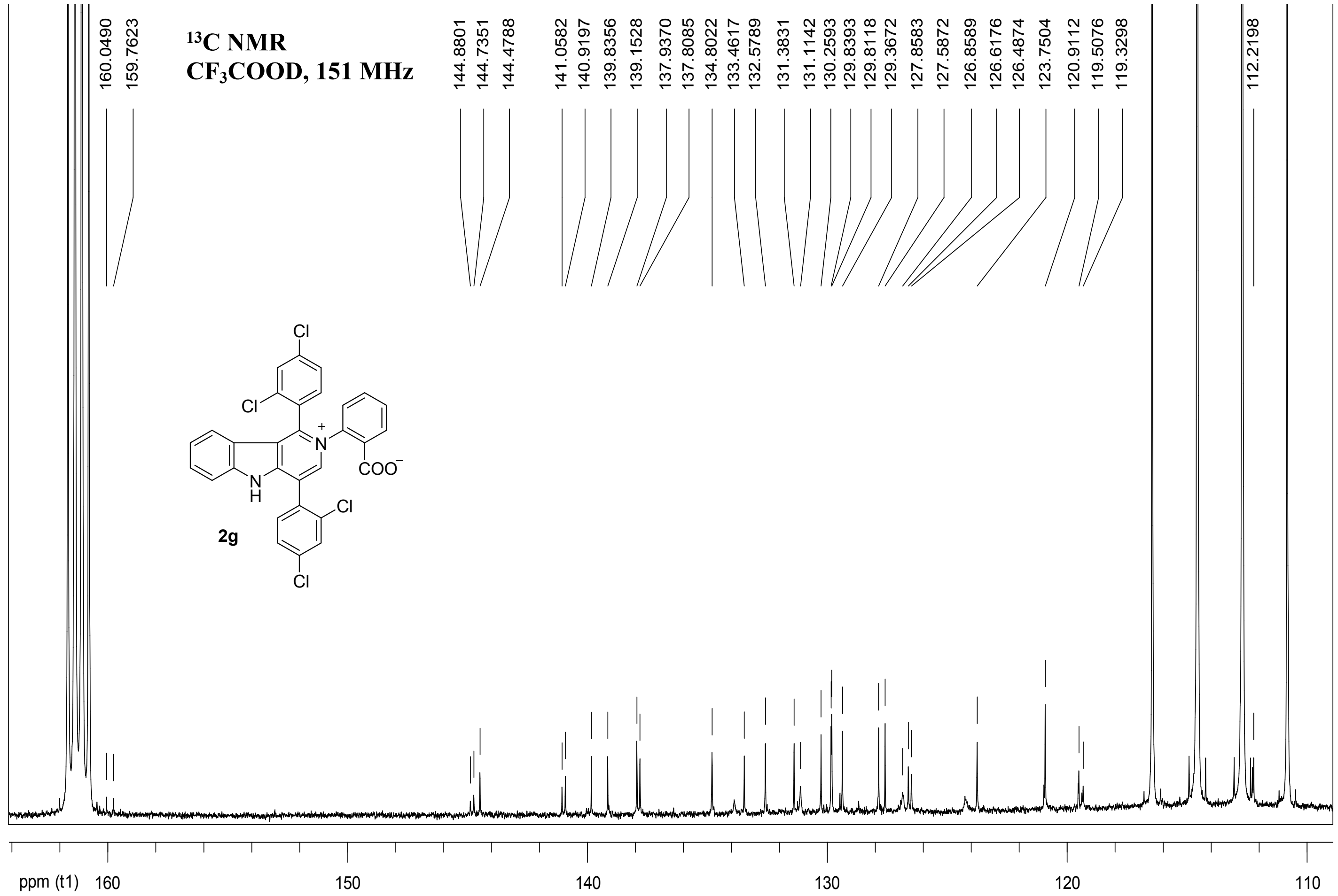

13C NMR
CF3COOD, 151 MHz
160.0490
159.7623
144.8801
144.7351
144.4788
141.0582
140.9197
139.8356
139.1528
137.9370
137.8085
134.8022
133.4617
132.5789
131.3831
131.1142
130.2593
129.8393
129.8118
129.3672
127.8583
127.5872
126.8589
126.6176
126.4874
123.7504
120.9112
119.5076
119.3298
112.2198
Cl
Cl
N
COO
N
H
Cl
Cl
2g
ppm (t1)
160
150
140
130
120
110

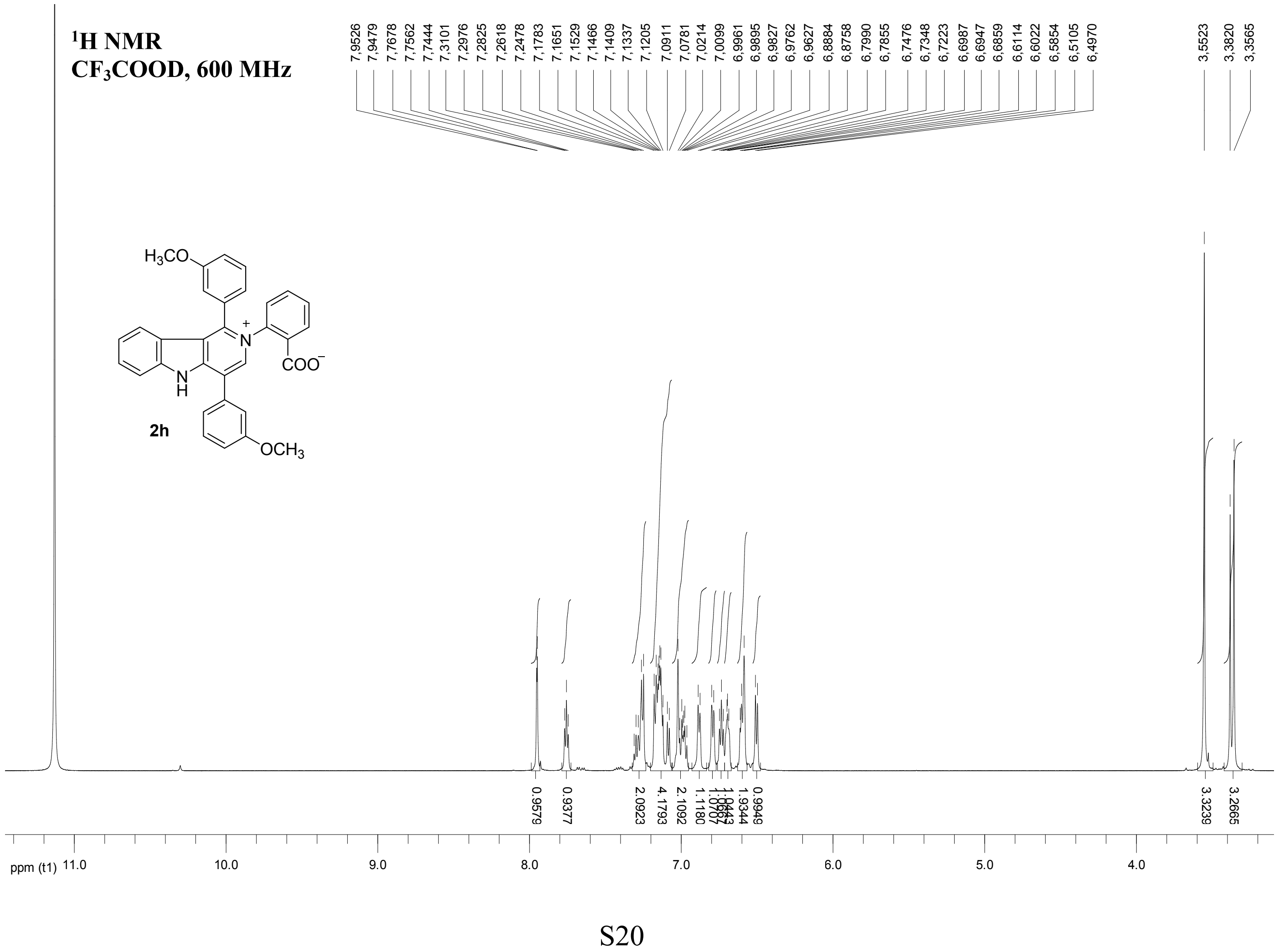

1H NMR
CF3COOD, 600 MHz
7,9526
7,9479
7,7678
7,7562
7,7444
7,3101
7,2976
7,2825
7,2618
7,2478
7,1783
7,1651
7,1529
7,1466
7,1409
7,1337
7,1205
7,0911
7,0781
7,0214
7,0099
6,9961
6,9895
6,9827
6,9762
6,9627
6,8884
6,8758
6,7990
6,7855
6,7476
6,7348
6,7223
6,6987
6,6947
6,6859
6,6114
6,6022
6,5854
6,5105
6,4970
3,5523
3,3820
3,3565
H3CO
N
COO
N
H
OCH3
2h
0.9579
0.9377
2.0923
4.1793
2.1092
1.1180
1.9344
0.9949
3.3239
3.2665
ppm (t1)
11.0
10.0
9.0
8.0
7.0
6.0
5.0
4.0

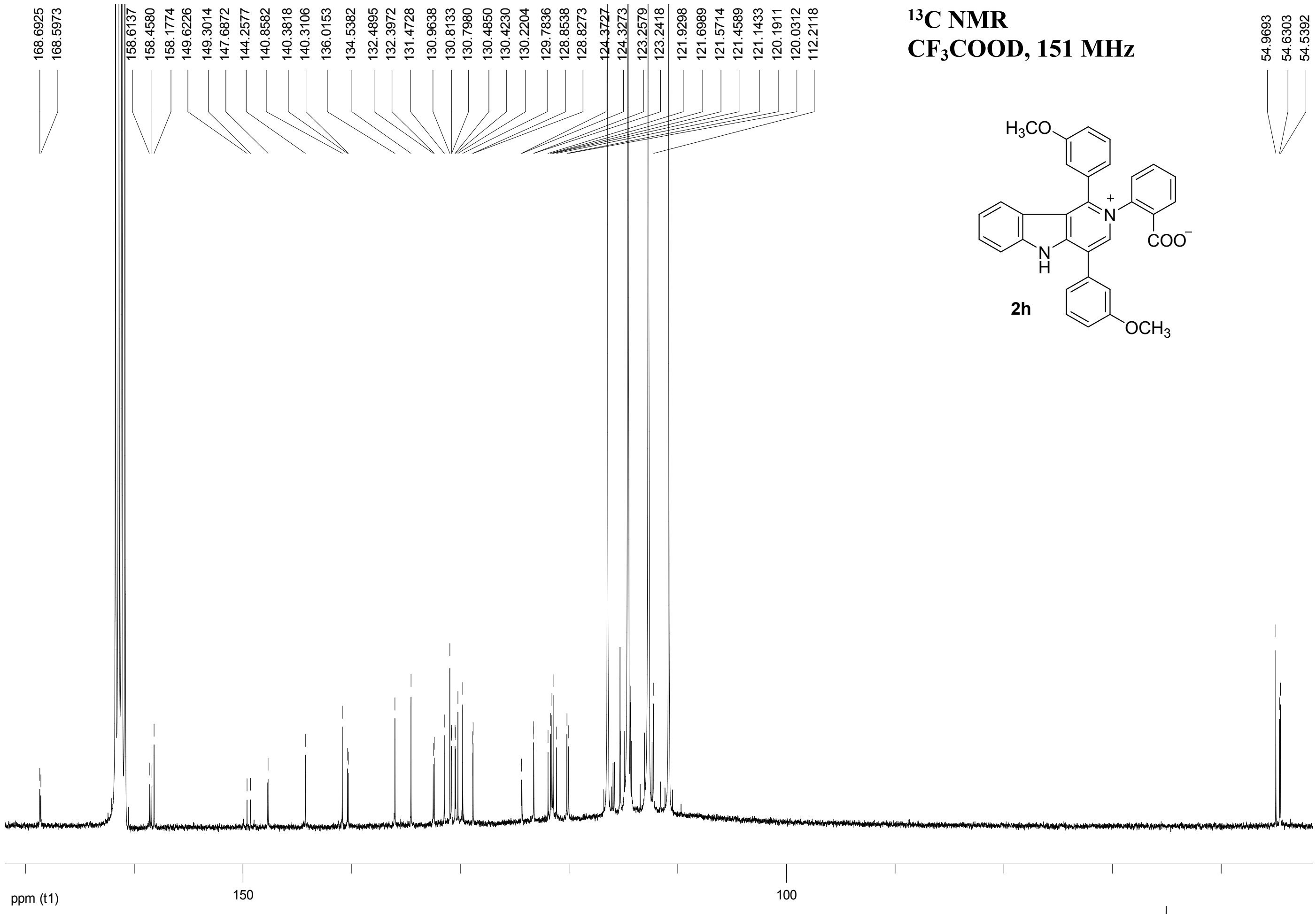
168.6925
168.5973
158.6137
158.4580
158.1774
149.6226
149.3014
147.6872
144.2577
140.8582
140.3818
140.3106
136.0153
134.5382
132.4895
132.3972
131.4728
130.9638
130.8133
130.7980
130.4850
130.4230
130.2204
129.7836
128.8538
128.8273
124.3727
124.3273
123.2579
123.2418
121.9298
121.6989
121.5714
121.4589
121.1433
120.1911
120.0312
112.2118
54.9693
54.6303
54.5392
13C NMR
CF3COOD, 151 MHz
H3CO
N
H
COO−
2h
OCH3
ppm (t1)
150
100

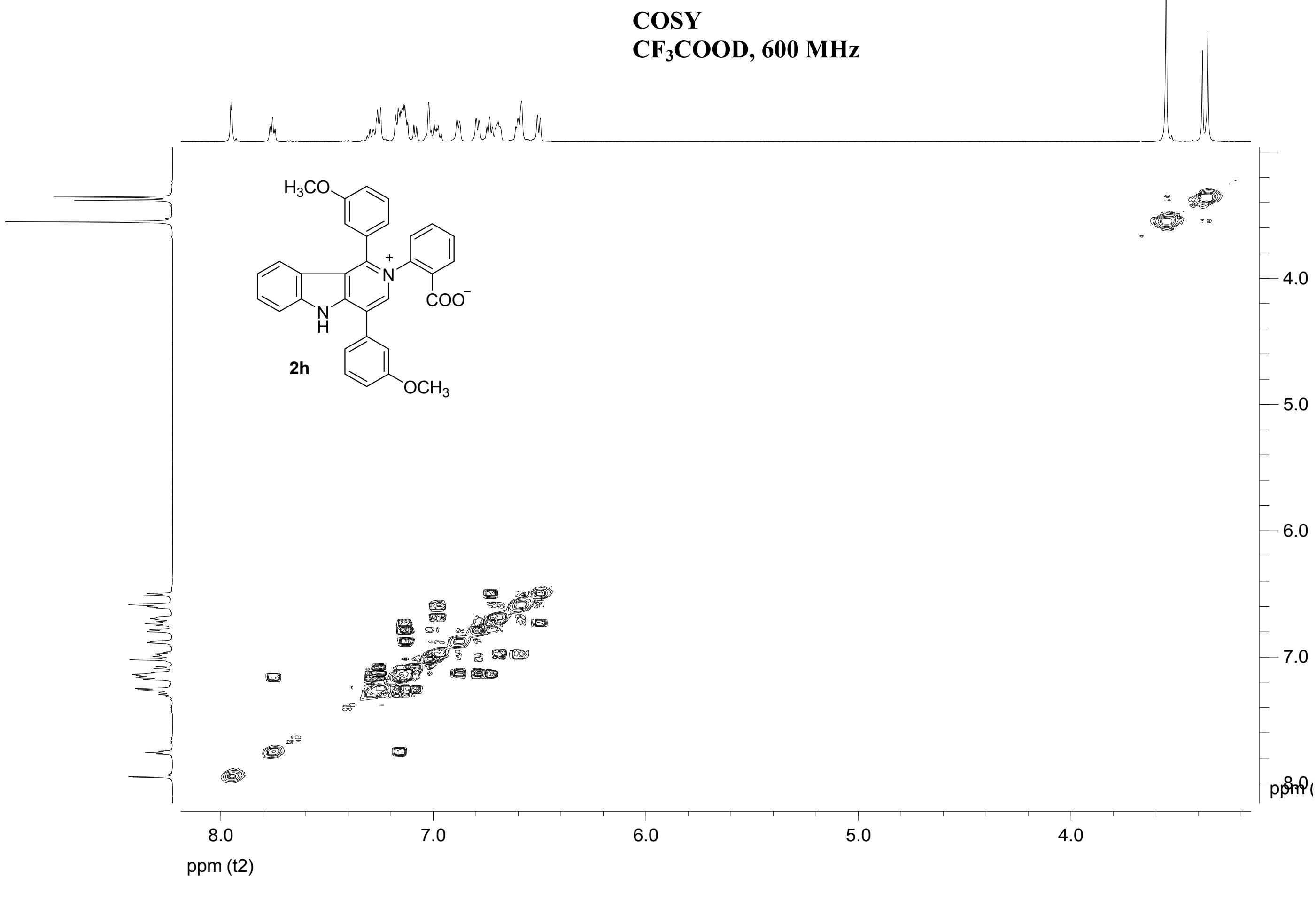
COSY
CF3COOD, 600 MHz
H3CO
N
H
COO−
2h
OCH3
4.0
5.0
6.0
7.0
8.0
ppm
8.0
7.0
6.0
5.0
4.0
ppm (t2)

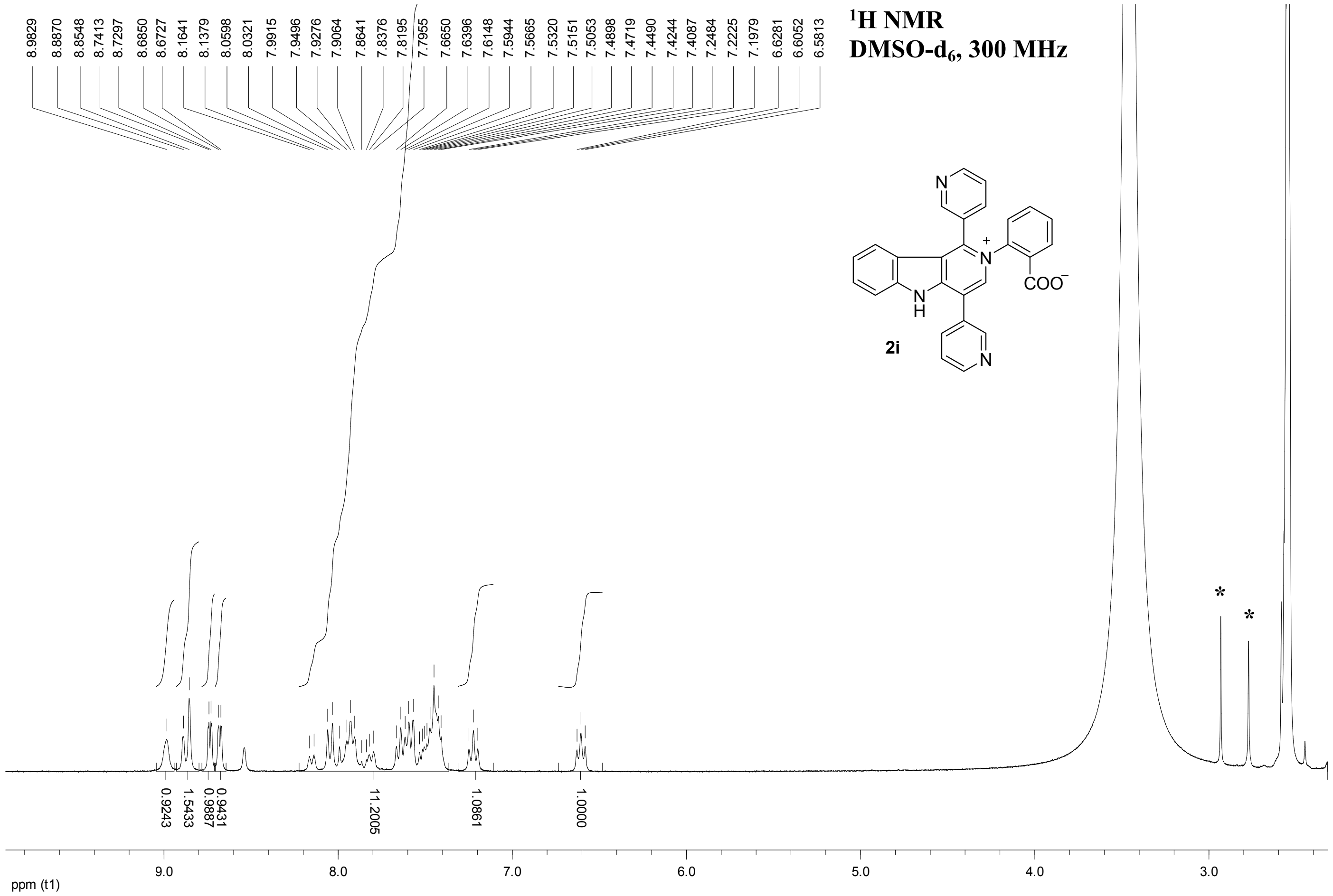

***) signals from DMFA**

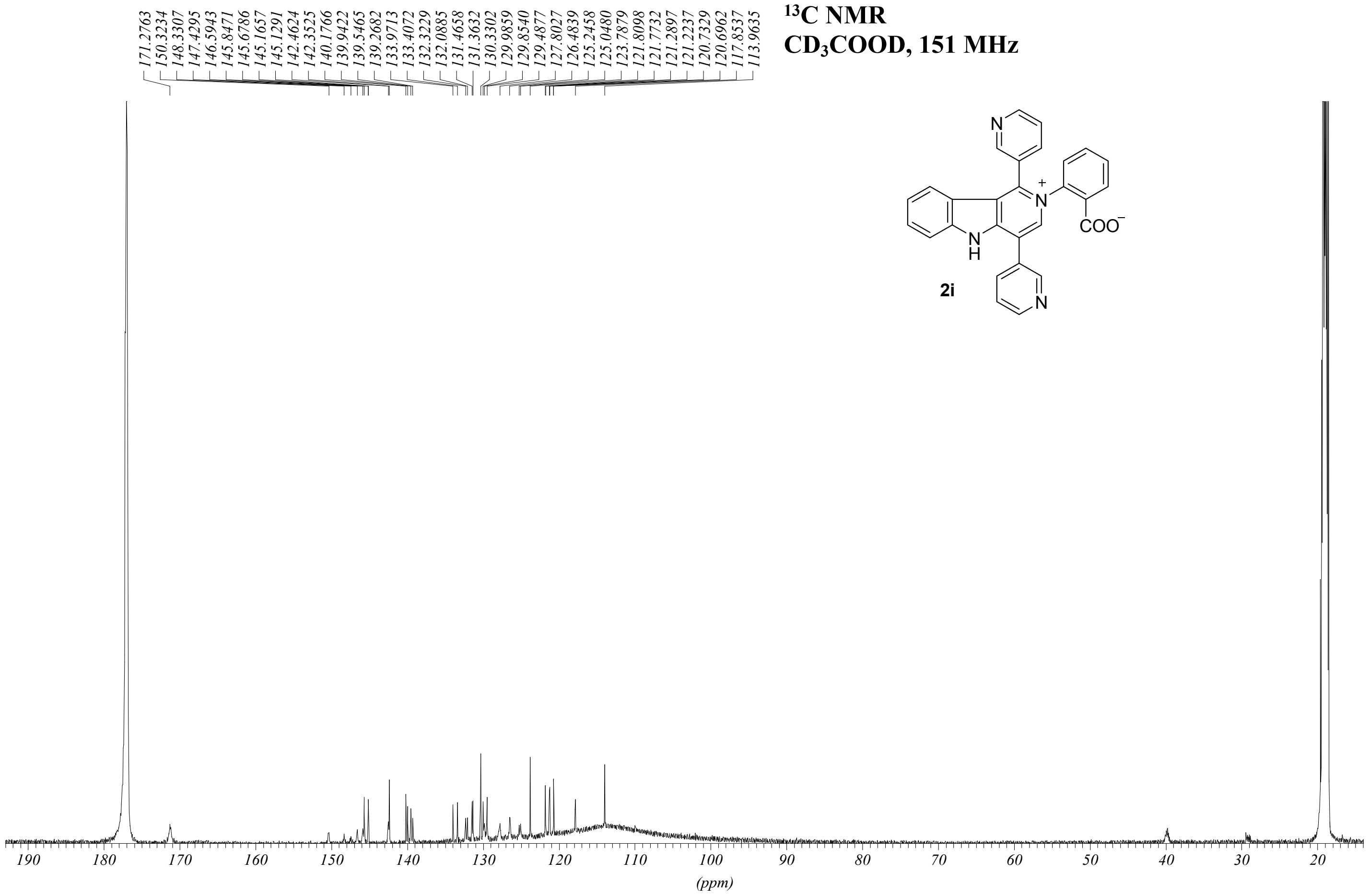

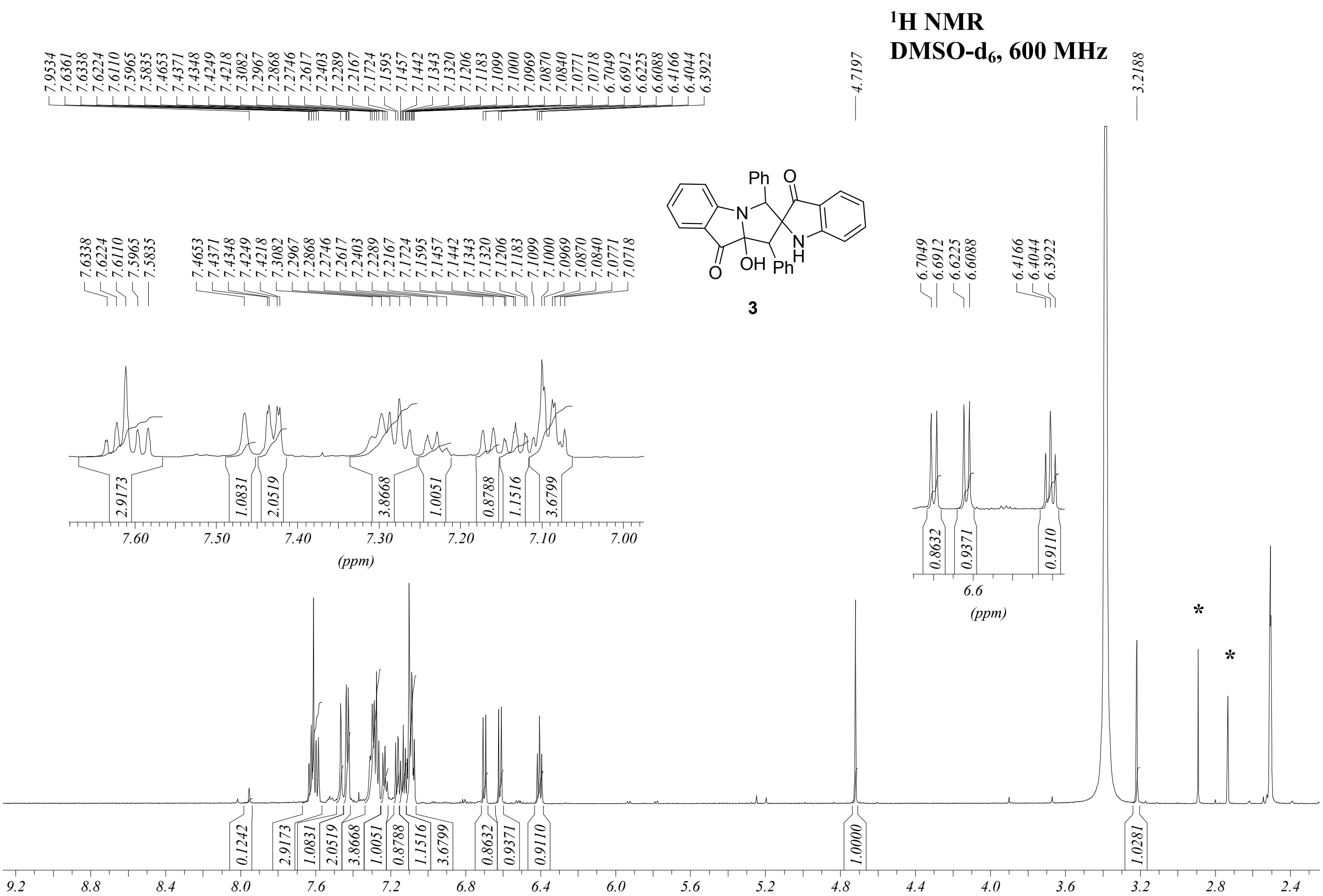

*) signals from DMFA

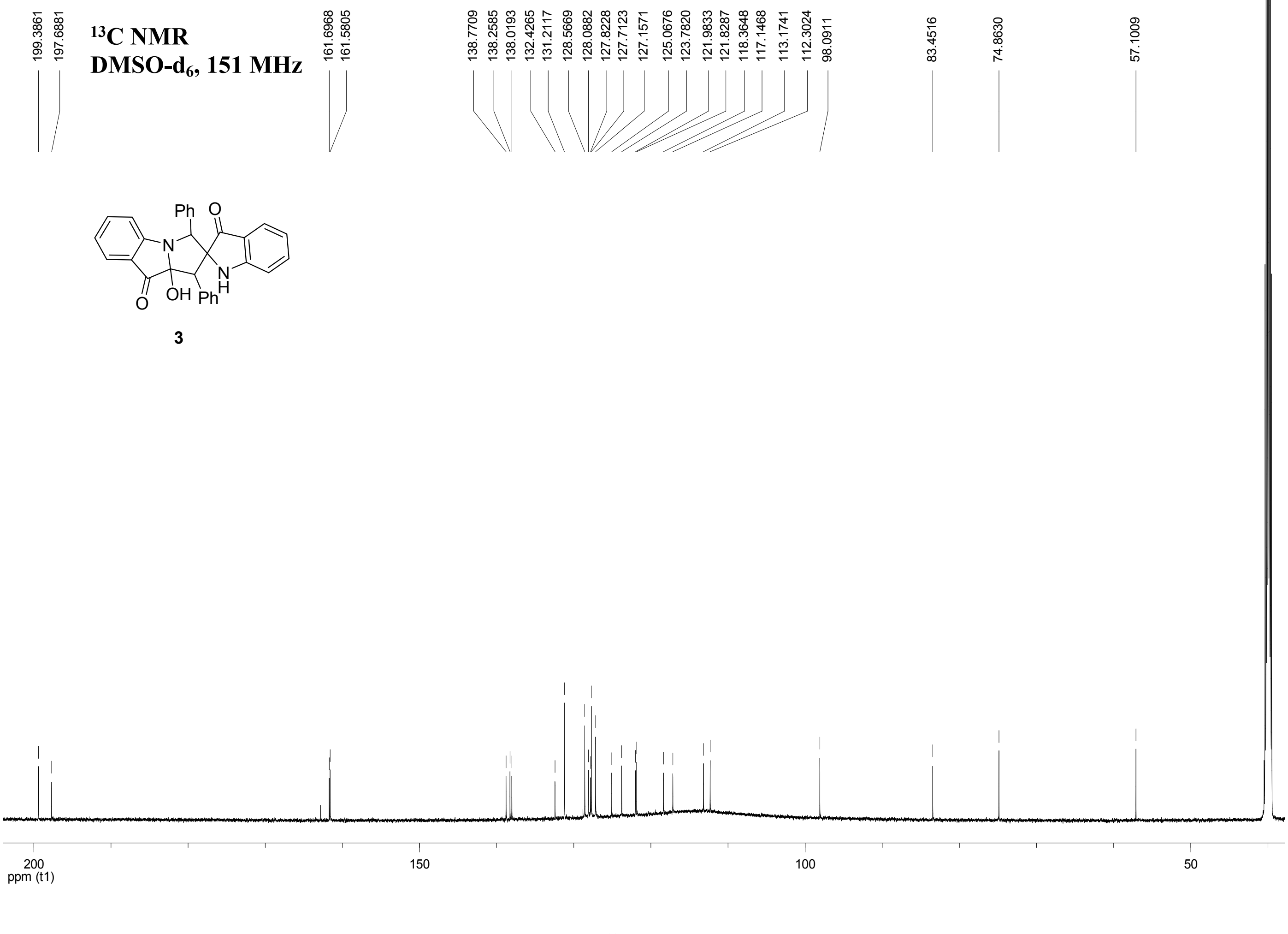

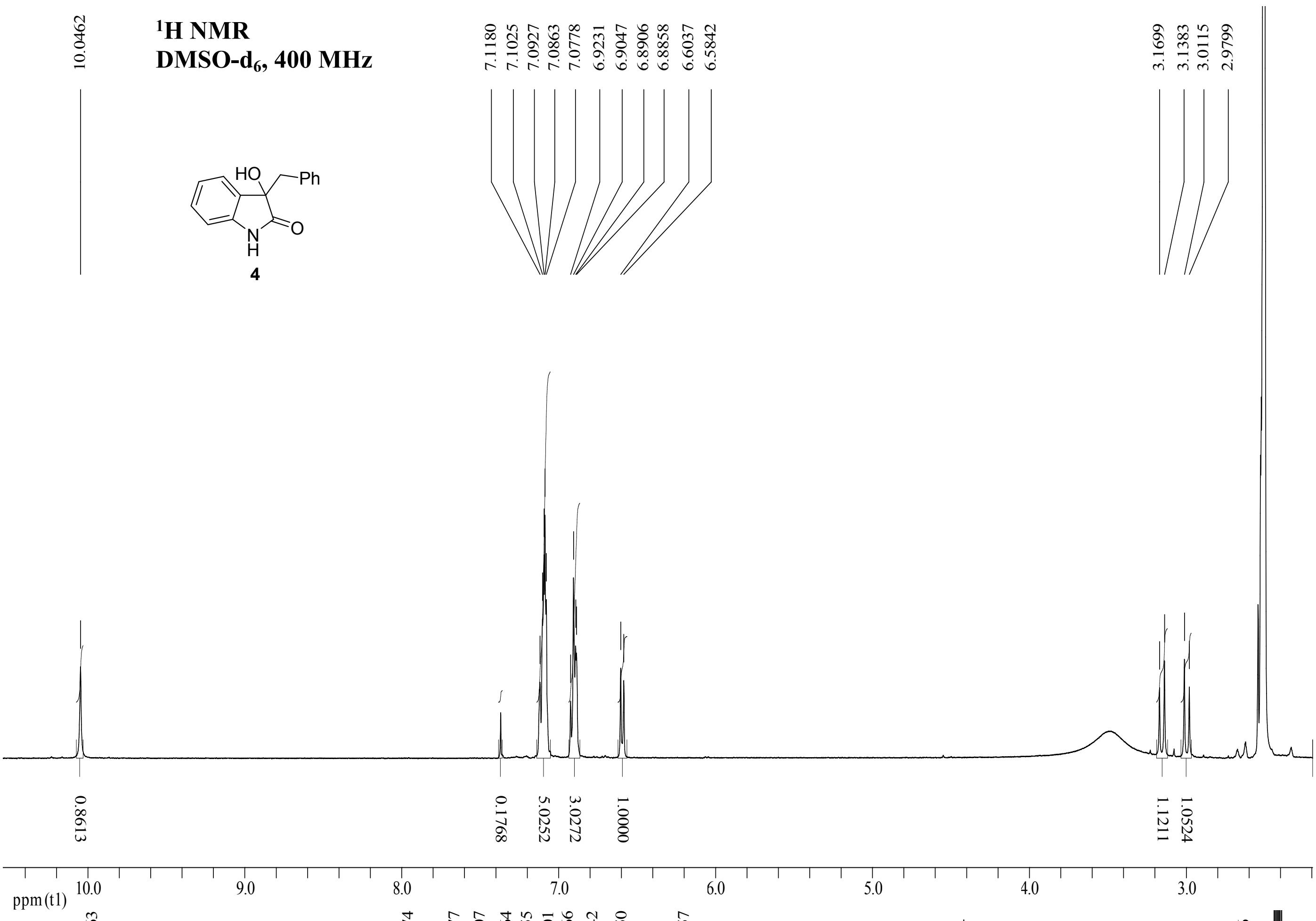

1H NMR
DMSO-d6, 400 MHz
HO
Ph
N
H
O
4
10.0462
7.1180
7.1025
7.0927
7.0863
7.0778
6.9231
6.9047
6.8906
6.8858
6.6037
6.5842
3.1699
3.1383
3.0115
2.9799
0.8613
0.1768
5.0252
3.0272
1.0000
1.1211
1.0524
ppm (t1)
10.0
9.0
8.0
7.0
6.0
5.0
4.0
3.0

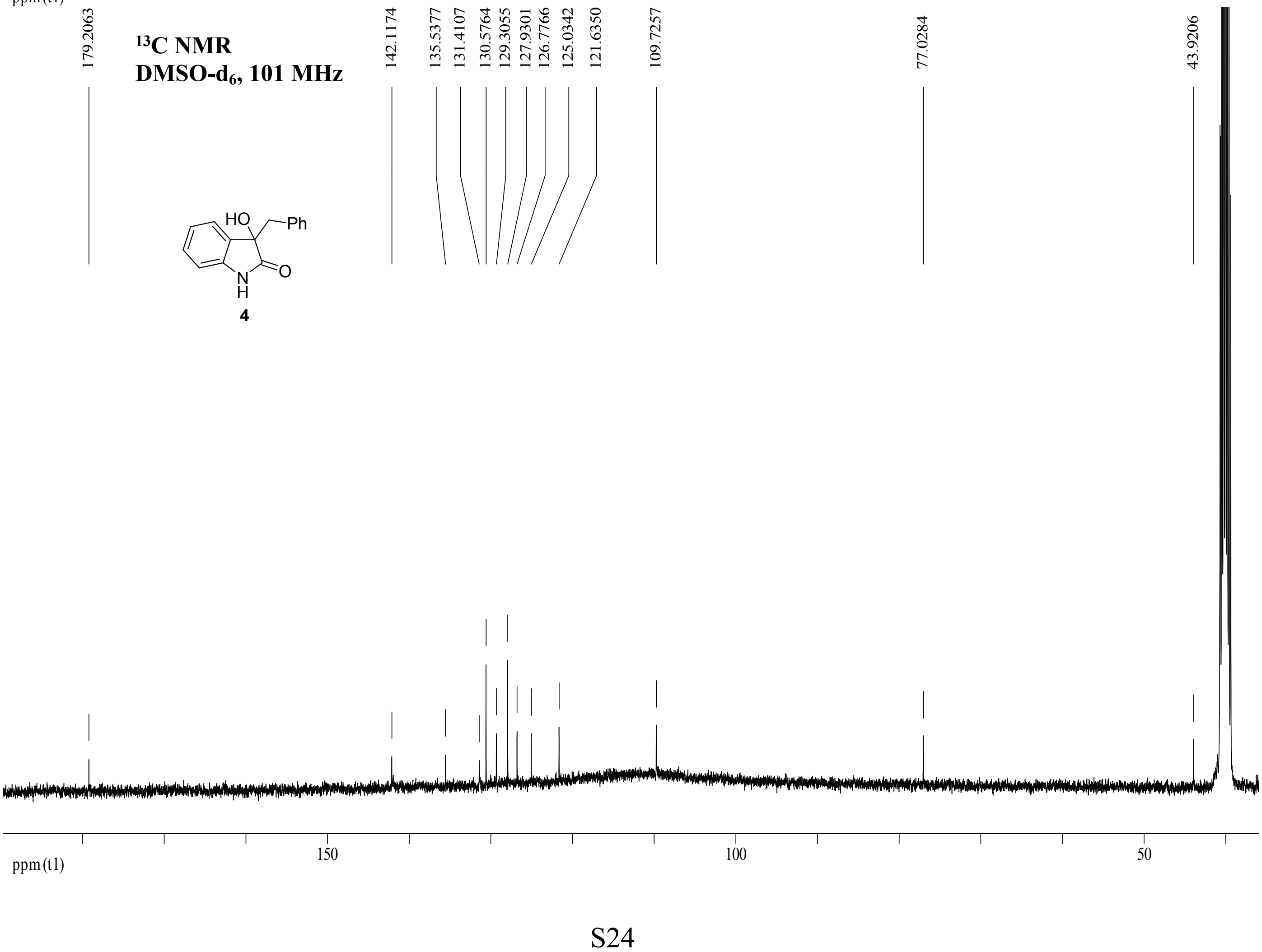

13C NMR
DMSO-d6, 101 MHz
HO
Ph
N
H
O
4
179.2063
142.1174
135.5377
131.4107
130.5764
129.3055
127.9301
126.7766
125.0342
121.6350
109.7257
77.0284
43.9206
ppm (t1)
150
100
50

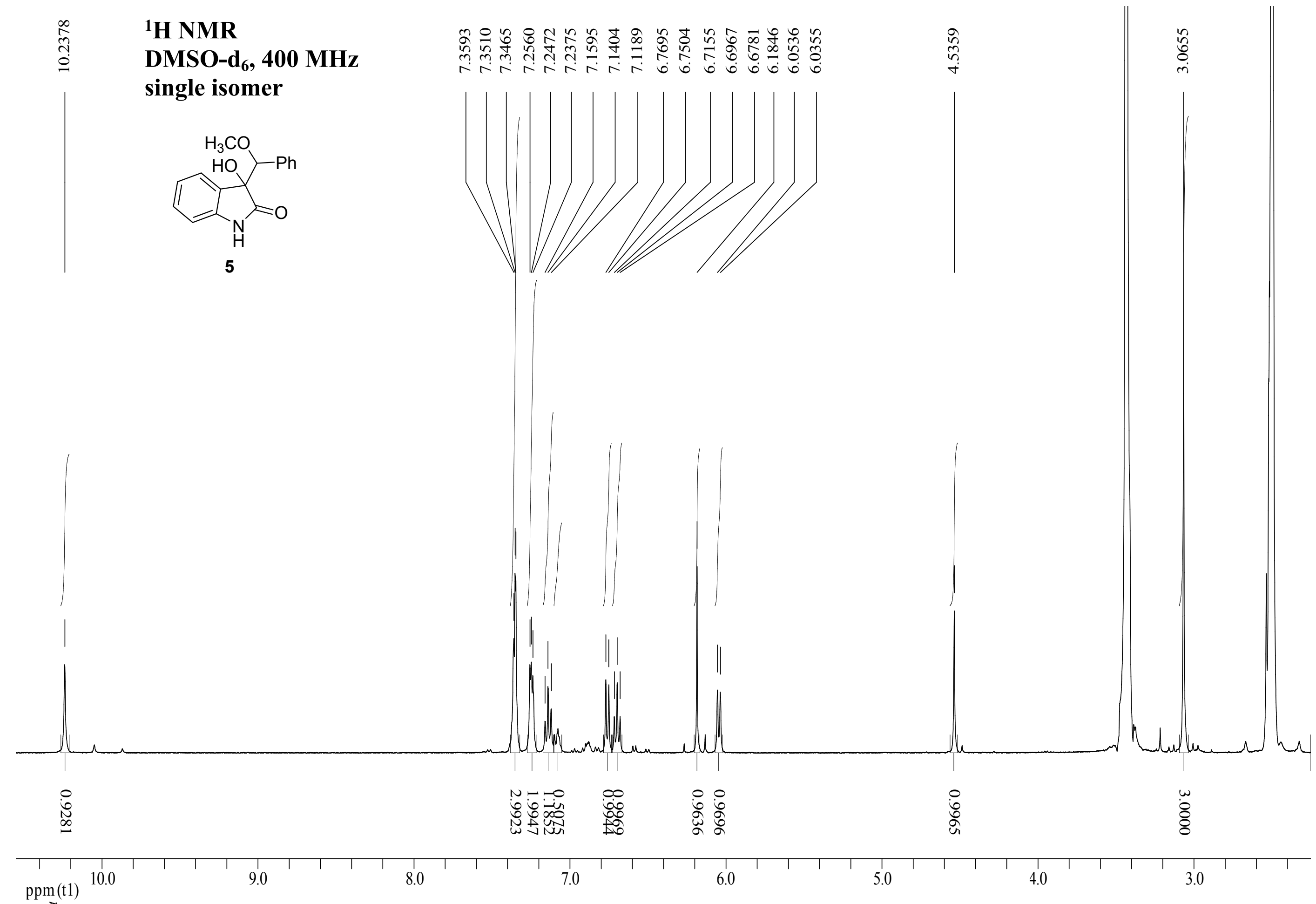

^{1}H NMR
DMSO-d_6, 400 MHz
single isomer
H_3CO
HO
Ph
O
N
H
5
ppm (t1)

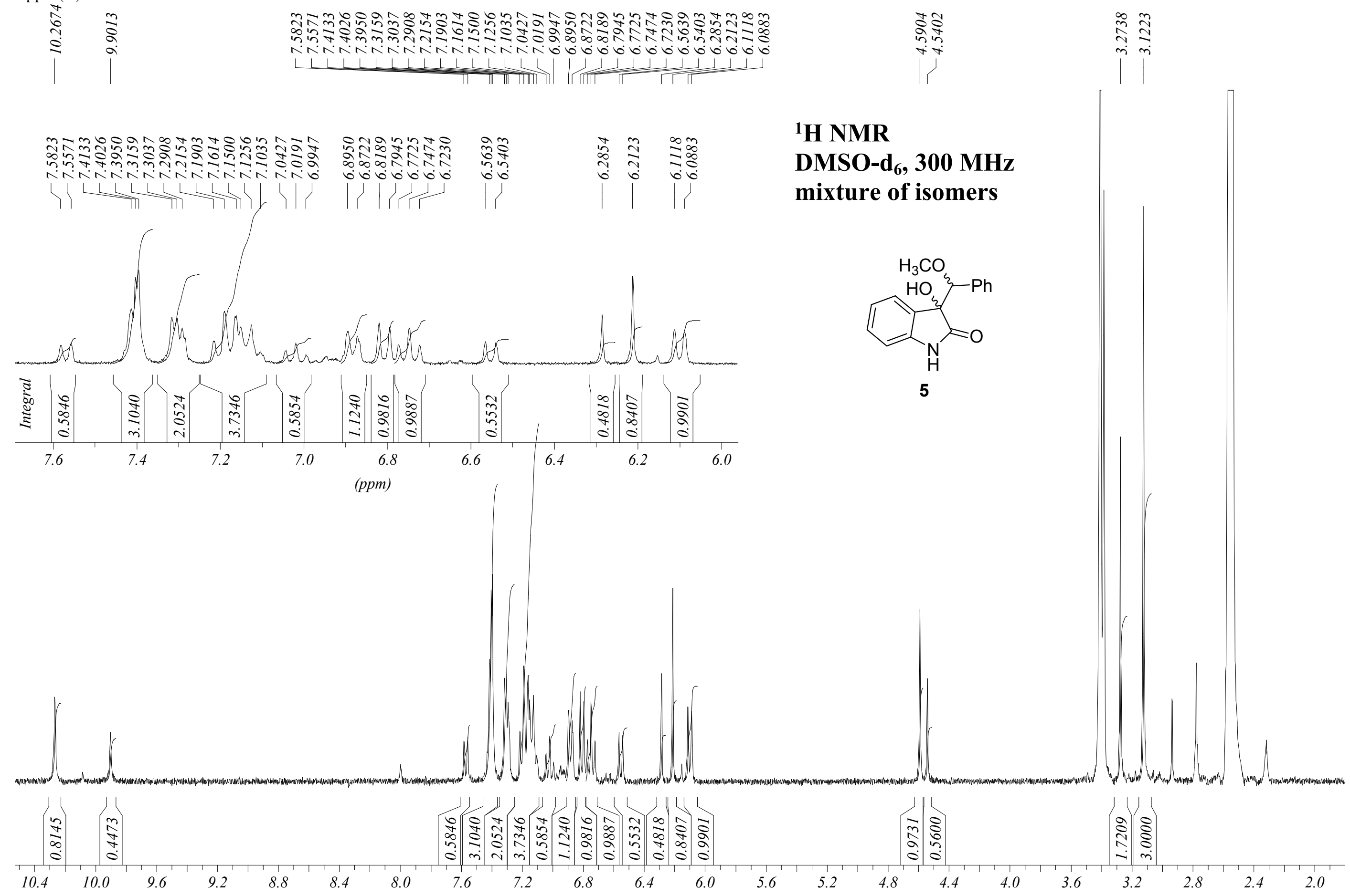

^{1}H NMR
DMSO-d_6, 300 MHz
mixture of isomers
H_3CO
HO
Ph
O
N
H
5
Integral
(ppm)

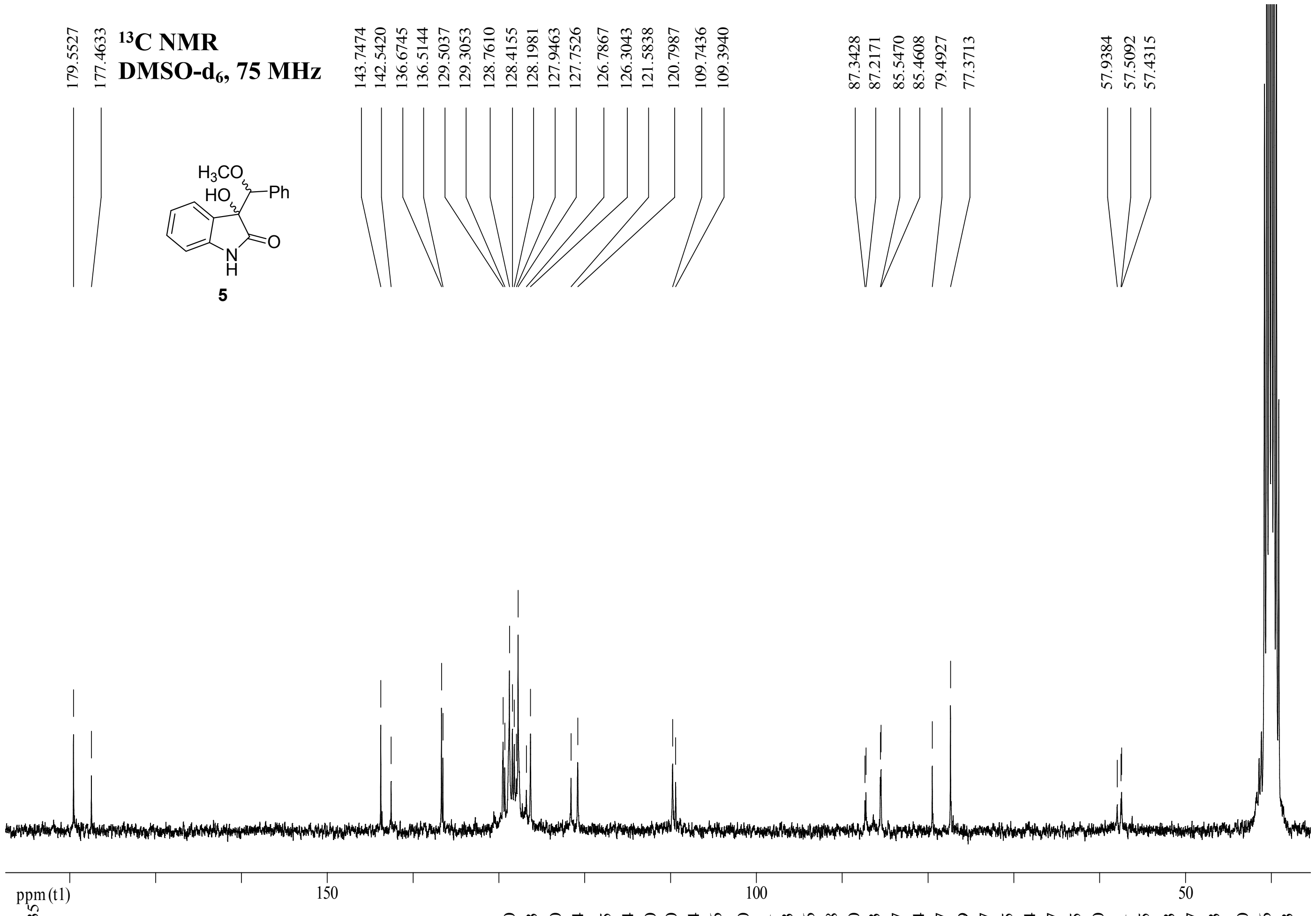

13C NMR
DMSO-d6, 75 MHz
179.5527
177.4633
143.7474
142.5420
136.6745
136.5144
129.5037
129.3053
128.7610
128.4155
128.1981
127.9463
127.7526
126.7867
126.3043
121.5838
120.7987
109.7436
109.3940
87.3428
87.2171
85.5470
85.4608
79.4927
77.3713
57.9384
57.5092
57.4315
H3CO
HO
Ph
O
N
H
5
ppm (t1)
150
100
50

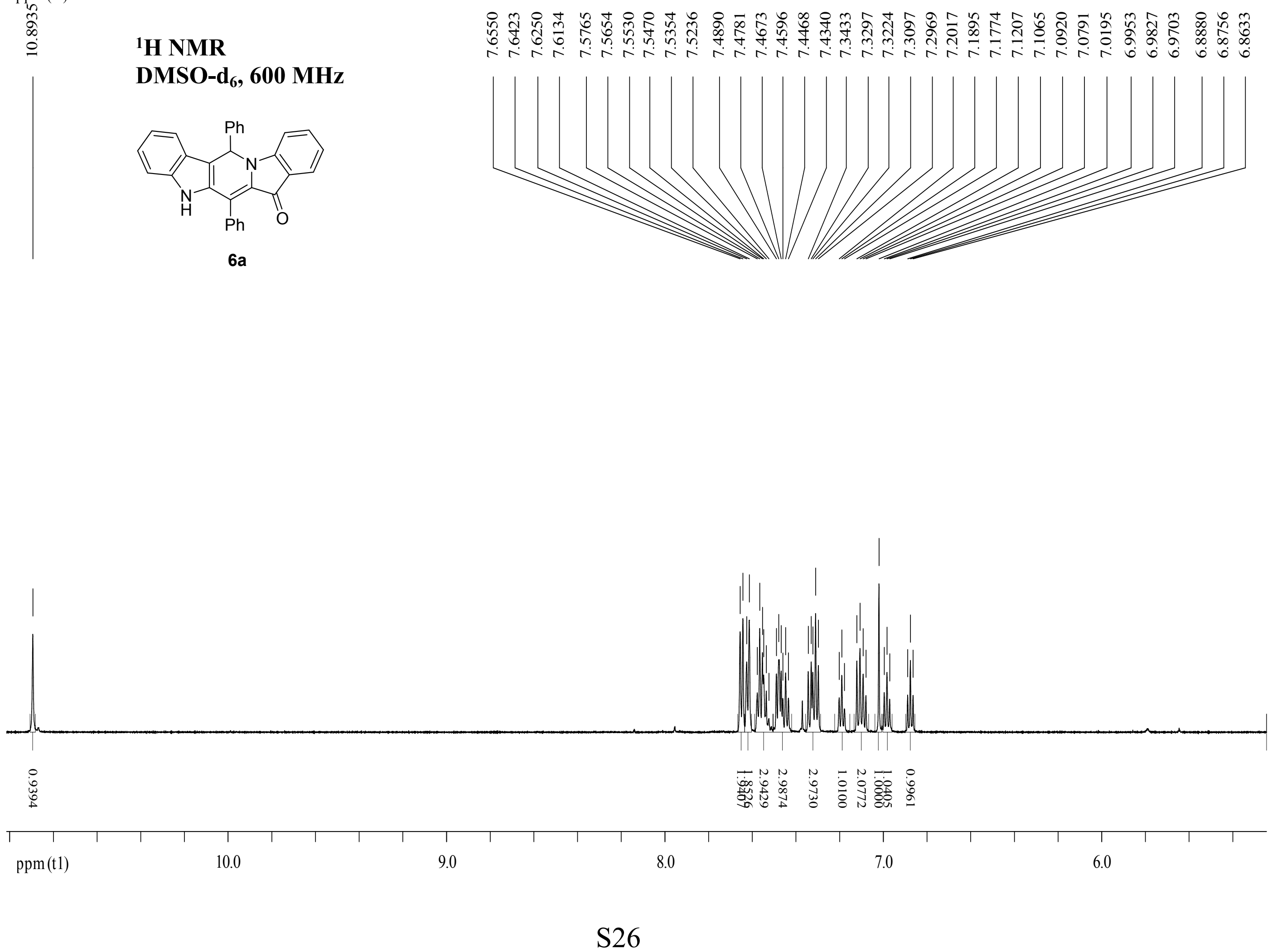

1H NMR
DMSO-d6, 600 MHz
10.8935
7.6550
7.6423
7.6250
7.6134
7.5765
7.5654
7.5530
7.5470
7.5354
7.5236
7.4890
7.4781
7.4673
7.4596
7.4468
7.4340
7.3433
7.3297
7.3224
7.3097
7.2969
7.2017
7.1895
7.1774
7.1207
7.1065
7.0920
7.0791
7.0195
6.9953
6.9827
6.9703
6.8880
6.8756
6.8633
Ph
N
N
H
Ph
O
6a
0.9394
2.9429
2.9874
2.9730
1.0100
2.0772
0.9961
ppm (t1)
10.0
9.0
8.0
7.0
6.0

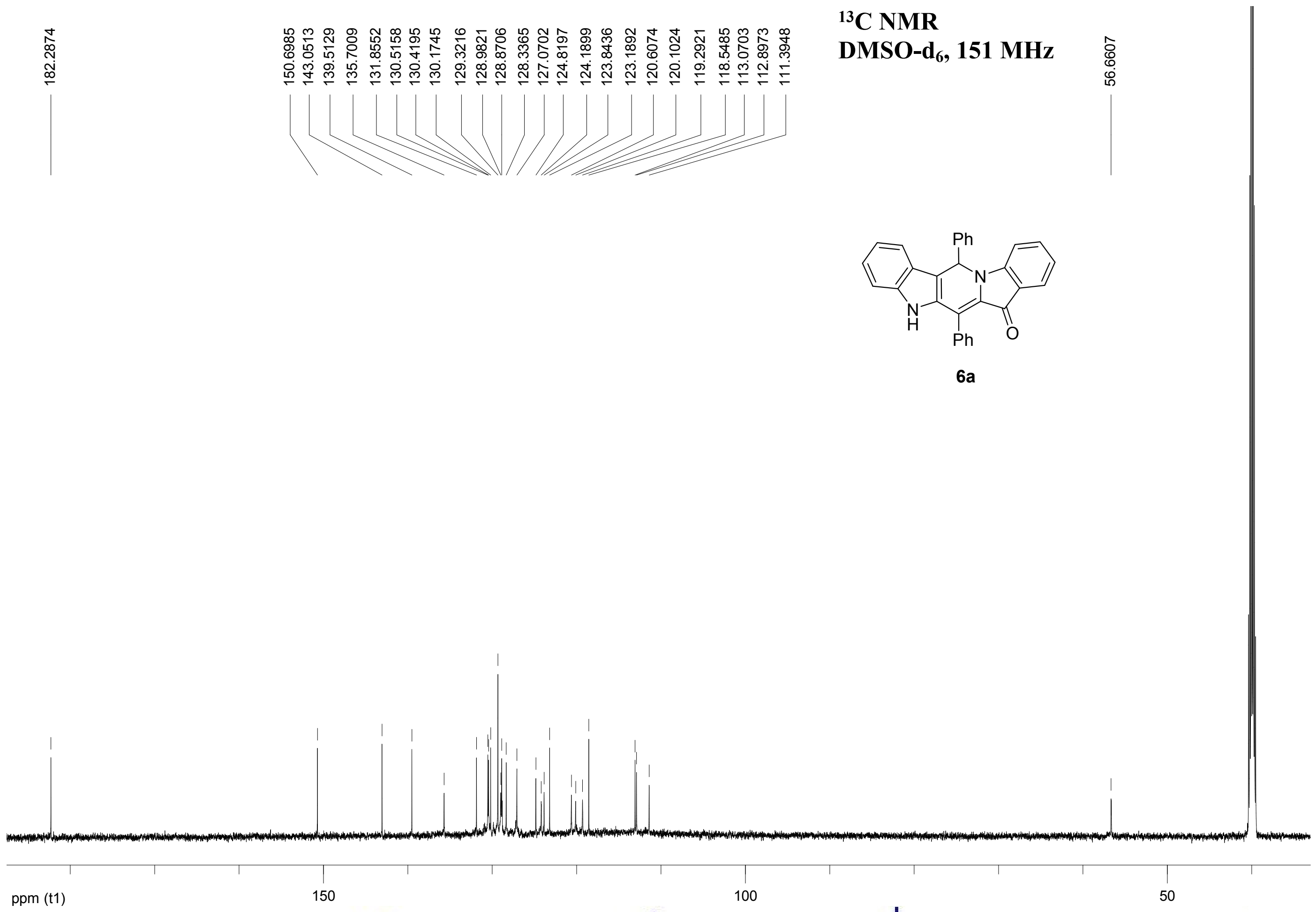
182.2874
150.6985
143.0513
139.5129
135.7009
131.8552
130.5158
130.4195
130.1745
129.3216
128.9821
128.8706
128.3365
127.0702
124.8197
124.1899
123.8436
123.1892
120.6074
120.1024
119.2921
118.5485
113.0703
112.8973
111.3948
^{13}C NMR
DMSO-d$_6$, 151 MHz
56.6607
Ph
N
NH
Ph
O
6a
ppm (t1)
150
100
50

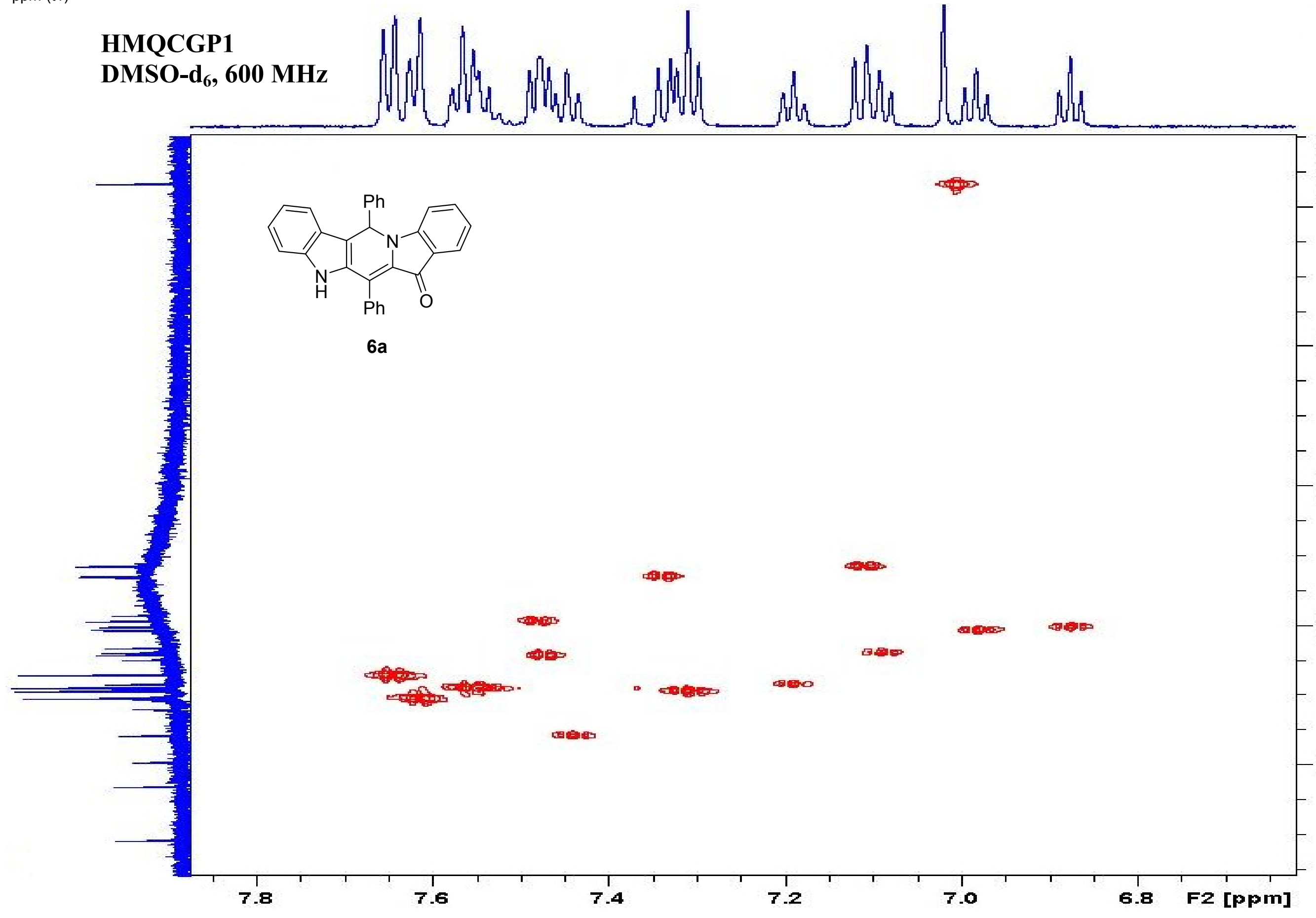
HMQCGP1
DMSO-d$_6$, 600 MHz
Ph
N
H
Ph
O
6a
F1 [ppm]
80
100
120
140
7.8
7.6
7.4
7.2
7.0
6.8
F2 [ppm]

HMQCGP2
DMSO-d$_6$, 600 MHz

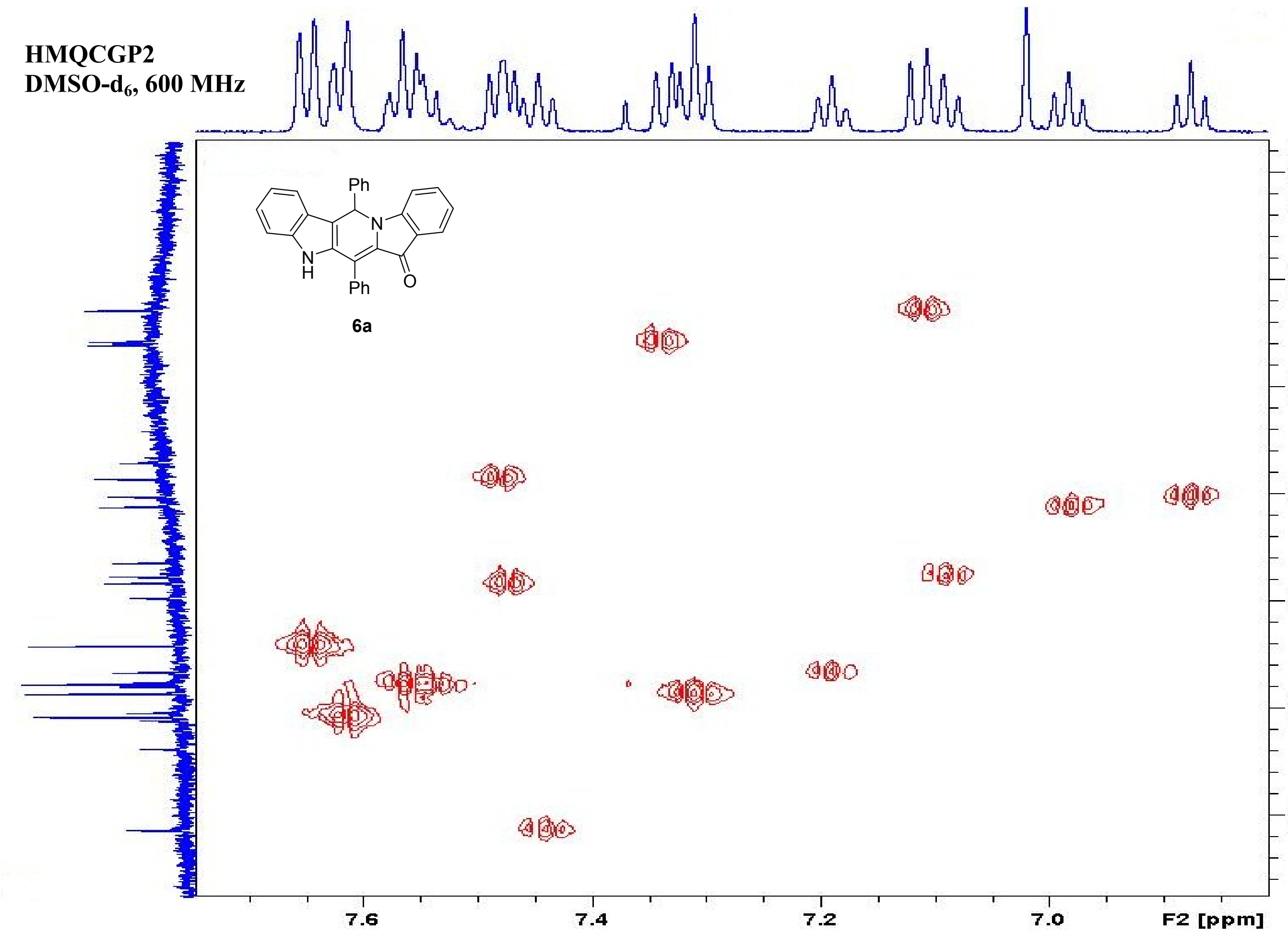

COSY
DMSO-d$_6$, 600 MHz

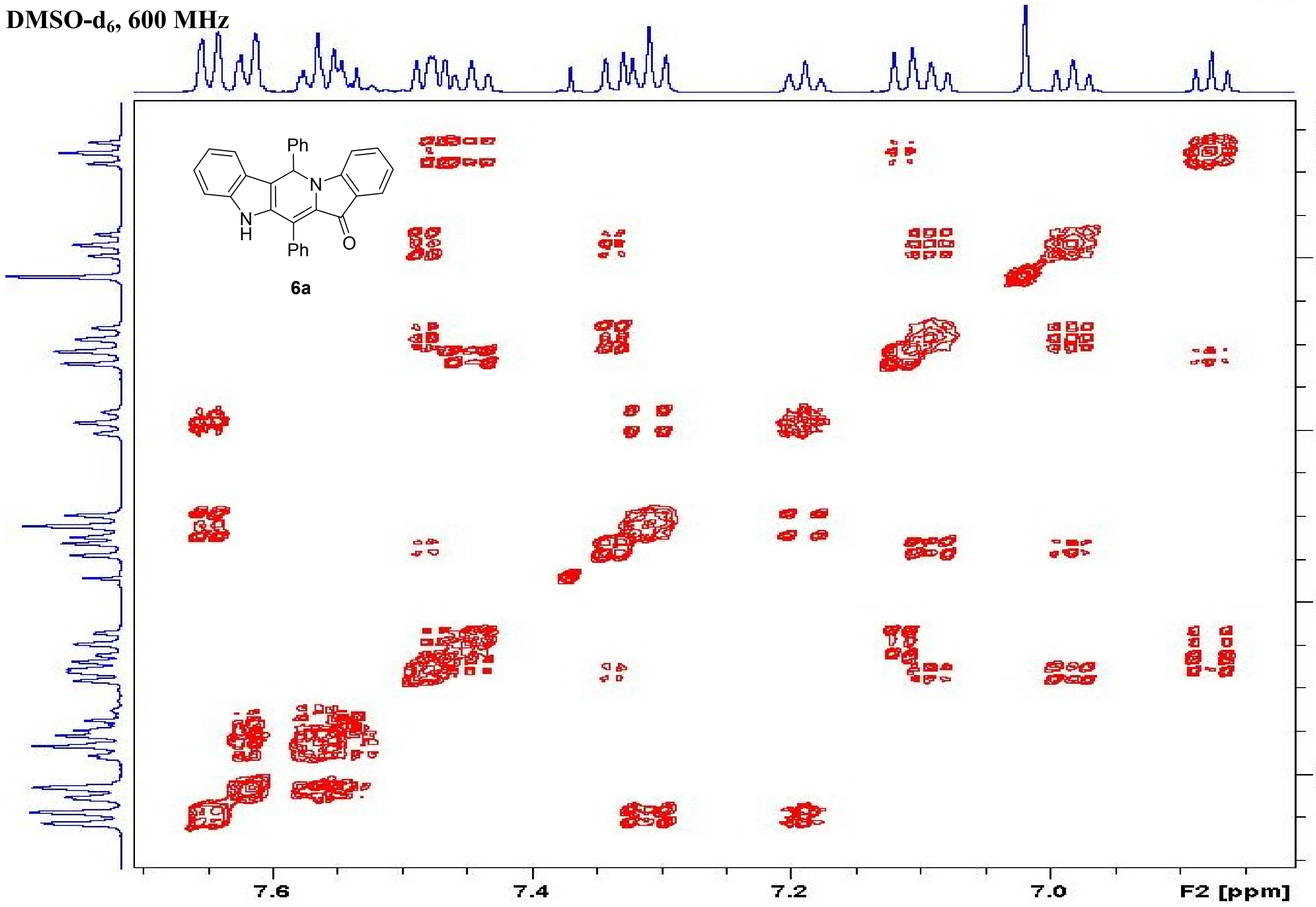

NOESY
DMSO-d_6, 600 MHz

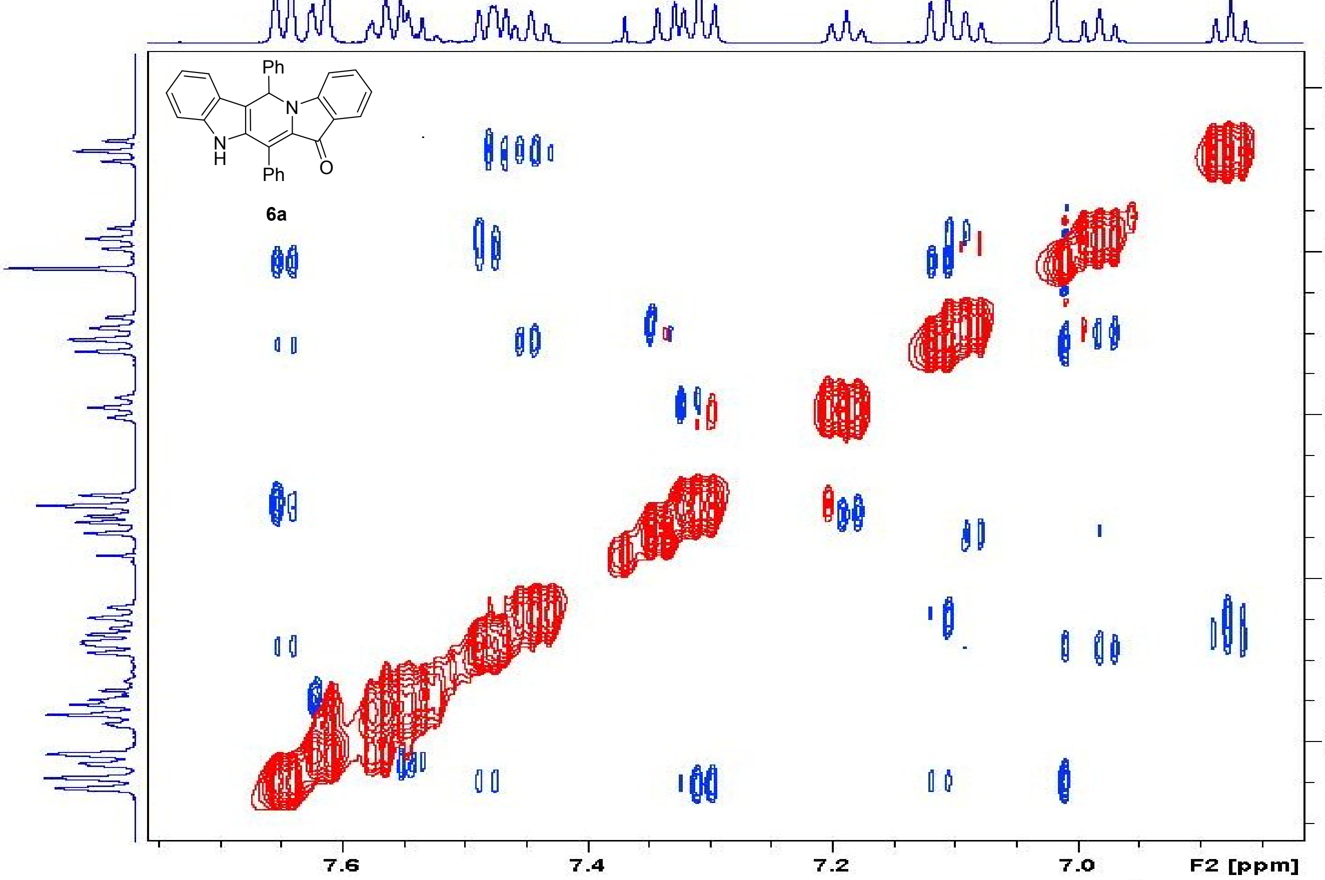

HMBCGP1
DMSO-d_6, 600 MHz

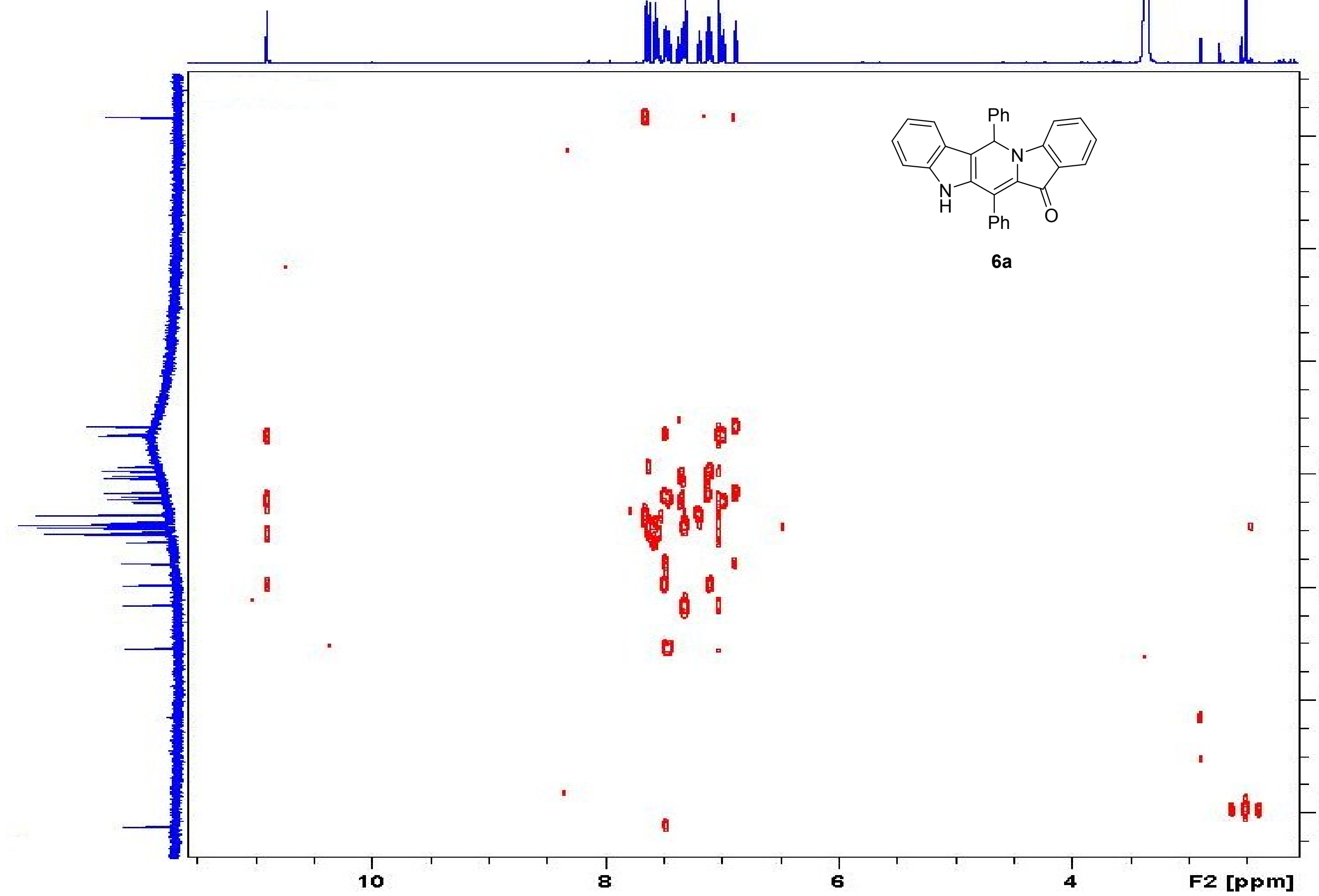

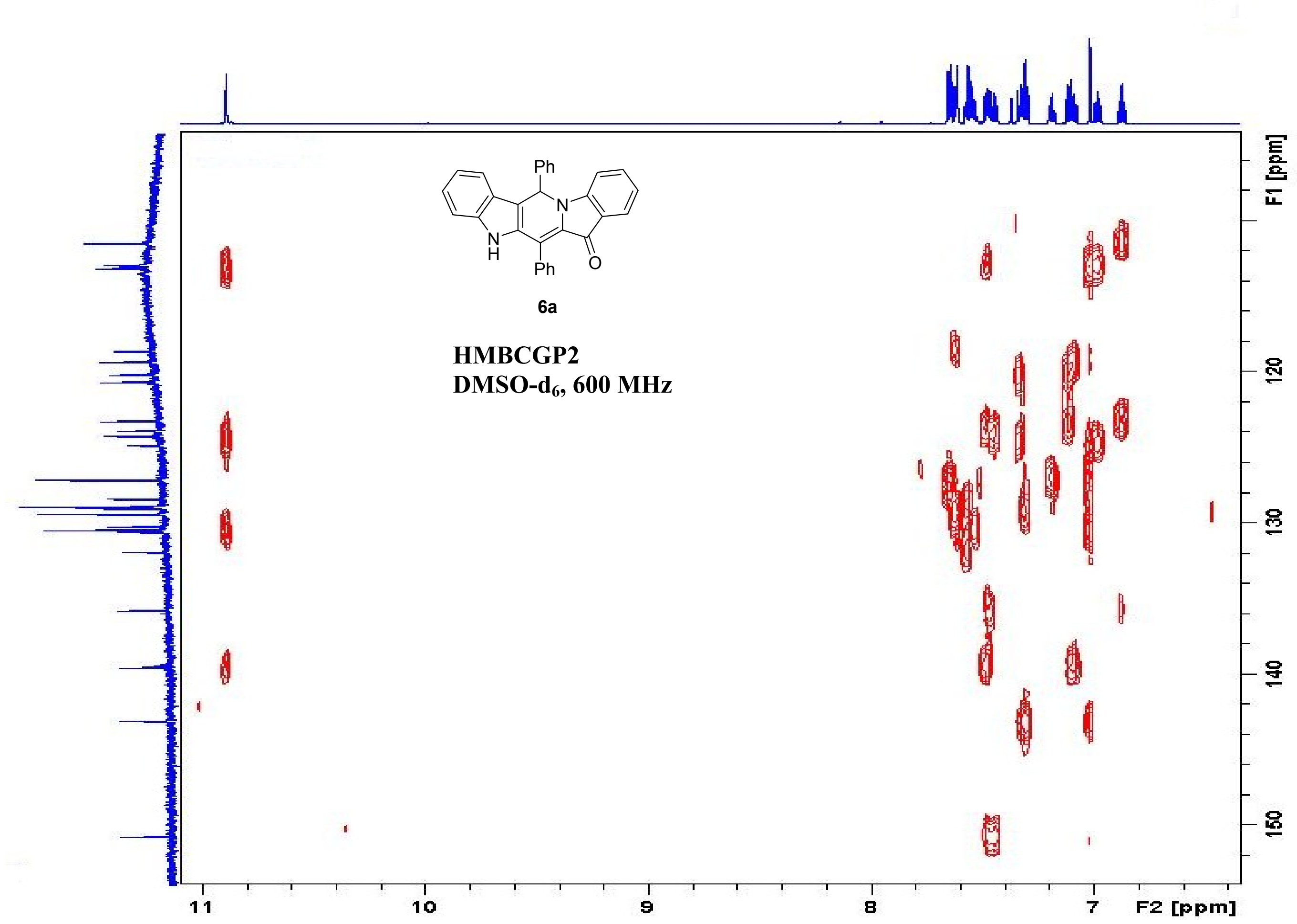

Ph
N
NH
Ph
O
6a
HMBCGP2
DMSO-d6, 600 MHz
F1 [ppm]
120
130
140
150
11
10
9
8
7
F2 [ppm]

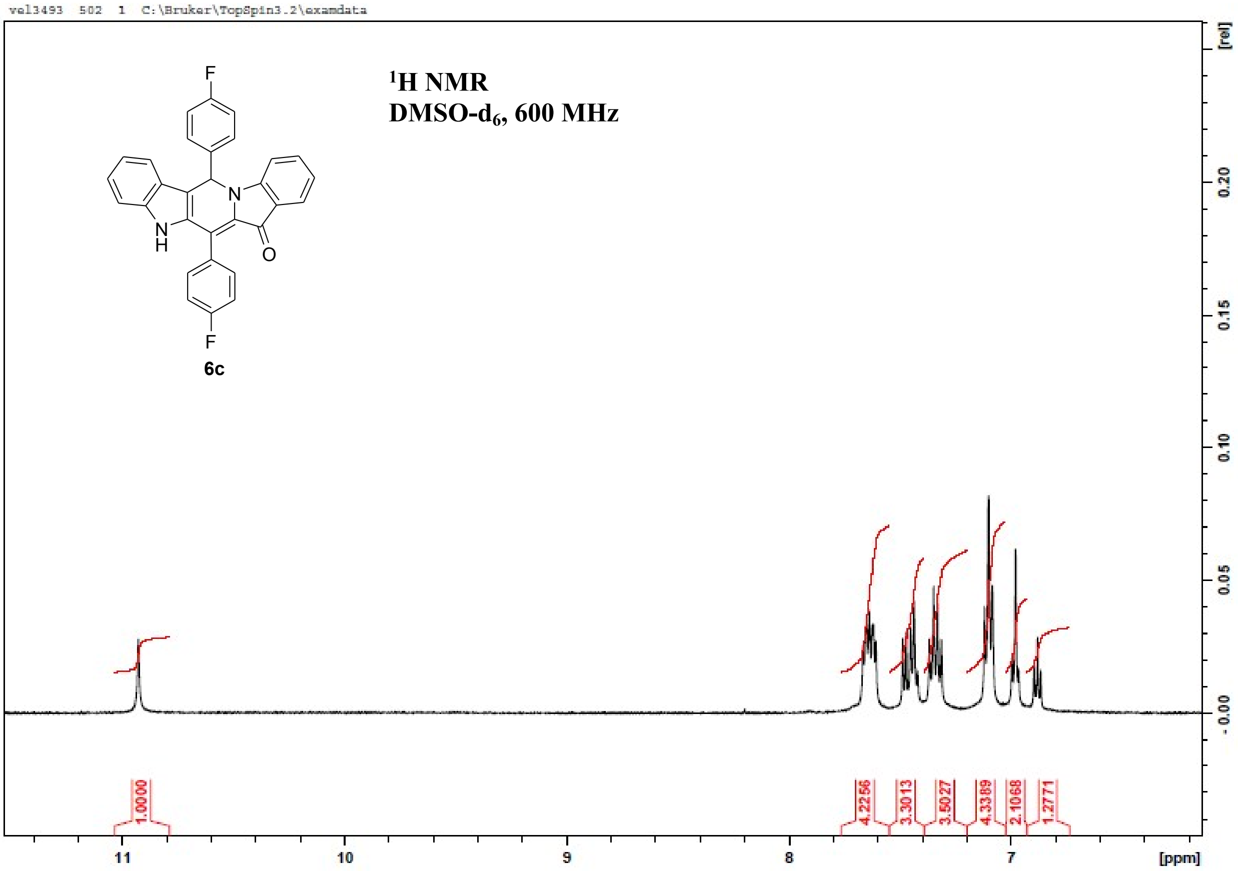
vel3493 502 1 C:\Bruker\TopSpin3.2\examdata
F
N
N
H
O
F
6c
^{1}H NMR
DMSO-d$_6$, 600 MHz
1.0000
4.2256
3.3013
3.5027
4.3389
2.1068
1.2771
11
10
9
8
7
[ppm]
[rel]
0.20
0.15
0.10
0.05
- 0.00

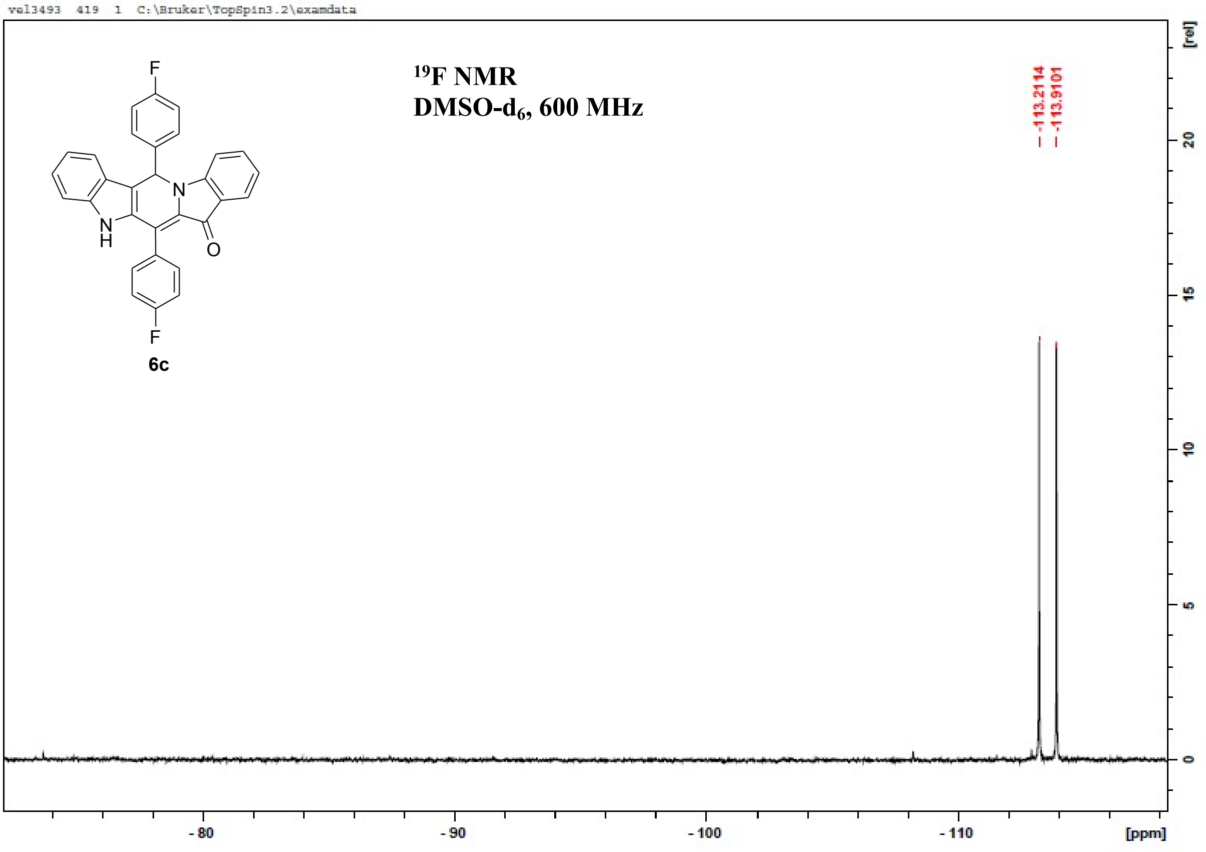
vel3493 419 1 C:\Bruker\TopSpin3.2\examdata
F
N
N
H
O
F
6c
^{19}F NMR
DMSO-d$_6$, 600 MHz
-113.2114
-113.9101
- 80
- 90
- 100
- 110
[ppm]
[rel]
20
15
10
5
0

COSY
DMSO-d$_6$, 600 MHz

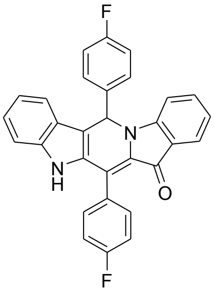

6c

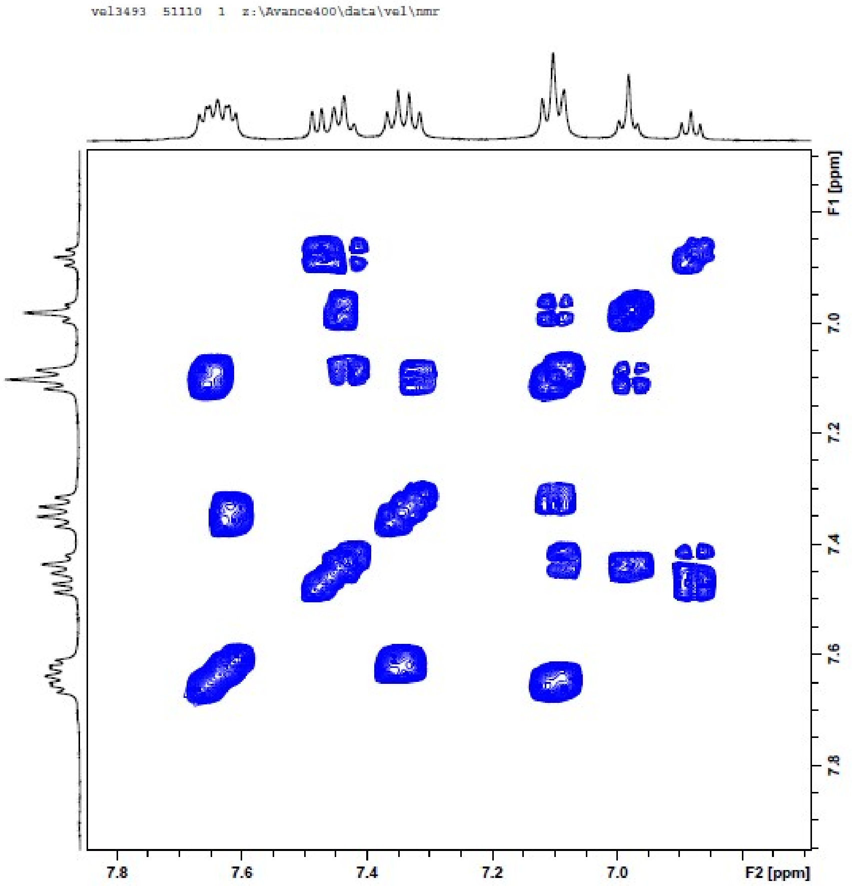

HMBC
DMSO-d$_6$, 600 MHz

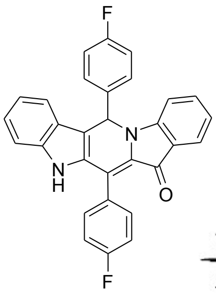

6c

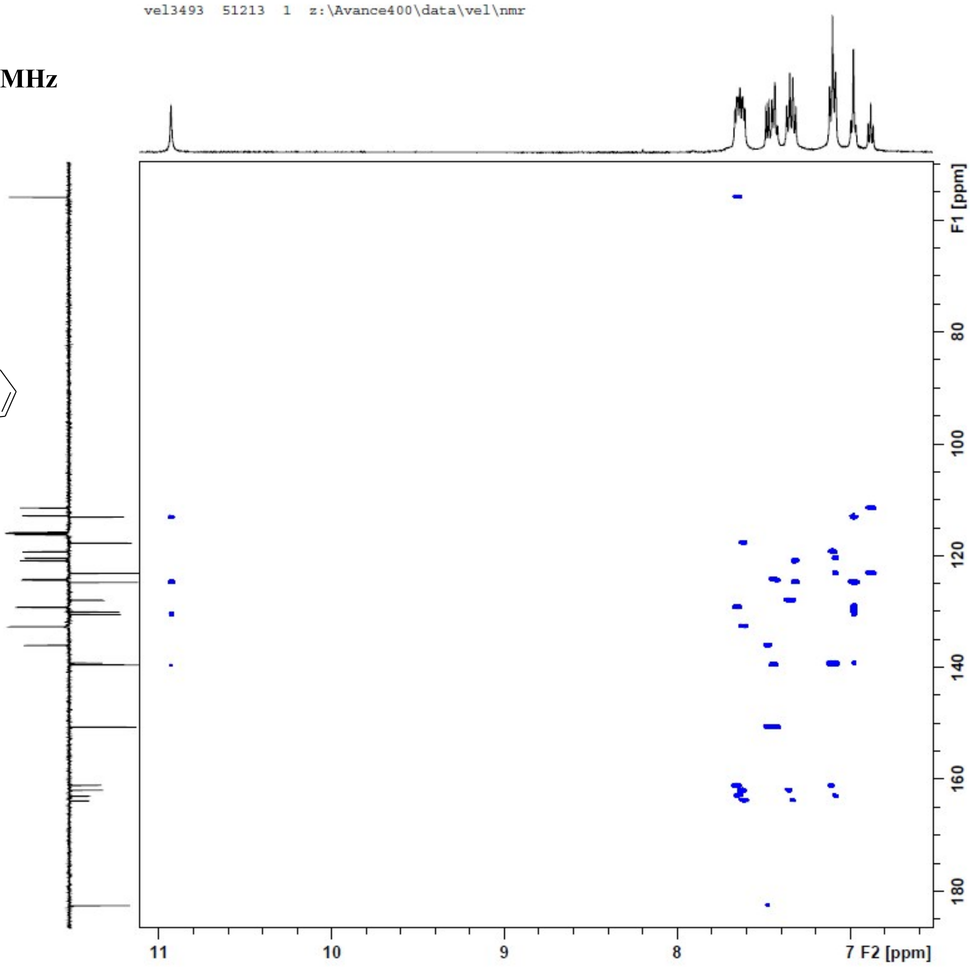

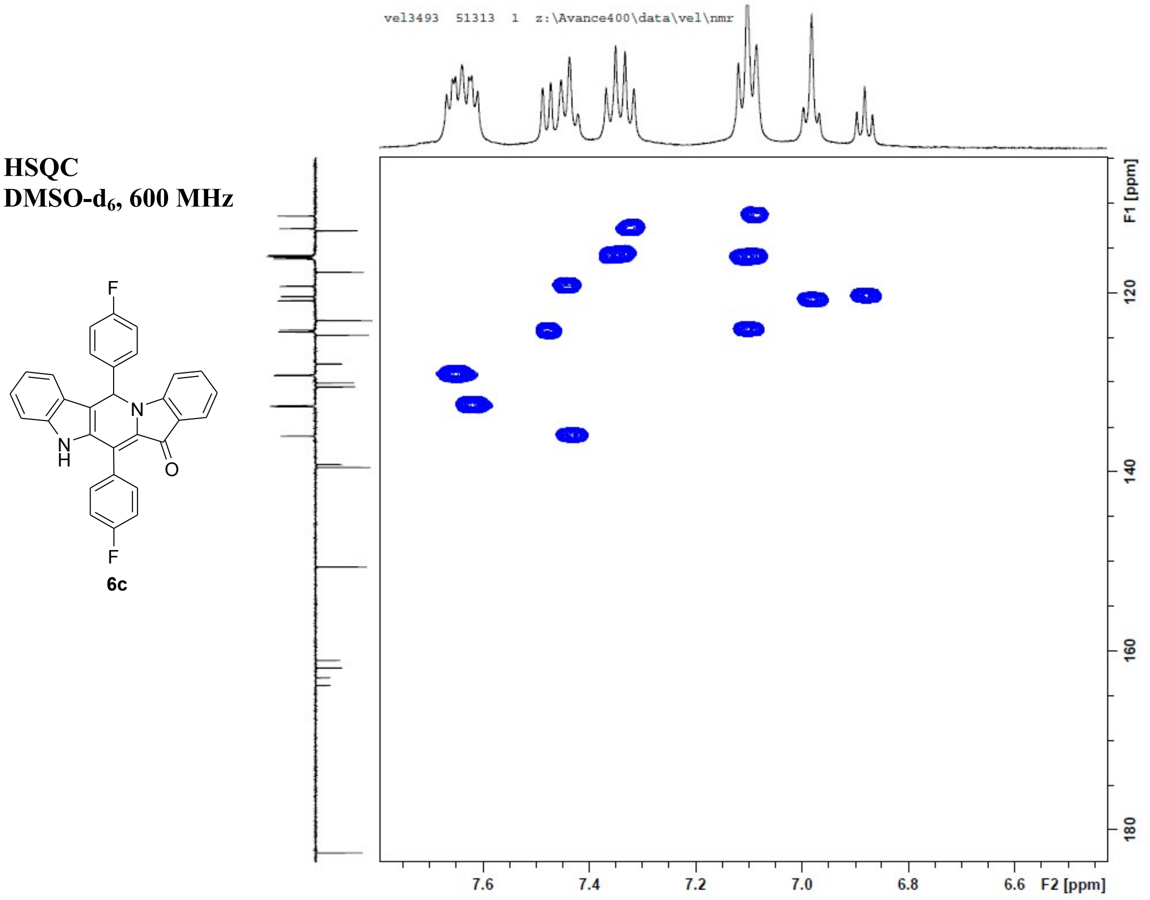

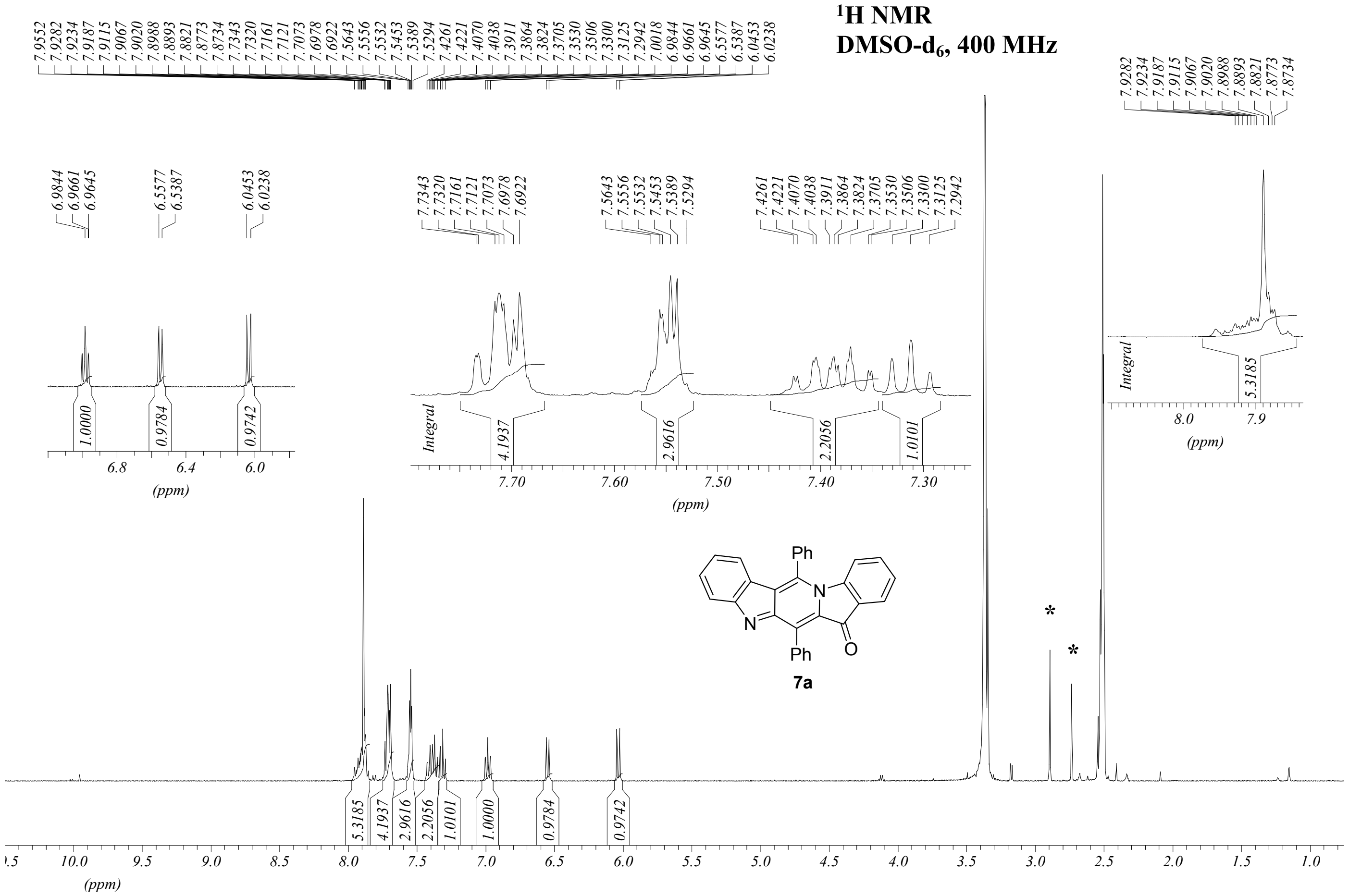

*) signals from DMFA

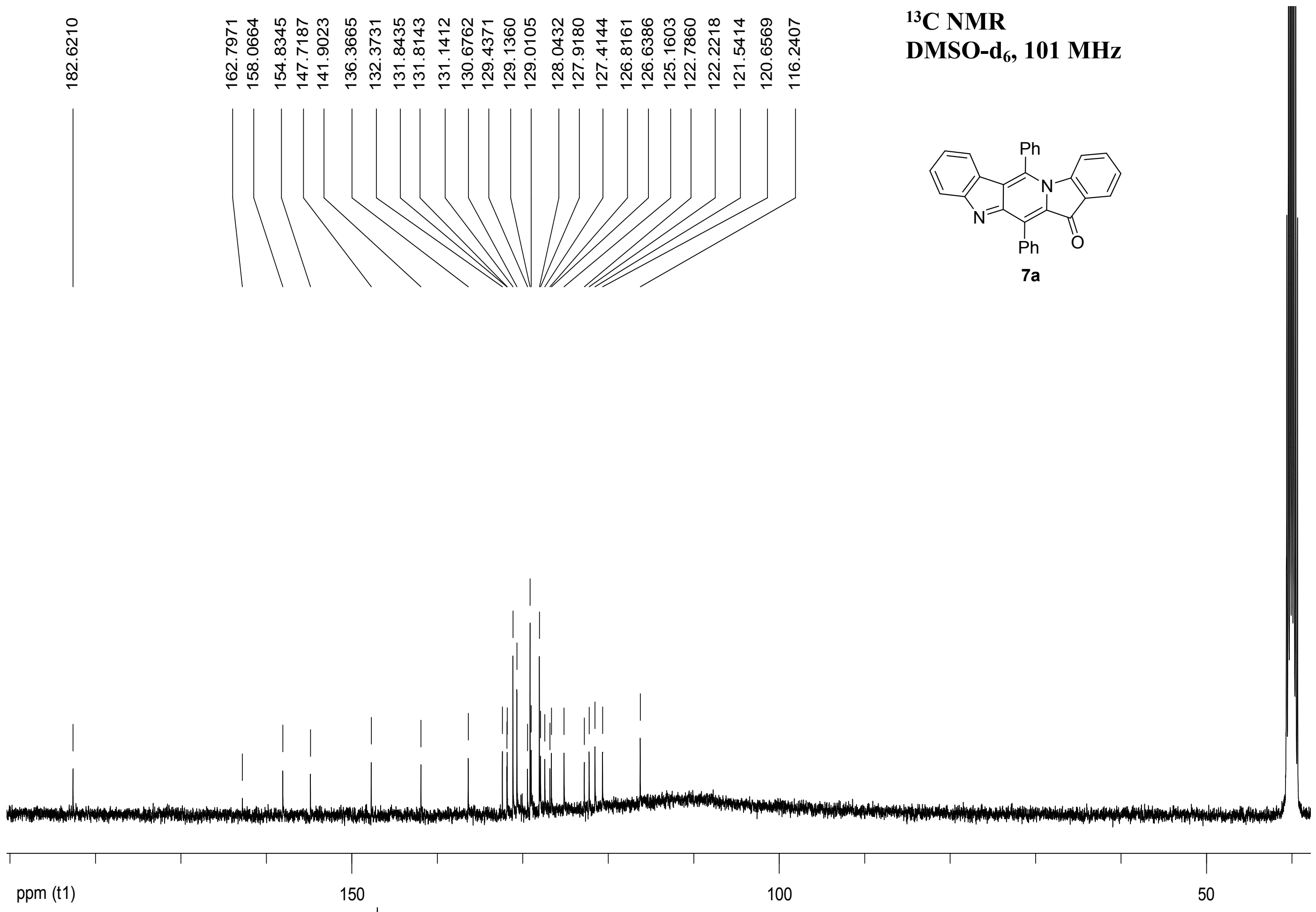
182.6210
162.7971
158.0664
154.8345
147.7187
141.9023
136.3665
132.3731
131.8435
131.8143
131.1412
130.6762
129.4371
129.1360
129.0105
128.0432
127.9180
127.4144
126.8161
126.6386
125.1603
122.7860
122.2218
121.5414
120.6569
116.2407
^{13}C NMR
DMSO-d$_6$, 101 MHz
Ph
N
N
Ph
O
7a
ppm (t1)
150
100
50

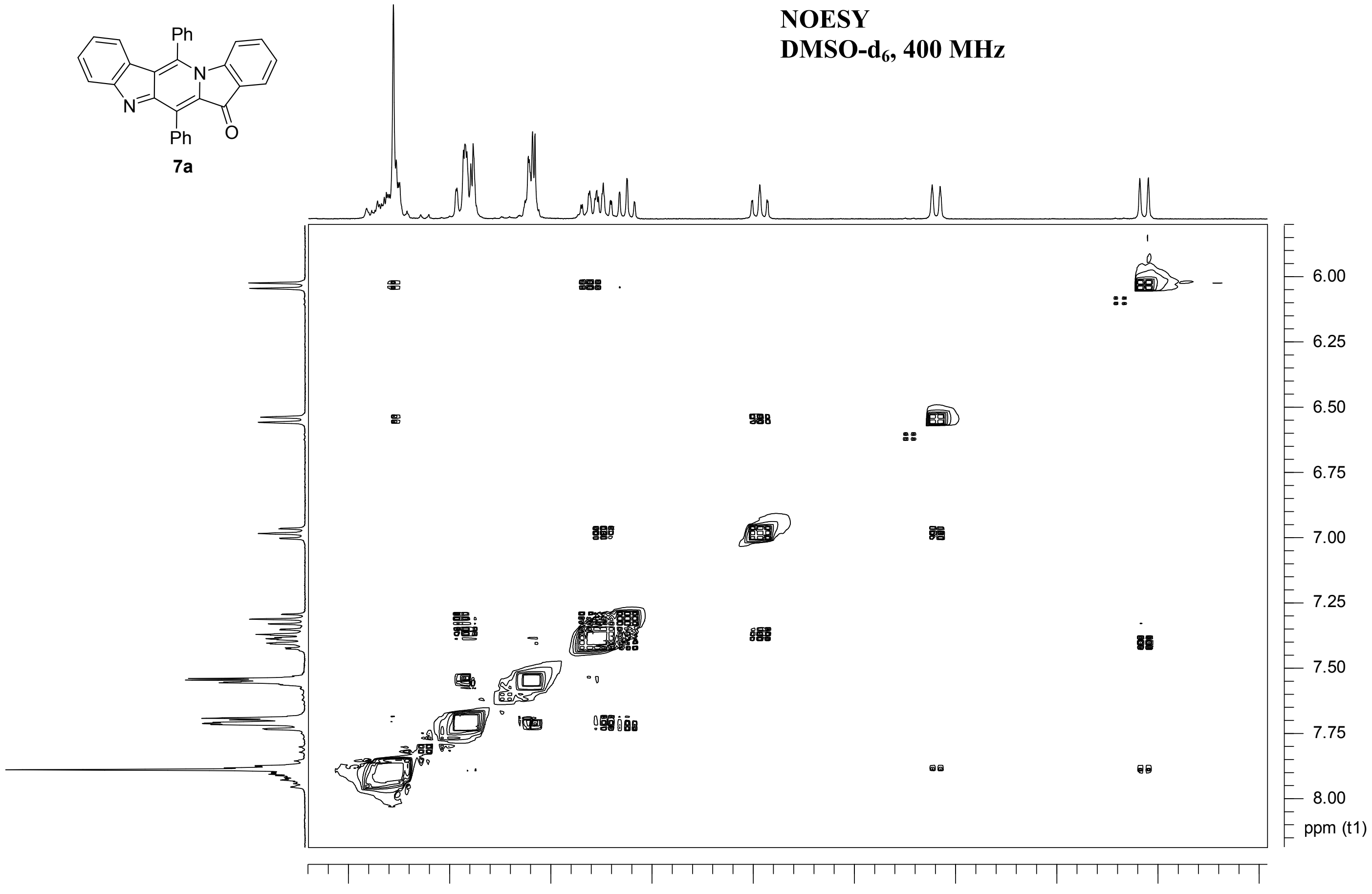
NOESY
DMSO-d$_6$, 400 MHz
Ph
N
N
Ph
O
7a
6.00
6.25
6.50
6.75
7.00
7.25
7.50
7.75
8.00
ppm (t1)
8.00
7.75
7.50
7.25
7.00
6.75
6.50
6.25
6.00
ppm (t2)

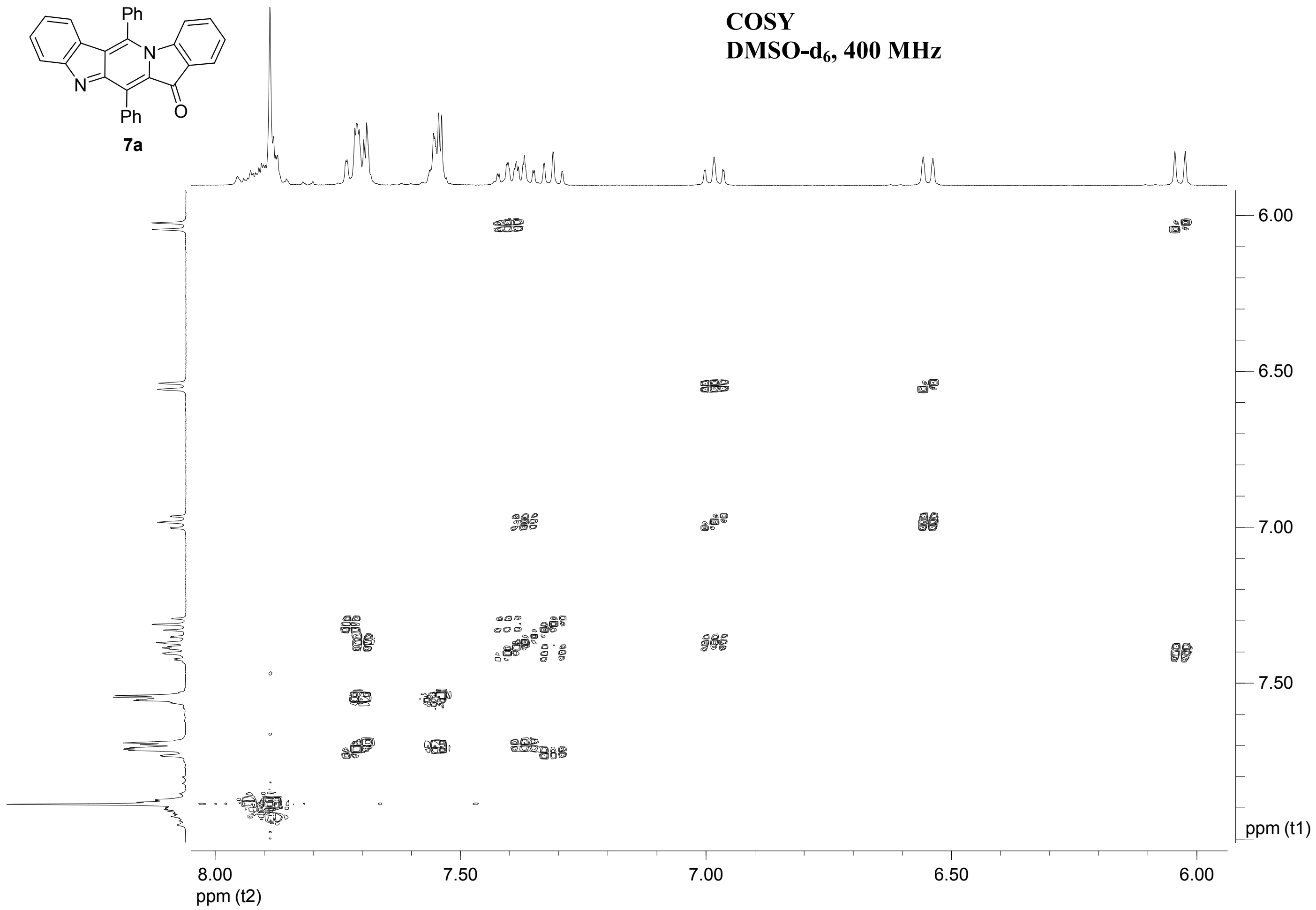
COSY
DMSO-d6, 400 MHz
Ph
N
N
Ph
O
7a
6.00
6.50
7.00
7.50
ppm (t1)
8.00
7.50
7.00
6.50
6.00
ppm (t2)

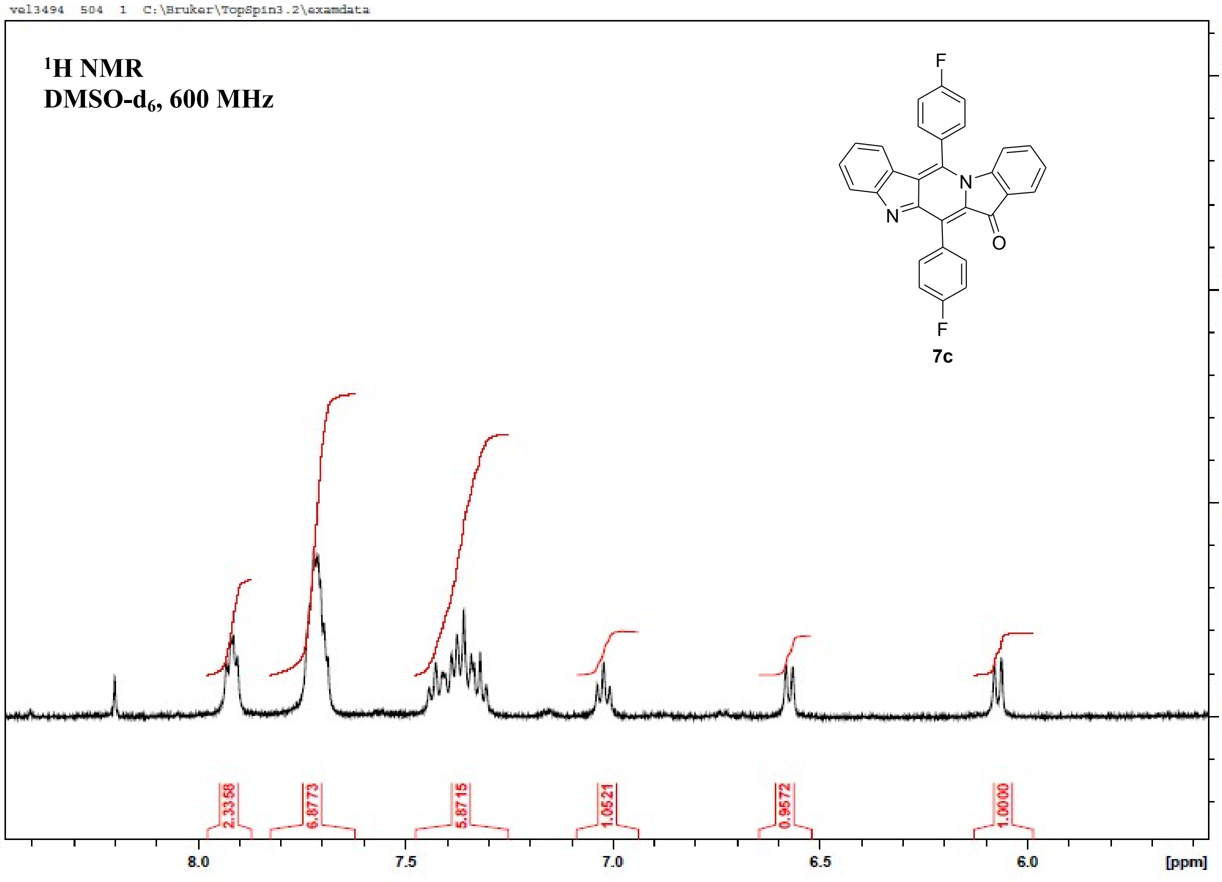
vel3494 504 1 C:\Bruker\TopSpin3.2\examdata
1H NMR
DMSO-d6, 600 MHz
F
N
N
O
F
7c
2.3358
6.8773
5.8715
1.0521
0.9572
1.0000
8.0
7.5
7.0
6.5
6.0
[ppm]
0.03 [rel]
0.02
0.01
0.00

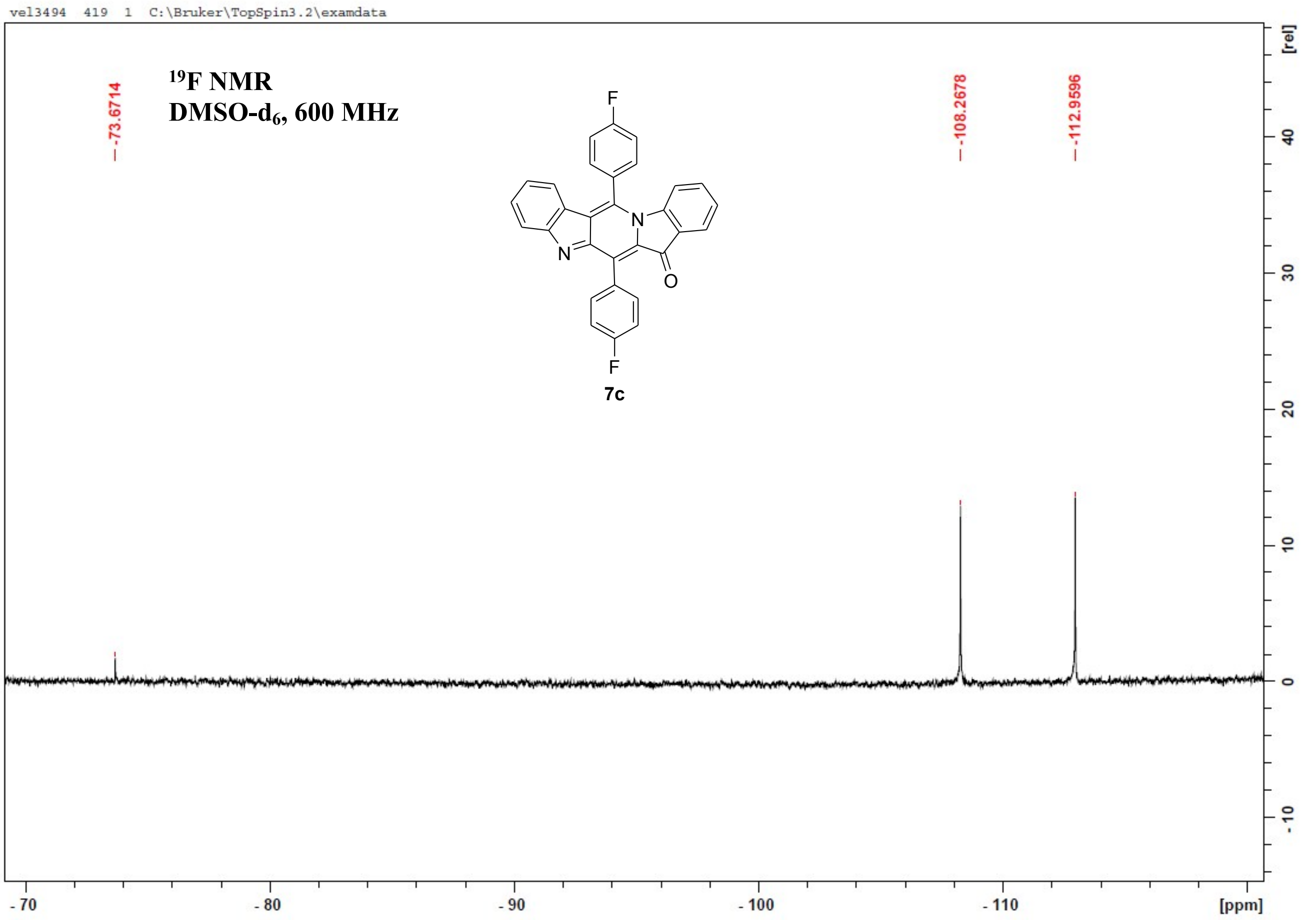
vel3494 419 1 C:\Bruker\TopSpin3.2\examdata
19F NMR
DMSO-d6, 600 MHz
--73.6714
--108.2678
--112.9596
F
N
N
O
F
7c
[rel]
[ppm]

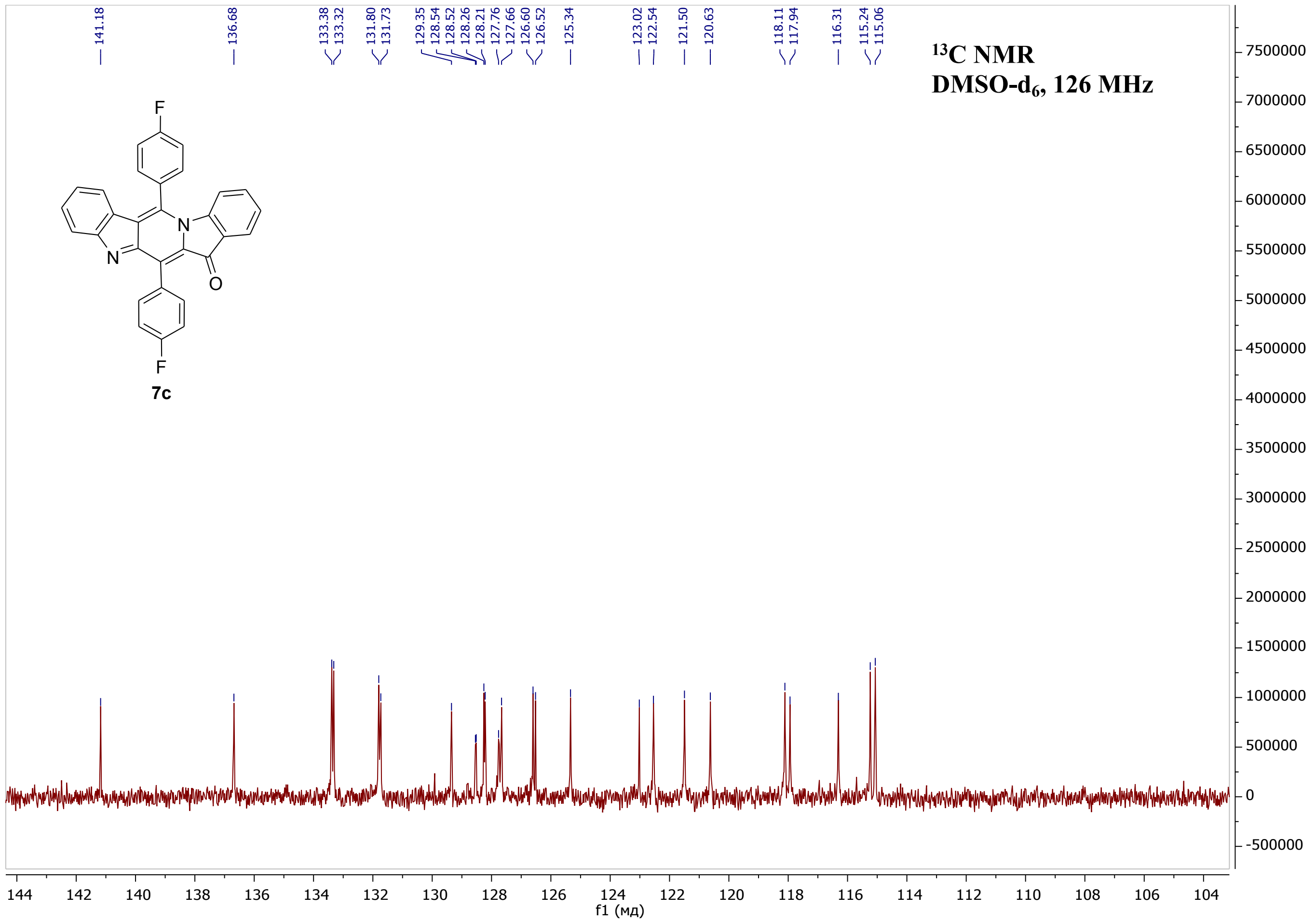
141.18
136.68
133.38
133.32
131.80
131.73
129.35
128.54
128.52
128.26
128.21
127.76
127.66
126.60
126.52
125.34
123.02
122.54
121.50
120.63
118.11
117.94
116.31
115.24
115.06
13C NMR
DMSO-d6, 126 MHz
F
N
N
O
F
7c
f1 (мд)

COSY
DMSO-d$_6$, 600 MHz

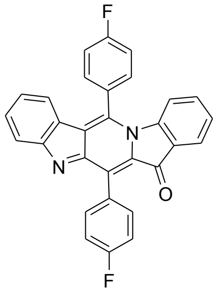

7c

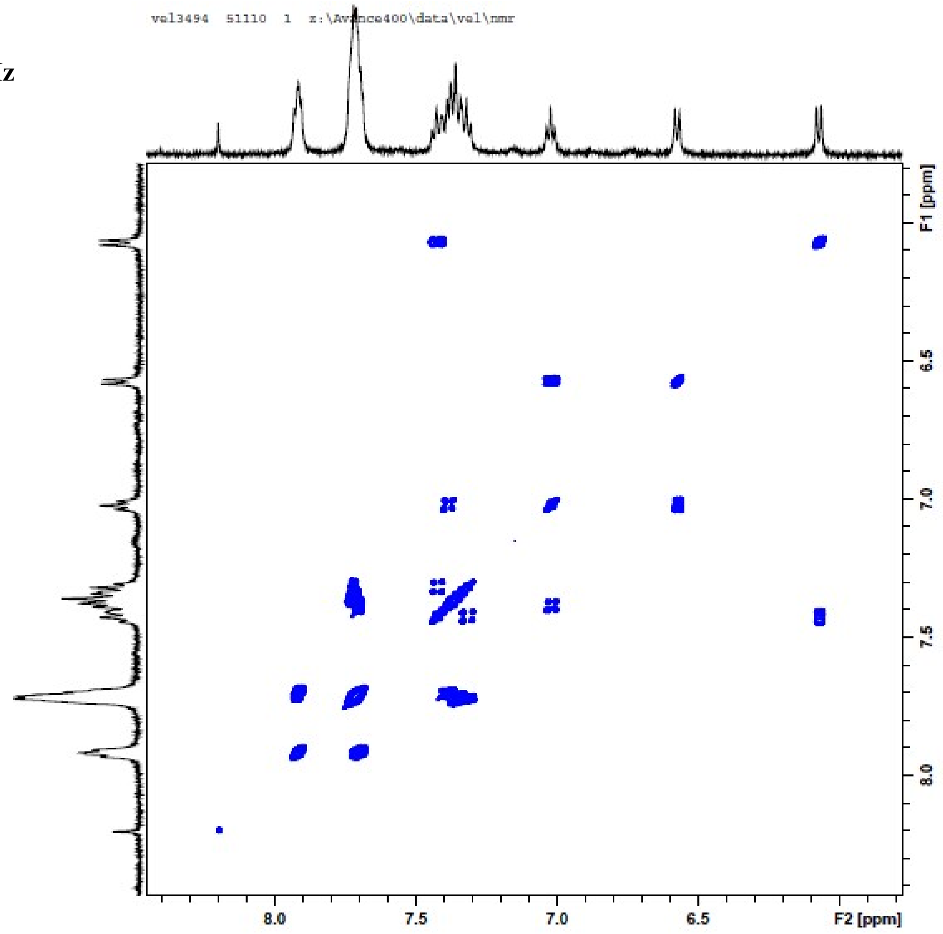

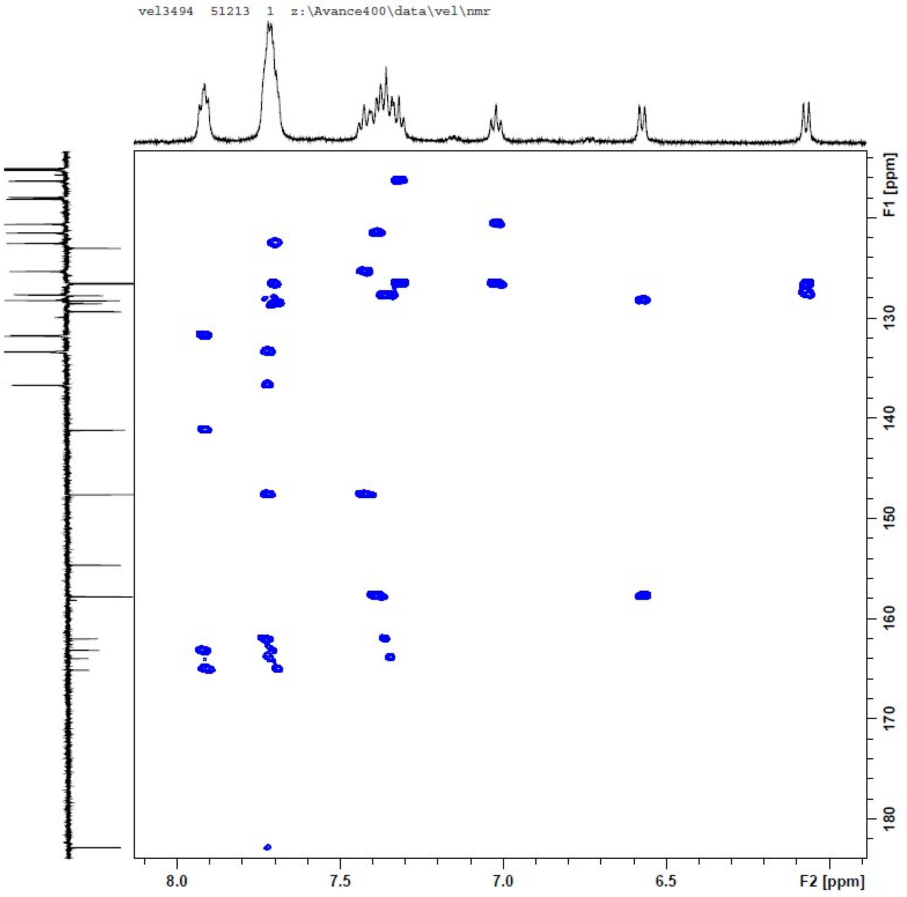

HMBC
DMSO-d$_6$, 600 MHz

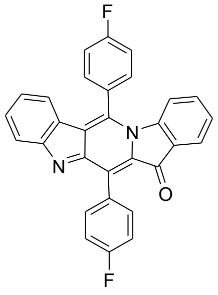

7c

4. X-ray diffraction

X-ray diffraction experiments were carried out at 120 K with a Bruker SMART APEX2 CCD diffractometer for the compound **3** and with a Bruker SMART 1000 CCD diffractometer for all others, using graphite monochromated Mo-Kα radiation (lλ = 0.71073 Å, ω-scans). The structures were solved with the ShelXS[5] structure solution program using Direct methods and refined with the olex2.refine[6] refinement package using Gauss-Newton minimisation. Hydrogen atoms of the NH and OH groups in **2a** and **3** were found in difference Fourier synthesis while the positions of other hydrogen atoms were calculated. All of them were refined in the isotropic approximation within the riding model. Crystal data and structure refinement parameters for **2a**, **3** and **7** are given in Table S1.

All calculations were performed using the SHELXTL[5] and Olex2[6] software. CCDC 1947196 (for **2a**), 1947197 (for **3**) and (for **7**) contain the supplementary crystallographic data for this paper.

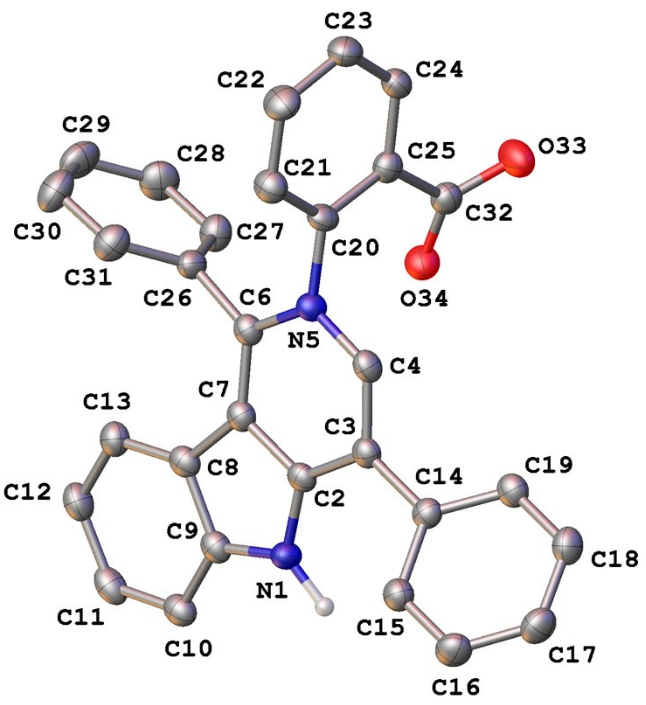

Fig. S1. General view of the compound **2a** in representation of atoms *via* thermal ellipsoids at 50% probability level. Solvate methanol molecules and hydrogen atoms except belonging to the NH group are omitted for clarity.

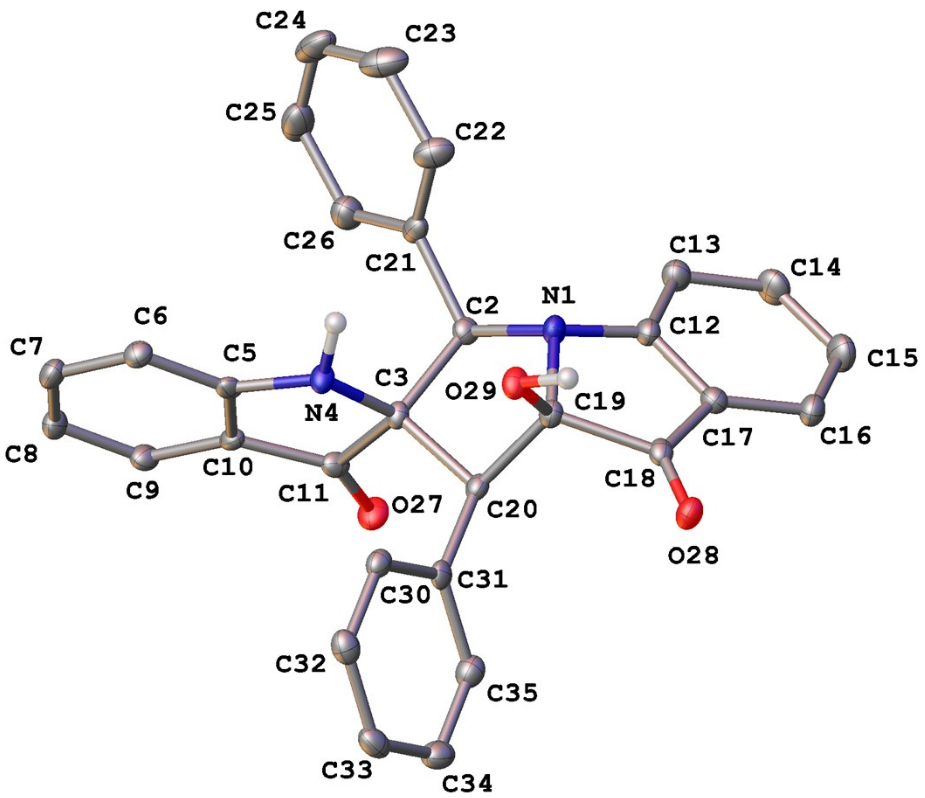

Fig. S2. General view of the compound **3** in representation of atoms *via* thermal ellipsoids at 50% probability level. Hydrogen atoms except those of NH and OH groups are omitted for clarity.

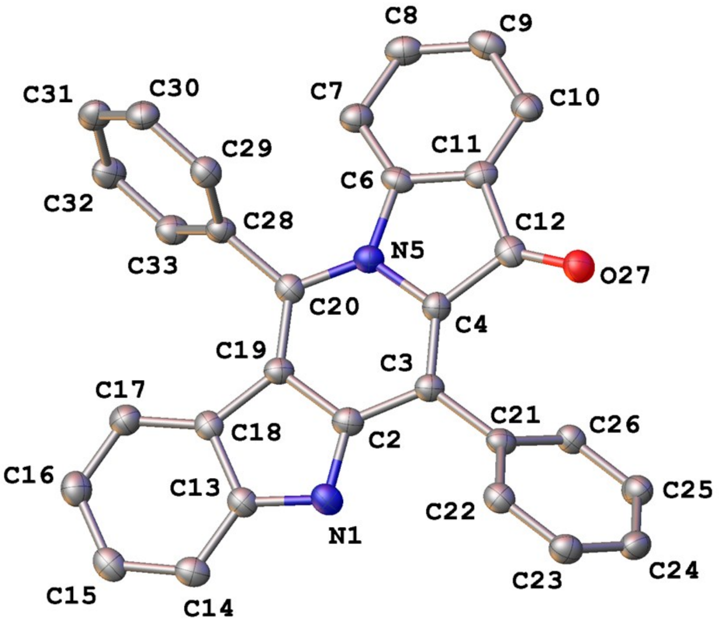

Fig. S3. General view of one of the two symmetry-independent molecules of **7** in representation of atoms *via* thermal ellipsoids at 50% probability level. Hydrogen atoms are omitted for clarity.

Table S1. Crystal data and structure refinement parameters for **2a**, **3** and **7**.

	**2a**	**3**	**7**
Empirical formula	$C_{32}H_{28}N_2O_4$	$C_{30}H_{22}N_2O_3$	$C_{30}H_{18}N_2O$
Formula weight	504.59	458.52	422.46
Crystal system	Triclinic	Monoclinic	Triclinic
Space group	P-1	$P2_1$	P-1
Z	2	2	4
a, Å	8.9151(10)	11.3360(8)	9.8297(9)
b, Å	9.6712(11)	6.3863(4)	11.1714(10)
c, Å	15.8118(17)	15.8956(11)	20.2818(18)
α, °	72.760(3)	90	100.752(2)
β, °	83.325(3)	95.678(2)	98.996(3)
γ, °	76.504(3)	90	106.440(2)
V, $Å^3$	1264.5(2)	1145.12(13)	2046.3(3)
D_{calc} (g cm^{-1})	1.325	1.330	1.371
Linear absorption, μ (cm^{-1})	0.88	0.87	0.84
F(000)	532	480	880
$2\theta_{max}$, °	52	58	56
Reflections measured	14904	13219	20875
Independent reflections	4953	6010	9818
Observed reflections [$I > 2\sigma(I)$]	2543	4389	4536
Parameters	356	316	595
R1	0.0592	0.0521	0.0468
wR2	0.1645	0.1016	0.0800
GOF	0.875	1.034	1.006
$\Delta\rho_{max}$/ $\Delta\rho_{min}$ (e $Å^{-3}$)	0.787/-0.575	0.366/-0.360	0.720/-0.204

5. References

1. M. Hooper and W. N. Pitkethly, *J. Chem. Soc., Perkin 1.,* 1972, 1607-1613.

2. V. S. Velezheva, P. J. Brennan, V. Yu. Marshakov, D. V. Gusev, I. N. Lisichkina, A. S. Peregudov, L. N. Tchernousova, T. G. Smirnova, S. N. Andreevskaya and A. E. Medvedev, *J. Med. Chem.,* 2004, **47**, 3455-3461.

3. W. Zhang and M.-L. Go, *Bioorg. Med. Chem.,* 2009, **17**, 2077-2090.

4. J. H. Gong, K. Y. Lee, J. S. Son and J. N. Kim, *Bull. Korean. Chem. Soc*., 2003, **24**, 507-510.

5. G. M. Sheldrick. *Acta Crystallogr A.,* 2008, **64**, 112-122.

6. L. J. Bourhis, O. V. Dolomanov, R. J. Gildea, J. A. K. Howard and H. Puschmann, *Acta Crystallogr. A Found Adv.*, 2015, **71**, 59-75.

7. K. B. Majorov, I. V. Lyadova, T. K. Kondratieva, E. B. Eruslanov, E. I. Rubakova, M.O. Orlova, V.V. Mischenko and A. S. Apt, *Infect. Immun*., 2003, **71**, 697–707.

8. B. V. Nikonenko, A. Kornienko, K. Majorov, P. Ivanov, T. Kondratieva, M. Korotetskaya, A. S. Apt, E. Salina and V. Velezheva, 2016, **60**, 6422-6424.
